# Friendship segregation and class composition in schools: A systematic analysis of the role of attribute consolidation

**DOI:** 10.1371/journal.pone.0339581

**Published:** 2025-12-31

**Authors:** Susanne Böller, Hanno Kruse

**Affiliations:** Department of Sociology, University of Bonn, Bonn, Germany; Sun Yatsen University, CHINA

## Abstract

In schools around the world students are sorted to different groups, courses, or classes. In increasingly diverse societies, such sorting practices play an important role to social cohesion. They set the structural conditions for students to form friendships across demographic divides. This article highlights the importance of attribute consolidation in learning contexts, the extent to which demographic categories align or cross-cut each other, a compositional feature largely overlooked in practical school settings. In Study 1, a comprehensive analysis of the association of attribute consolidation and friendship segregation in 524 school classes in Germany, the Netherlands, and Sweden reveals the outstanding significance of *gender consolidation*. This means that friendships are far more segregated along an attribute when it aligns with gender in a classroom than when it coincides with other attributes, such as socio-economic status or place of residence. Based on this finding, Study 2 uses simulations to examine the potential effects of class-placement strategies that reduce gender consolidation. The results suggest that, relative to observed class placement in Germany, the Netherlands, and Sweden, class placement that minimizes gender consolidation can decrease friendship segregation in that it reduces students’ share of ingroup friends by 4–7 percentage points. More generally, the potential impact of paying attention to gender consolidation in class placement is the largest in diverse schools, which are mostly located in diverse countries with low levels of between-school segregation.

## Introduction

The increasing diversity of today’s societies [1,2] is often poorly reflected in people’s social relationships, including adolescents’ friendship networks in schools, where segregation along demographic lines persists [3,4]. Because attending diverse schools does not automatically translate into diverse friendships, students often lack out-group friends even if they share a school, with potential implications for their educational opportunities, self-image, intergroup attitudes, and societal cohesion as a whole [5–7].

A central reason why students may fail to form friendships across demographic divides within their schools may be the lack of foci for intergroup encounters [8]. Various sorting practices within schools can affect intergroup contact, with ability grouping and tracking receiving considerable public and academic attention [9,10]. The most direct and immediate impact on intergroup encounters, however, occurs when school administrators directly target the demographic makeup of a learning environment, for example, by balancing students of different ethnic/racial groups across classes in a given cohort. Class placement criteria beyond academic performance are prominently used in schools, targeting more than 90% of students in OECD countries in 2022 [11]. In this article, we contribute to the question of how school administrators can best use this leverage over class placement to facilitate diverse friendships among adolescents.

When it comes to creating learning contexts that provide more or less favorable opportunities for diverse friendships, most attention is paid to the role of relative group size [3,12–15], with the underlying idea that more diverse contexts provide more opportunities for intergroup encounters [16]. However, another critical compositional feature of school classes has received little attention: attribute consolidation—the extent to which demographic categories align (e.g., all boys in a class are Muslim while all girls are Christian) or cross-cut each other (e.g., gender and religion are unrelated) [16]. Previous research suggests that reducing attribute consolidation in class placement may be beneficial for friendship diversity in schools. Importantly, these insights are currently limited to ethno-racial divides and their alignment with socio-economic status (SES) [3,17] and gender [18], thus lacking a comprehensive analysis of consolidation effects across a wide range of demographic attributes. From a practical perspective, this is necessary because school administrators face various, often locally specific, divides that transcend students’ countries of origin, and it is unclear which consolidated attributes require the most attention to effectively address them. From a sociological perspective, this is also compelling, as an intersectional approach suggests that some combinations of attributes may carry greater salience in friendship formation when consolidated than others [17,19]. In this light, it remains an open question whether attributes that are generally the strongest predictors of adolescent friendship choices (such as gender) will also exert the strongest consolidation effects when combined with other attributes.

To systematically examine the role of attribute consolidation in friendship segregation in learning environments, we conduct a series of two studies. In *Study 1*, we conduct a comprehensive analysis of the relationship between attribute consolidation and friendship segregation in 524 school classes across Germany, the Netherlands, and Sweden, using data from the “Children of Immigrants Longitudinal Survey in Four European Countries (CILS4EU)”. We examine a total of 42 attribute combinations, including students’ socio-economic background, educational background, country of origin, religion, language spoken at home, place of residence, and gender. This selection captures attributes that are central to ongoing debates about social cohesion and inequality, as well as those that hold practical relevance for school personnel involved in class placement decisions.

Building on these findings, *Study 2* explores the practical significance of attribute consolidation. Specifically, we examine whether accounting for the most impactful forms of attribute consolidation identified in Study 1 could hypothetically affect friendship segregation. Given that such an impact would depend not only on the observed effect sizes (identified in Study 1) but also on the demographic makeup of the student cohort being assigned to classes, we incorporate a wide range of observed cohort compositions into the simulations in Study 2. Through simulations of class placement, we compare counterfactual levels of segregation to observed patterns, first in the three countries under study and then more broadly as a function of school population composition, drawing on a realistic value space from over 16,000 schools in 79 countries. Although the simulations do not account for the practical constraints of implementing class placement strategies that specifically reduce consolidation, they offer a theoretically grounded estimate of the upper bound of friendship segregation reduction achievable under idealized placement scenarios relative to existing practices and cohort structures in schools.

## Study 1. Testing consolidation effects on friendship segregation across different attributes

**Fig 1:**
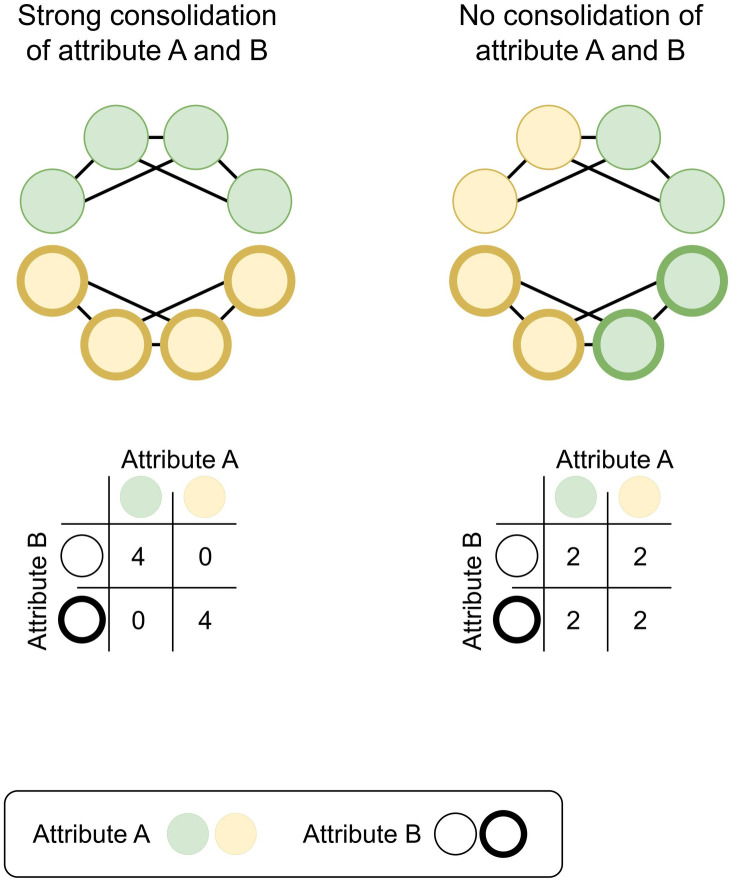
Stylized visualizations of the association between attribute consolidation and friendship segregation. In both classes, friendships are strongly segregated along the lines of attribute **B.** In the left class, attributes A and B are strongly consolidated. Here, the strong friendship segregation along the lines of attribute B translates to segregation along the lines of attribute **A.** In the right class, attributes A and B are not consolidated. In this class, the friendship networks are diverse with respect to attribute A, irrespective of the strong friendship segregation in attribute **B.**

Before turning to prior research and its gaps regarding the effects of attribute consolidation on friendship segregation, we begin by illustrating the core idea with a simplified example. Fig. 1 shows two stylized school classes. In both classes, friendships are strongly segregated along the lines of attribute B. However, the classes differ in the extent to which attribute A and B are consolidated. The class on the left is characterized by a strong attribute consolidation: All students who share attribute A also share attribute B (and vice versa). For example, if attribute A is SES (thin border = low SES, thick border = high SES) and attribute B is gender (green = boys, yellow = girls), then all four boys have a low SES and all four girls a high SES. Here, a tendency towards same-gender friends automatically produces SES segregation. In contrast, attributes A and B are completely unconsolidated in the class on the right: Only half of the students who share attribute A also share attribute B (and vice versa). Using the same example, two of the boys and two of the girls have a high SES, while the other two boys/ girls have a low SES. In this class, the friendship networks are diverse with respect to students’ SES, regardless of the strong gender segregation. In this study, we examine how the stylized relationship – between the consolidation of attributes A and B and friendship segregation along attribute A – varies depending on which attributes are chosen as A and B.

In sociology, the concept that attribute consolidation undermines social cohesion was first introduced by Simmel [20], drawing on various historical examples. Building on this insight, seminal work on the structural predictors of intergroup relations [16,21] later argued that attribute consolidation, or “the concomitant variation of several kinds of social differences” [21: 99] impedes intergroup relations. Empirically, it demonstrated the detrimental effects of attribute consolidation on intergroup relations in the context of intermarriage in 125 U.S. metropolitan areas.

Related research strands have emerged across the social sciences. In management research, the consolidation of multiple attributes within work teams – often conceptualized as demographic faultlines [22] – has been shown to reduce group performance and satisfaction while increasing relationship and task conflict [23,24]. In political science, studies indicate that societies in which ethnicity is not consolidated with religion, socio-economic status and geographic region are less likely to experience civil war [25–28].

Applied to the school context, attribute consolidation has been examined with respect to its effects on friendship segregation, both in U.S. high schools [3] and in secondary schools across several European countries [17,18]. In addition to differing national contexts, these studies differ in their operationalizations of segregation. Two studies include analyses of *gross* segregation, that is, the observed level of friendship segregation in a network, irrespective of the mechanisms producing it [3,18]. In contrast, Zhao [17] analyses *net* segregation isolating how attribute consolidation shapes preferences for same-ethnic friends after accounting for other tie-generating mechanisms. In the present study, we adopt the former approach and focus on gross friendship segregation, as our interest lies in the overall extent to which attribute consolidation is associated with friendship segregation, regardless of whether this association operates through individual preferences or opportunity-based mechanisms of tie formation.

Importantly, previous research has concentrated on friendship segregation along ethno-racial lines. These studies show that friendships are more segregated by ethnicity or race when these attributes are consolidated with gender [18] or socioeconomic status [3]. As we argue below, there are compelling reasons to broaden this scope by systematically comparing consolidation effects across different combinations of attributes. This extension pertains both to the outcome variable (e.g., whether consolidation similarly affects ethnic versus religious segregation) and to the consolidating attribute (e.g., whether ethnic friendship segregation is more strongly shaped by the consolidation of ethnicity with gender or SES).

As shown in Fig 1, consolidation mechanically transfers friendship segregation in attribute B to friendship segregation in attribute A. Different mechanisms may underly friendship segregation [15], with homophily (i.e., a preference for similar others) [29] and propinquity (i.e., segregated meeting opportunities) [8] being the most prominent ones. Any attribute B that induces homogeneous friendship choices (through any of these mechanisms) can reinforce friendship segregation along the lines of attribute A if the attributes are consolidated. Previous studies suggest the existence of segregating tendencies in adolescents’ friendship networks along various demographic dimensions, such as socio-economic background, educational background, country of origin, religion, language spoken at home, place of residence, and gender [4,15,30–33]. Thus, all of these attributes could impose consolidation effects on friendship segregation along the lines of any attribute A.

*H1: Friendship segregation along the lines of an attribute A is higher in school classes with a stronger consolidation of attributes A and B, with attributes A and B being defined as any of the 42 combinations of students’ socio-economic background, educational background, country of origin, religion, language spoken at home, place of residence, and gender.*

Beyond the general expectation of positive consolidation effects on friendship segregation, little is known about how these effects vary across different combinations of attributes. Kroneberg et al. [18] find that while the consolidation of ethnicity and gender increases friendship segregation along ethnic lines, it has nearly no effect on gender segregation. This asymmetric finding is consistent with the consolidation effect depicted in Fig 1, with ethnicity taken as attribute A and gender as attribute B: While both networks are strongly gender segregated, ethnic segregation is contingent on the degree to which ethnicity is consolidated with gender in the respective classroom. Gender is particularly salient and important to adolescents [34]. While the salience of attributes such as religion, spoken language, or place of residence may vary across learning environments, gender consistently plays a prominent role. A systematic review of 51 studies from 28 countries worldwide shows that gender segregation in school friendship networks is a consistent global phenomenon (see S1 Table in the Appendix). Comparative research further shows that gender exhibits the strongest segregating patterns in friendship networks, while other attributes such as students’ country of origin, religion, or socio-economic status exhibit weaker segregating tendencies [4,30–33].

At the same time, there is evidence suggesting that certain combinations of demographic attributes can have effects on friendship segregation that go beyond what would be expected from their separate, additive influences. Zhao [17], for instance, demonstrates that the consolidation of ethnic origin and socio-economic status increases the salience of ethnicity in friendship choices, whereas a similar consolidation between ethnic origin and gender does not yield the same effect. This finding resonates with intersectionality theory, which posits that specific intersections of social categories can produce unique identities and forms of social relevance that cannot be reduced to their component attributes in isolation [19]. Consequently, it cannot be assumed that gender—although generally the strongest predictor of adolescent friendship choices—will necessarily exert the strongest consolidation effects when combined with other attributes. Instead, certain intersections may give rise to particularly salient social distinctions that surpass the influence of gender alone. In light of this open question, we test the following hypothesis:

*H2: The positive association between the consolidation of attribute A and B and friendship segregation along the lines of attribute A is stronger when attribute B is gender rather than students’ socio-economic background, educational background, country of origin, religion, language spoken at home, or place of residence.*

### Data and methods

To examine consolidation effects on friendship segregation across attributes with different segregating power, we use unweighted data from the first wave of the “Children of Immigrants Longitudinal Survey in Four European Countries (CILS4EU)” [35]. While the dataset comprises nationally representative survey responses from students in four different countries, we exclude English schools from the analysis, because instead of classes they organize teaching predominantly in courses, i.e., each student belongs to several subject-specific learning groups. This leaves us with 14,401 students around the age of 14 in 744 school classes at 373 randomly selected secondary schools in Germany, the Netherlands, and Sweden, collected in 2010 and 2011. In all three countries, students were sampled using a stratified, three-stage sampling design: In stage 1, schools were randomly selected with probabilities proportional to their size (oversampling schools with a high proportion of ethnic minority students). In stage 2, two ninth grade classes were randomly selected in each school. In stage 3, all students in the classes were selected. Only students without parental refusal (in Germany, only students with active parental consent) could participate in the study. The CILS4EU data collection received ethical approval from the ethical vetting boards from the Universities of Stockholm, Mannheim, and Oxford. In the Netherlands, ethical approval was not required at the time of data collection, but they followed the same ethical standards as in the other three survey countries. Additional ethical approval for the presented study was not required, as the analysis is based on anonymized secondary data that does not involve direct interaction with human subjects. Access to CILS4EU data can be requested at GESIS Data Archive for the Social Sciences.

We in Study 1 aim to examine how the overall levels of friendship segregation along the outlined seven demographic attributes are affected by consolidation with the remaining other attributes. The seven dependent variables in Study 1 are therefore the *share of ingroup friends* based on the seven different attributes. They are based on sociometric questions about students’ up to five best friends in their school class (“Who are your best friends in class?”). We define the share of ingroup friends as the proportion of nominated friends reporting the same category in a given demographic attribute as the nominating students. To obtain information on the seven selected attributes, we combine survey responses from the students and their parents. *Socio-economic background* is defined as low, medium, or high based on the highest value of the parents’ International Socio-economic Index of Occupational Status (ISEI) [36], with categorical cutoffs following the sample terciles in ISEI values across 79 countries in the PISA 2018 database (see Study 2). We define *educational background* in three categories as primary education or lower, secondary education, or tertiary education based on the highest educational attainment of students’ parents. *Country of origin* is defined based on the countries of birth of the students and their parents. For students born abroad, we define their own country of birth as their country of origin. For students born in the survey country, we use the mother’s country of birth if she was born abroad and the father’s country of birth otherwise [37]. Students could select their religious affiliation from a list or report another religion in an open-ended question, and we combine both types of information to define students’ *religion*. The *language spoken at home* is defined as either the survey country language or students’ second language, based on students’ reports of whether they often or always talk to their family in this second language. In the absence of precise information about where students live, we define their *residential area* based on dyadic reports about classmates who live within five minutes of each other. Each component in this network of residential proximity is considered a residential area in the respective school class. Students’ *gender* was collected as a binary variable so we distinguish between students who identify as boys and those who identify as girls.

In additional analyses we further use metric information on students’ self-evaluated math performance (from 1 = “not well at all” to 5 = “very well”), their school satisfaction (from 1 = “very satisfied” to 10 = “very unsatisfied”), and the number of friends in the same class that were nominated by students when asked to list their five best friends in general. We impute missing values in all variables (except students’ friends and place of residence) ten times using multivariate imputation by chained equations, repeat the following steps for each imputed dataset and combine the results using Rubin’s rules [38]. Supplementary analyses using only complete cases yield substantially similar results (see S2 Table in the Appendix).

Attribute consolidation, our covariate of interest, is a contextual feature that does not vary at the individual level but only between demographic categories within classes. Therefore, we follow previous work [18] and aggregate the *proportion of in-group friends* at the level of groups within classes and use this as our unit of analysis. This means that, for each group in a school class, we calculate the proportion of friendship nominations that group members send to ingroup peers, relative to all nominations that they send to members of their class. A ‘group’ consists of at least three students in the same school class who share the same category for one of the seven specified attributes. For example, in a class with eight students of Turkish origin, ten students of German origin, and one student of Polish origin, we would identify two ethnic groups within the class: Turkish and German.

We use Cramer’s V to measure *attribute consolidation*, following prior research that applies this statistic to assess the association between student attributes in school classes [17,18]. Cramer’s V is based on the chi-squared statistic and well-suited to measure the association between nominal variables with multiple response categories. It ranges from 0 (no overlap) to 1 (perfect overlap) and is defined as

${Consolidation}_{g,c,s}=\;\sqrt{\frac{{\chi^{\text{2}}}_{g,c,s}}{n_{c,s}\;*\;min(\text{1},{CategoriesB}_{c,s}-\text{1})}}$,

for each group g (defined by attribute A, e.g., low, medium, and high when A = SES) in class c in survey country s, where ${\chi^{\text{2}}}_{g,c,s}$ is the chi-squared statistic, $n_{c,s}$ the total number of students in class, and ${CategoriesB}_{c,s}$ the number of categories in attribute B. As we measure consolidation as the overlap between group membership (defined by attribute A) and attribute B, it varies not only across classes but also groups within classes, as indicated by the subscript *g,c,s*. For example, in a class with 10 low-SES boys, 5 medium-SES girls, and 10 high-SES girls, group membership is more strongly consolidated with gender from the perspective of the low-SES students (Cramer’s V = 1) than medium-SES students (Cramer’s V = 0.4).

For each of the seven attributes, we create separate datasets, containing information on each group’s *share of ingroup friends* (see above), the *consolidation* of group membership with each of the other six attributes (see above), the *class size*, the *group size*, the *ingroup-outgroup diversity* (measured as the inverse Herfindahl index of the proportions of in- and outgroup members in class), the *diversity* of the other six attributes (again, inverse Herfindahl index), the *absolute difference in the two diversity measures*, and the *number of categories* in class of all other non-binary attributes (see below for details). We exclude groups without any nominated friend and groups without outgroup members in the respective class. To check whether excluding groups without any nominated friend biases our results, we included these groups in supplementary analyses (with their ingroup share artificially assigned to either 0 or 1). However, as this sample restriction affects only six groups out of over 6,000, it does not meaningfully change the results (see S3 and S4 Tables in the Appendix). To ensure sufficient coverage of the classes’ network structure, we drop all groups from classes with less than 10 respondents, a participation rate below 60 percent, or 25 percent or more without any sociometric nominations. Moreover, to ensure comparability across the different attributes, we drop all groups from classes that are included only in some but not all seven groups-in-classes datasets. Across the seven resulting datasets (i.e., demographic attributes of interest), the final sample size varies between 800 and 1,348 groups in 524 school classes. See S5 and S6 Tables in the Appendix for descriptive information on the analytical samples.

Building on the approach of Kroneberg et al. [18], we examine the effects of attribute consolidation on *overall levels* of friendship segregation rather than focusing on individual preferences for similar others, as explored by Zhao [17]. Given our focus, network approaches such as exponential random graphs [39], which are designed to estimate segregation tendencies while accounting for other tie formation mechanisms, are not suited to our analysis. Instead, we regress the proportion of ingroup friends on the consolidation of the group-defining attribute with the other six attributes in 42 separate models using OLS regressions with cluster-robust standard errors. The 42 models are used to examine heterogeneity in consolidation effects across different combinations of attributes. Because Hypothesis 1 examines the significance of consolidation effects across multiple attribute combinations, we apply corrections for multiple testing [40]. In contrast, such corrections are unnecessary for Hypothesis 2, as it focuses on differences between estimates rather than the significance of a universally applying consolidation effect. The following equation presents our regression model, with attribute A and B defined by all possible combinations of the seven selected attributes in 42 separate models:$\begin{array}{cc} {IngroupShare}_{g,c,s}= & \;\beta_{\text{0}}+\beta_{\text{1}}{Consolidation}_{g,c,s}+\;\beta_{\text{2}}{ClassSize}_{c,s}+\beta_{\text{3}}{GroupSize}_{g,c,s}+\beta_{\text{4}}{DivA}_{g,c,s}\\ & +\;\beta_{\text{5}}{DivB}_{c,s}+\;\beta_{\text{6}}\left|{DivA}_{g,c,s}-{DivB}_{c,s}\right|+\;\beta_{\text{7}}{CategoriesB}_{c,s}+\;\gamma_{g,s}+\;\varepsilon_{g,c,s} \end{array}$

To address different sources of confounding, we include groups-in-survey-countries fixed effects $\gamma_{g,s}$ (i.e., dummy variables for the combination of group characteristics and survey countries, e.g., high-SES groups in Sweden or students of Turkish ethnic origin in Germany). Supplementary analyses using school fixed effects instead of groups-in-survey-country fixed effects yield similar results (see S7 Table in the Appendix). Further, we control for structural determinants of consolidation. We supplemented a set of determinants identified in prior research [18] with additional structural variables associated with consolidation in our sample, as revealed by exploratory analyses (see S8 Table in the Appendix). Specifically, we control for class size (25 in the example above), group size (10 for low-SES students in the example above), ingroup-outgroup diversity (measured as the inverse Herfindahl index of the proportions of in- and outgroup members, which equals 0.48 for the low-SES group in the example above), diversity in attribute B (also measured as the inverse Herfindahl index, which equals 0.5 for attribute B defined as gender in the example above), the absolute difference between the two diversity measures (0.02 in the example above), and the number of categories in attribute B (2 in the example above).

All analyses of Study 1 and 2 are conducted in R version 4.2.1 [41], using the packages *tidyverse, data.table, parallel, haven, labelled, mice, miceadds, rcompanion, igraph, netseg, caret, patchwork, writexl, MASS, scales, segregation, gridExtra,* and *randomForest*.

### Results

**Fig 2:**
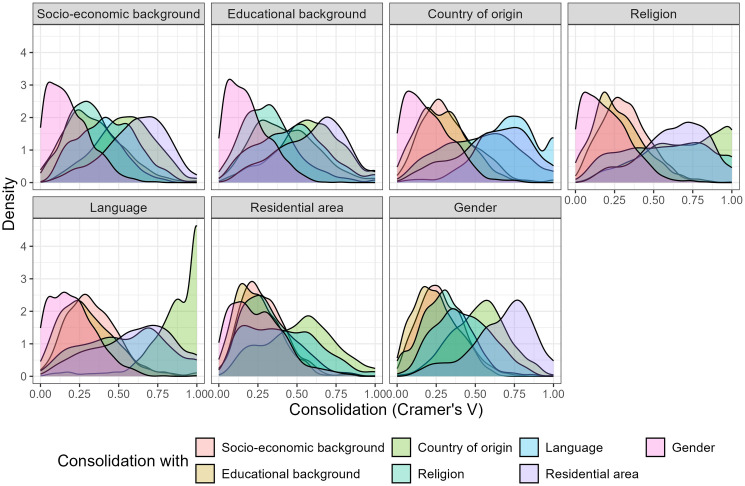
Attribute consolidation in 524 school classes in Germany, the Netherlands, and Sweden. The seven panels show the consolidation (Cramer’s V) of group membership as defined by seven demographic attributes (see panel headings) with the six other demographic attributes across all 524 classes in the analytical sample of Study 1.

The degree of consolidation potentially experienced by groups of demographically similar students varies across learning environments and attributes. Fig 2 illustrates how group membership, defined by the seven demographic attributes, aligns with six other attributes across the 524 school classes in the analytical sample. The distributions of these alignments vary considerably across various attribute combinations. Gender consolidation, which reflects the alignment between group membership and gender similarity, is right-skewed across all group-defining attributes. Most groups are placed in classes where the alignment between group membership and gender is small, but a few are situated in environments with strong gender consolidation. Conversely, the consolidation of language groups with countries of origin is strongly left-skewed, indicating that students who speak the same language at home typically share a similar country of origin. This contrast highlights the impact of cohort compositions in the production of attribute consolidation in school classes (see Study 2). While languages and countries of origin are often consolidated at broader levels (e.g., school cohorts or national populations), leaving little room for variation between classes, strong gender consolidation appears only in certain classes, likely as a random consequence of schools not prioritizing gender consolidation in student placements [18].

**Fig 3:**
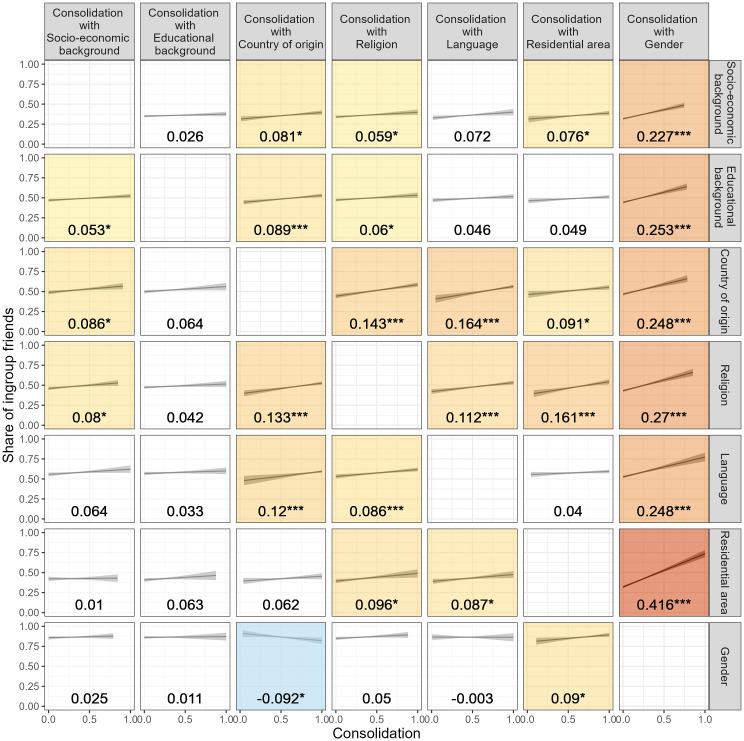
The association between attribute consolidation and friendship segregation across 42 attribute combinations. Conditional expected values plots and unstandardized regression coefficients of OLS regressions modelling the association between consolidation and the share of ingroup friends with cluster robust standard errors, groups-in-survey-countries fixed-effects, and controlled for class size, group size, ingroup-outgroup diversity, diversity of the consolidating attribute, the absolute difference in diversity, and the number of categories of the consolidating attribute. Pooled results over 10 imputed datasets using Rubin’s rules [38]. Background color of panels visualizes coefficient significance and size. ***p < 0.001 **p < 0.01 *p < 0.05.

Fig 3 illustrates the results of 42 regression models that estimate the relationship between the share of ingroup friends and attribute consolidation. The units of analysis are groups in classes (defined as at least three similar students). The seven rows in Fig 3 represent seven group-defining attributes. The seven columns represent the different consolidating attributes. The x-axis in each model quantifies the consolidation of group membership with the consolidating attribute, measured as Cramer’s V. Values of zero correspond to completely unconsolidated contexts and values of one correspond to perfectly consolidated contexts. The y-axis in each model shows the group’s share of ingroup friends. For each of the regression models, the figure shows conditional expected values plots and unstandardized regression coefficients with their significance levels. See S9 Table in the Appendix for full regression results.

Across models, positive relationships between attribute consolidation and friendship segregation are observed. Almost all coefficients are positive (40 of the 42 coefficients) and more than half of them are statistically significant and positive (25 coefficients). Even after applying a Bonferroni correction that divides the significance threshold by 42 – a highly conservative adjustment given the large number of tests – 14 coefficients remain statistically significant (see S10 Table in the Appendix). This indicates that the observed consolidation effects are unlikely to be mere artifacts of multiple testing. Consistent with theoretical expectations, consolidation is positively related to friendship segregation across a wide range of demographic attributes.

Moreover, there is considerable variation in the strength of this relationship across the seven consolidating attributes. Gender consolidation stands out with particularly strong associations with friendship segregation (see last column in Fig 3). Here, the coefficients vary between 0.227 and 0.416, are all statistically significant (t-values between 7 and 14.48, see S9 Table in the Appendix), and are almost all significantly higher than the other attributes’ consolidation coefficients (see S11 Table in the Appendix). As expected, among all demographic attributes under investigation, gender consolidation is particularly important for friendship segregation.

Further, friendship segregation by gender is unrelated to most types of attribute consolidation (see last row in Fig 3). The coefficient sizes for the relationship between friendship segregation by gender and the consolidation with socio-economic background (0.025, t = 0.72), educational background (0.011, t = 0.32), religion (0.05, t = 1.54), and language (−0.003, t = −0.06) are close to zero and insignificant, the consolidation with country of origin even negative (−0.092, t = −2.1), and only the consolidation with residential area significantly positive related to gender segregation (0.09, t = 2.27). Gender segregation in adolescents’ friendships is prevalent across contexts, largely independent of the extent of consolidation with other demographic attributes.

Finally, Fig 3 reveals remarkably high coefficient sizes for the combination of the three ethno-religious attributes (country of origin, religion, and language) and the combination of residential area with religion. For example, while language consolidation is not or only weakly related to segregation along the lines of socio-economic background (0.072, t = 1.95), educational background (0.046, t = 1.62), residential area (0.087, t = 2.28), and gender (−0.003, t = −0.06), its relation to country-of-origin segregation is surprisingly high (0.164, t = 4.48). This suggests the presence of salience effects that increase friendship segregation beyond the mechanical effects of attribute consolidation. Preferences to form outgroup friendships may increase in distinctive ways when certain attributes are consolidated due to unique experiences or identities that lie at the intersections of attributes. However, despite the potential presence of other segregation-enhancing mechanisms for certain attribute combinations, none of these coefficients reach the overarching strong consolidation effects imposed by gender.

## Study 2. Exploring the effectiveness of class placement in reducing friendship segregation

Adopting a solution-oriented stance [42], we are not only interested in the relative strength of different consolidation effects in school classes, but also in their potential to reduce friendship segregation among adolescents. To evaluate this potential, we compare observed levels of friendship segregation to a counterfactual scenario in which class placements are designed to minimize attribute consolidation. While there is little systematic knowledge about the principles guiding actual placement practices, it is plausible that some student attributes—most notably gender—receive greater attention than others. In the absence of such information, it remains uncertain whether a hypothetical strategy explicitly aimed at minimizing attribute consolidation would, in fact, yield meaningful reductions in consolidation and, consequently, in friendship segregation relative to current practices (which may for example often aim for gender balance across classes but potentially not minimal levels of gender consolidation).

**Fig 4:**
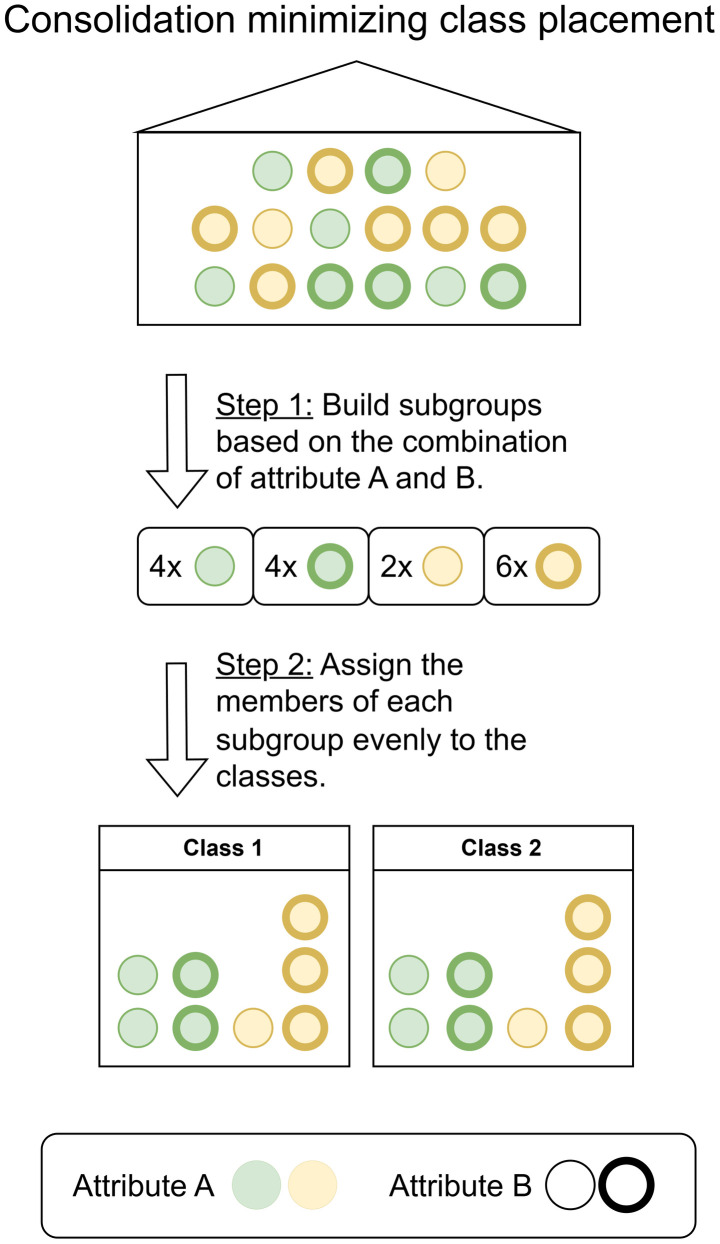
A sorting heuristic that minimizes consolidation in class placement. Schools can minimize the consolidation of two attributes across school classes by, first, creating subgroups based on the combination of both attributes and, second, assigning the members of each subgroup evenly to classes.

As shown in Fig 4, attribute consolidation can be minimized across classes in a cohort by following a simple sorting heuristic: First, build subgroups based on the combination of the two selected attributes A and B. Second, assign the members of each subgroup evenly to the classes. While this sorting heuristic can serve as a benchmark for evaluating the effectiveness of accounting for attribute consolidation, it is only hypothetical, given that school administrators face various constraints and requirements in their placement decisions. Nevertheless, this sorting method not only results in minimal levels of attribute consolidation across classes but also maximizes diversity along the two attributes simultaneously. For example, by minimizing the consolidation of gender and religion, school administrators produce classes that exhibit not only minimal consolidation but also balanced representations of the different genders and religions.

Importantly, the effectiveness of any form of class placement, including the outlined hypothetical sorting heuristic, will to a large degree depend on the *sociodemographic composition of the school cohort* to be sorted. A classical structuralist argument suggests that “[t]he degree of differentiation in a social structure is always greater than the average degree of differentiation in its substructures” [21: 155]. This means that the average consolidation across school classes is always at least as high as the consolidation at the higher level of the whole cohort of students in a given school or school system. To illustrate, if the students’ religions are already perfectly consolidated with their genders in the overall student cohort, they will also be perfectly consolidated within each of the school classes. And similar arguments may apply concerning the impact of other compositional characteristics of a cohort. For example, in addition to the consolidation of students’ religion and gender in a cohort, the degree of diversity in one of the two attributes alone may also have an impact on the effectiveness of accounting for consolidation. In general, the composition of the whole cohort of students determines the scope for school administrators to achieve different levels of attribute consolidation through their sorting decision.

In light of the many functional dependencies between compositional characteristics, it is difficult to derive explicit expectations about how specific compositions of schools influence consolidation effects. Instead, we explore this variation inductively, based on class placement simulations across the empirically observable value space of cohort compositions worldwide.

### Data and methods

We aim to determine how much friendship segregation in schools could be reduced by considering attribute consolidation in class placement. Based on the findings of Study 1, we test the proposed sorting heuristic (see Fig 4) with a focus on gender, as *minimizing gender consolidation* appears to have the strongest impact on reducing segregation (see Study 1). Our analysis consists of two sets of simulations. First, we compare the heuristic’s outcomes to actual class placements in schools in Germany, the Netherlands, and Sweden. Second, we move beyond real-world placements to explore whether the heuristic’s effectiveness depends on the demographic composition of the student body. By applying the heuristic to a range of empirically plausible cohort compositions, we assess how consistently it reduces friendship segregation across different student populations.

For the first set of simulations, we again use data from the first wave of the “Children of Immigrants Longitudinal Survey in Four European Countries (CILS4EU)” [35]. We restrict the sample used in Study 1 to 188 schools for which we have data on two randomly selected classes. We use the same selection and measurement of attributes as in Study 1. Only residential areas are excluded from Study 2, because information on students’ residential proximity is only available within classes and not across them. For each of the attributes socio-economic background, educational background, country of origin, religion, and language, we simulate class placements that *minimize the consolidation of the selected attribute and gender*: We create subgroups based on the combination of the respective attribute and gender, and then assign the subgroup members evenly to two classes (see Fig 4). This sorting heuristic minimizes the consolidation of the selected attribute with gender while maximizing the diversity of gender and the selected attribute across classes. We simulate this sorting heuristic 200 times for each of the five selected attributes and 188 schools in the sample. As a benchmark for comparison, we use the *observed class placements*. In supplementary analyses (S1 Fig in the Appendix), we replace this benchmark with 200 simulations of *random class placements*.

For each of the observed and simulated classes we predict friendship segregation based on the models of Study 1. First, we transform the data analogously to Study 1 into a groups-in-classes structure and generate a separate dataset for each demographic attribute. Second, we generate the same variables as in Study 1: The consolidation of group membership with gender, class size, group size, ingroup-outgroup diversity, gender diversity, and the absolute difference in the two diversity measures. Third, we use the models estimated in Study 1 (M37 to M41 in S9 Table in the Appendix) to predict the share of ingroup friends for the groups in the observed and simulated classes. As the outcome measure of each class placement, we report the maximum predicted ingroup share across all groups in the two classes. That is, we evaluate the performance of a class placement based on the predicted segregation for the most segregated group. Rather than minimizing segregation in one group or class, possibly at the expense of increasing segregation in a less advantaged group or class, this outcome focuses on the potential payoff for the ‘least advantaged’ group in a school (though possibly at the expense of a more advantaged group [43]). In S1 Text in the Appendix, we describe the rationale and implications of the selected outcome measure in more detail. Finally, we compute the difference in this outcome measure between the *observed class placements* and the averages over 200 simulations of our hypothetical sorting heuristic that *minimizes gender consolidation*.

For the second set of simulations, we rely on unweighted data from the “OECD Programme for International Student Assessment (PISA)” in 2018. The PISA 2018 database comprises survey responses of 612,004 students in 21,903 schools in 80 countries. In each country, students were sampled in a two-stage stratified sampling design: In stage 1, 150 schools were randomly selected within each country with probabilities proportional to the number of 15-year-old students. In stage 2, 42 students at the age of 15 were randomly selected within each school. In 809 schools in 20 countries, the number of respondents exceeds 42 due to these countries’ participation in an optional module [44]. We draw a random sample of 42 students from these schools to avoid biases in the school comparisons. In supplementary analyses with the full cohorts of these 809 schools, we test how this affects the results of Study 2 (see S12 Table in the Appendix). All procedures for data collection and consent were managed by national project teams in accordance with OECD guidelines and respective national regulations. PISA data is publicly available through the OECD database and is de-identified to ensure participant confidentiality. Ethical approval for the presented study was not required, as the analysis is based on anonymized secondary data that does not involve direct interaction with human subjects.

Students’ demographic attributes are defined similarly to the analyses relying on CILS4EU data (see S2 Text in the Appendix for a detailed comparison of coding schemes). However, given the lack of information on students’ religion and residential area, we limit our analysis to the remaining five demographic attributes. We drop all schools in Singapore due to missing language information and all schools with less than 20 respondents (i.e., the minimal class size is 10 students, as in Study 1), resulting in a final sample size of 521,210 students in 16,117 schools in 79 countries. We impute missing values in all five variables ten times using multivariate imputation by chained equations, repeat the following steps for each imputed dataset and combine the results using Rubin’s rules [38].

For each of the 16,117 schools, we simulate 200 class placements that *minimize gender consolidation* (for each of the attributes socio-economic background, educational background, country of origin, and language spoken at home) and 200 *random class placements*. Analogously to the first simulation study, we then transform the data into a groups-in-class structure, predict for each group the share of ingroup friends using the models estimated in Study 1, and summarize the predictions by selecting the maximum predicted ingroup share across all groups and classes within each simulated class placement. Finally, we compute the difference in this outcome measure between the averages over 200 simulations of our suggested sorting heuristic that *minimizes gender consolidation* and 200 *random class placements*. See S1 Text and S2 Fig in the Appendix for a more detailed description and visualization of the simulation. In supplementary analyses, we use *gender balanced class placements* (i.e., assigning boys and girls evenly to the two classes) instead of random class placements as the benchmark of comparison (see S13 Table and S4 Fig in the Appendix).

In a second step, we then analyze how the estimated potential impact of the sorting heuristic that *minimizes gender consolidation* is related to distributional characteristics at school and country level. Using random forests with 5-fold cross-validation, we explore how well the simulated reductions in friendship segregation are predicted by five compositional characteristics of schools: The diversity in the group-defining attribute (measured as the inverse Herfindahl index), the consolidation of the selected attribute and gender (measured as Cramer’s V), the number of students, the gender diversity (measured as the inverse Herfindahl index), and the number of categories in the group-defining attribute (see S13 Table in the Appendix for the summary statistics of all included variables). To assess the importance of these five school characteristics, we measure each variable’s contribution to the model’s prediction accuracy. We do this by comparing how much the models’ accuracy in predicting the outcome decreases after randomly permuting the values of each variable one at a time. Finally, we explore the functional shape of each variable’s marginal effects on the outcome variable with partial dependence plots.

### Results

**Fig 5:**
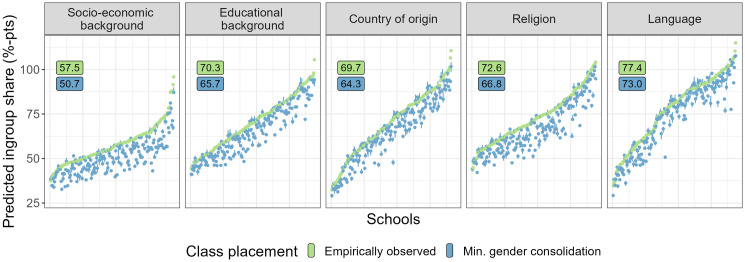
Difference in predicted friendship segregation between observed and simulated class placements. Share of ingroup friends predicted with the models of Study 1 for 188 empirically observed (green) and simulated counterfactual (blue) class placements in Germany, the Netherlands, and Sweden.

In the first set of simulations, we examine how friendship segregation, as predicted by the models estimated in Study 1, differs between empirically observed class placements in 188 German, Dutch, and Swedish schools and simulations of counterfactual class placements that minimize gender consolidation. Fig 5 visualizes the results. The green points in each panel show the highest predicted share of ingroup friends for groups in the observed classes. The blue points show for the same schools the highest predicted share of ingroup friends averaged over 200 simulations of gender consolidation minimizing class placements (interquartile ranges are visualized by the blue lines around each point). The average predicted share of ingroup friends across the 188 schools decreases from 57.5 to 50.7 percent for socio-economic background groups, from 70.3 to 65.7 percent for educational background groups, from 69.7 to 64.3 percent for country of origin groups, from 72.6 to 66.8 percent for religion groups, and from 77.4 to 73 percent for language groups. This corresponds to an average estimated decrease of 4–7 percentage points.

Fig 5 reveals a considerable between-school variation in the estimated reductions of friendship segregation (Std. Dev. = 5.5, 4.6, 4.8, 5.6, and 5.1 percentage points). While the predicted reduction in students’ share of ingroup friends is close to zero for some schools, it ranges up to 26 percentage points in the most extreme case.

This variation could stem from two sources: differences in current sorting approaches (e.g., whether school administrators balance ethnicity across classes) and differences in cohort compositions (e.g., the level of ethnic diversity within a school). To disentangle the roles of these factors, we conduct supplementary analyses that use random class placements – rather than the empirically observed class placements – as the benchmark of comparison (see S1 Fig in the Appendix). In this analysis, school differences in the potential impact of the sorting heuristic can only be attributed to compositional differences, not to observed sorting approaches. Compared to random class placements, the suggested sorting heuristic could reduce friendship segregation on average by 3–6 percentage points, with standard deviations between 1 and 2 percentage points and a maximum predicted reduction of 10 percentage points. Even when differences in sorting approaches are held constant, the heuristic’s potential effectiveness still varies between schools. This indicates that the cohort’s composition plays an important role in determining how effective the proposed sorting heuristic can be.

To examine how compositional features of the cohort to be sorted shape the potential effects of the proposed sorting heuristic, we run another set of simulations using a much larger dataset. To address the complete, plausible value space of cohort compositions, we simulate the proposed sorting heuristic for 16,117 empirically observed schools in 79 countries. To focus on the role of cohort compositions in shaping the potential effects of the hypothetical sorting heuristic, we use random class placements as the benchmark of comparison. A comparison with gender balanced class placements yields similar results (see S13 Table and S4 Fig in the Appendix). We do this separately for the available attributes of socio-economic background, educational background, country of origin, and language. The extent to which the hypothetical sorting heuristic could reduce friendship segregation varies considerably across schools and attributes. In 94% of the simulated cases, the reductions are positive, up to a maximum reduction of 11 percentage points. On average, the simulated reductions in ingroup shares with respect to students’ socio-economic backgrounds, educational backgrounds, countries of origin, and languages spoken at home are 4.8, 4.6, 2.4, and 2.3 percentage points (Std. Dev. = 1.6, 1.9, 1.9, and 2.1 percentage points).

**Fig 6:**
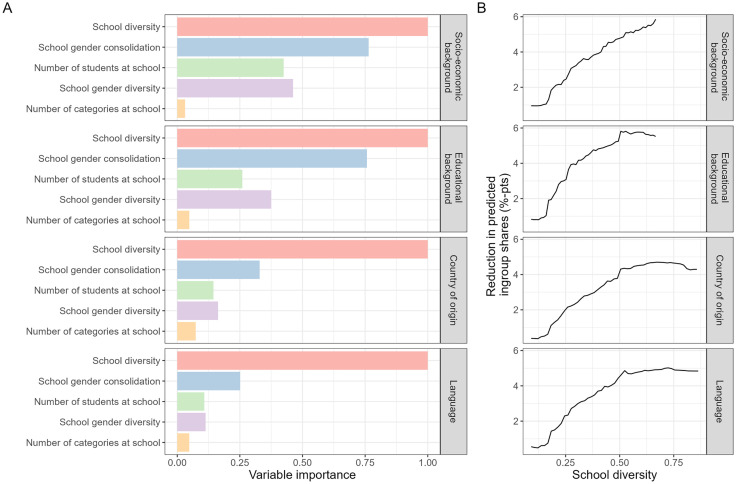
Random forest analysis of factors predicting simulated reductions in friendship segregation. Results of random forests that predict simulated reductions in friendship segregation with gender consolidation minimizing class placements (compared to random class placements) for 16,117 schools in 79 countries with 5-fold cross-validation. **(A)** Relative importance of each predictor variable based on a comparison of prediction errors after permuting each of the variables in the out-of-bag data. **(B)** Partial dependence plots with the marginal effects of school diversity on reductions in predicted ingroup shares.

Fig 6 summarizes the results of exploratory analyses using random forests. School diversity is the most important of the compositional variables in predicting the potential effectiveness of the hypothetical sorting heuristic. Panel B in Fig 6 shows the marginal effects of school diversity on the reductions in predicted ingroup shares. The relationship is positive with decreasing steepness, meaning that the proposed sorting heuristic can be more effective in more diverse schools. S5 Fig in the Appendix shows the marginal effects of the other compositional variables. The sorting heuristic’s potential effectiveness is larger when the cohort to be sorted is characterized by a low consolidation of the selected attribute and gender, higher number of students, and a stronger gender diversity.

In supplementary analyses, we explore the potential implications of the observed positive relationship between school diversity and the sorting heuristic’s effectiveness for schools in national contexts with different compositional features. S6 Fig and S14 Table in the Appendix indicate that the proposed sorting heuristic could theoretically be more effective in countries with high levels of diversity and low levels of between-school segregation. It is important to note, however, that this analysis does not account for country-specific differences in sorting practices or in the effects of gender consolidation. Instead, it relies solely on differences in school compositions and should therefore be interpreted with caution.

Overall, schools vary considerably in the extent to which the sorting heuristic that *minimizes gender consolidation* can reduce friendship segregation. While much of this variation stems from differences in observed class placements, the composition of the student cohort also plays a role. In particular, diverse schools – which are more common in countries with high levels of diversity and low levels of between-school segregation – may provide more favorable structural conditions for diverse friendships by trying to reduce gender consolidation in their class placement.

## Discussion

The persistence of friendship segregation among adolescents highlights that placing students in diverse schools is not sufficient to promote friendship diversity. In the search for an effective strategy for schools to promote friendship diversity, this research has uncovered the potential of minimizing attribute consolidation in class placement. Based on two comprehensive studies this article offers novel insights into how within-school sorting decisions shape friendship networks among adolescents in secondary schools. It does not only enhance our knowledge of consolidation effects on friendship segregation, but yields insights of practical relevance to school administrators who apply class placement in their school.

Study 1 found that the consolidation of attributes in school classes is positively related to friendship segregation among adolescents. This relationship was observed across various demographic attributes, with the strongest effects seen when gender was the consolidating attribute. While previous research highlighted the importance of SES consolidation for *net* ethnic friendship segregation [17], our findings show that, for the *gross* segregation observed in friendship networks, salience effects like these are far outweighed by the strong mechanical effect of gender consolidation. This finding underscores the role of gender as a central axis along which adolescents’ social networks are organized. Gender consolidation in school classes, i.e., the alignment of relevant demographic attributes with gender, can significantly affect the likelihood of adolescents forming diverse friendships.

Study 2 extended these findings by examining the effectiveness of a sorting heuristic that reduces gender consolidation within school classes. The simulation approach used in Study 2 offered a key strength: it enabled counterfactual estimation of how much friendship segregation could be reduced if school administrators minimized attribute consolidation in their class placement decisions. By combining the consolidation effects estimated in Study 1 with realistic cohort compositions and class capacities, the simulation revealed the compositional conditions under which such placement strategies would actually matter. This allowed for an assessment of the potential desegregating impact of reducing consolidation vis-à-vis actual class placements and cohort compositions currently faced—something that cannot be inferred from consolidation effects alone. However, the approach also had clear limitations. It did not account for the multiple, often competing aims that guide real-world class placements. As such, it remained agnostic about whether minimizing consolidation is a feasible policy objective, offering only an estimate of the maximum possible reduction in friendship segregation that could be achieved under idealized placement conditions. Recognizing that, in reality, school administrators face a variety of factors to consider in their placement decisions that are not accounted for in the sorting heuristic, this analysis indicated that the proposed heuristic can create favorable structural conditions for fostering more integrated social networks in educational settings. Simulations showed that implementing this heuristic could substantially reduce friendship segregation, especially in diverse schools located mostly in diverse countries with low levels of between-school segregation.

The main findings of the two studies are supported and extended by several supplementary analyses. In support of the proposed sorting heuristic, it should be noted that additional analyses show no evidence of unintended consequences for student achievement, loneliness, or school satisfaction (see S15 and S16 Tables in the Appendix). Further, its impact on reducing segregation is likely to be underestimated (by 27–89 percent) due to the rather small cohort sizes following the PISA sampling design (see S12 Table in the Appendix) and the lack of information on observed class placements in PISA 2018.

Still, future research will need to address two analytic challenges. First, class placements with different levels of gender consolidation are likely to be largely conditionally ignorable [18], suggesting valid causal inference. However, other attribute combinations may be at a greater risk of confounding. In Study 1, we included groups-in-survey-countries fixed effects to control for group-level confounding. Yet, some of the observed positive associations may have been driven by other confounding variables (e.g., neighborhood composition). Future research should investigate the extent to which the positive relationships between attribute consolidation and friendship segregation in school classes can be interpreted as causal. Second, while gender consolidation stands out with particularly strong associations with friendship segregation in the three European countries under study, little is known about its importance in other educational systems. A systematic literature review demonstrates a remarkable consistency of gender segregation in adolescent friendships across countries (see S1 Table in the Appendix), indicating that gender homophily in friendships is a global phenomenon. This supports the idea that reducing gender consolidation in class placement can help decrease friendship segregation in school. However, applying our findings in diverse international contexts requires careful consideration of each country’s unique socio-cultural dynamics, as well as the varying goals and strategies school administrators use when assigning students to classes.

Our research provides empirical support for consolidation effects on segregation – a classical structuralist argument in sociology, political science, and management science that has been largely overlooked in practical school settings. To our knowledge, neither country policies for class placement, nor the most popular commercial tools for helping teachers with their sorting decisions take gender consolidation into account. This gap highlights a significant opportunity. Schools can actively work to reduce segregation in adolescent friendships by considering the consolidation of relevant demographic attributes with gender in their class placement, thereby fostering more inclusive and diverse social environments.

## Supporting information

S1 Fig Simulations with random class placements as the comparison baseline. Share of ingroup friends predicted with the models of Study 1 for groups in 200 *randomly* sorted classes and 200 classes with *minimized gender consolidation*, simulated for 188 observed student cohorts in Germany, the Netherlands, and Sweden.
(PNG)

S2 Fig Visualization of the simulation procedure for school 78400188. (A) The distribution of gender groups and gender-attribute subgroups in 2 of the 200 simulations of gender balanced class placements (i.e., class placement that maximizes gender diversity across classes) and class placements that minimize the consolidation of the selected attribute and gender. UAE = United Arab Emirates; Other GCC = Another State of the Gulf Cooperation Council. (B) Average gender diversity and gender consolidation for each group and class (in red for the class with the maximum value and in blue for the class with the minimum value, respectively). (C) Distributions of predicted ingroup shares for the three types of sorting strategies for each attribute. Red points show the averages of each distribution and red lines and numbers the difference to the average predicted ingroup share for gender consolidation minimizing class placements.
(PNG)

S3 Fig Number of countries of origin and languages in schools in the 79 countries included in Study 2’s main analyses. Red asterisks show the overall number of categories in the respective survey country, boxplots show the distribution of category numbers within schools. ALB = Albania, ARE = United Arab Emirates, ARG = Argentina, AUS = Australia, AUT = Austria, BEL = Belgium, BGR = Bulgaria, BIH = Bosnia Herzegovina, BLR = Belarus, BRA = Brazil, BRN = Brunei, CAN = Canada, CHE = Switzerland, CHL = Chile, COL = Colombia, CRI = Costa Rica, CZE = Czech Republic, DEU = Germany, DNK = Denmark, DOM = Dominican Republic, ESP = Spain, EST = Estonia, FIN = Finland, FRA = France, GBR = United Kingdom, GEO = Georgia, GRC = Greece, HKG = Hong Kong, HRV = Croatia, HUN = Hungary, IDN = Indonesia, IRL = Ireland, ISL = Iceland, ISR = Israel, ITA = Italy, JOR = Jordan, JPN = Japan, KAZ = Kazakhstan, KOR = Korea, KSV = Kosovo, LBN = Lebanon, LTU = Lithuania, LUX = Luxembourg, LVA = Latvia, MAC = Macao, MAR = Morocco, MDA = Moldova, MEX = Mexico, MKD = North Macedonia, MLT = Malta, MNE = Montenegro, MYS = Malaysia, NLD = Netherlands, NOR = Norway, NZL = New Zealand, PAN = Panama, PER = Peru, PHL = Philippines, POL = Poland, PRT = Portugal, QAT = Qatar, QAZ = Baku Azerbaijan, QCI = B-S-J-Z China, QMR = Moscow Region Russian Federation, QRT = Tatarstan Russian Federation, ROU = Romania, RUS = Russian Federation, SAU = Saudi Arabia, SRB = Serbia, SVK = Slovak Republic, SVN = Slovenia, SWE = Sweden, TAP = Chinese Taipei, THA = Thailand, TUR = Turkey, UKR = Ukraine, URY = Uruguay, USA = United States of America, VNM = Vietnam.
(PNG)

S4 Fig Random forest results for simulations with gender balanced class placements as the comparison baseline. Results of random forests that predict simulated reductions in friendship segregation with gender consolidation minimizing class placements (compared to *gender balanced class placements*) for 16,117 schools in 79 countries with 5-fold cross-validation. (A) Relative importance of each predictor variable based on a comparison of prediction errors after permuting each of the variables in the out-of-bag data. (B) Partial dependence plots with the marginal effects of school diversity on reductions in predicted ingroup shares.
(PNG)

S5 Fig Partial dependence plots with the marginal effects of the five included predictor variables on reductions in predicted ingroup shares. Results of random forests that predict simulated reductions in friendship segregation with gender consolidation minimizing class placements (compared to random class placements) for 16,117 schools in 79 countries with 5-fold cross-validation.
(PNG)

S6 Fig Relationships between school- and country-level characteristics and the predicted reductions in friendship segregation with gender consolidation minimizing instead of random class placements for 16,117 secondary schools in 79 countries. (A) Linear relationship of school diversity (measured as inverse Herfindahl index) and simulated reductions in friendship segregation. (B) Linear relationship between country diversity (measured as inverse Herfindahl index) and the country’s average simulated reduction in friendship segregation for countries with low (green) and high (orange) between-school segregation (Theil index below or above median).
(PNG)

S1 Text Simulated class placement. (DOCX)

S2 Text Coding schemes in Studies 1 and 2. (DOCX)

S1 Table Gender segregation in adolescents’ school friendships around the world. (DOCX)

S2 Table OLS models regressing the share of ingroup friends on consolidation using only complete cases. (DOCX)

S3 Table OLS models regressing the share of ingroup friends on consolidation, including six groups without any friendship nominations (setting their ingroup share to 0). (DOCX)

S4 Table OLS models regressing the share of ingroup friends on consolidation, including six groups without any friendship nominations (setting their ingroup share to 1). (DOCX)

S5 Table Missingness and number of categories in the seven group-defining attributes. (DOCX)

S6 Table Summary statistics of the analytical samples for the seven group-defining attributes. (DOCX)

S7 Table OLS models regressing the share of ingroup friends on consolidation with school fixed effects. (DOCX)

S8 Table OLS models regressing consolidation on structural variables. (DOCX)

S9 Table OLS models regressing the share of ingroup friends on consolidation. (DOCX)

S10 Table Significance of consolidation estimates with Bonferroni correction. (DOCX)

S11 Table Difference between the coefficients of gender consolidation and the other types of consolidation. (DOCX)

S12 Table Simulation results for 809 schools with full cohort data. Summary statistics of predicted reductions in ingroup shares (in %-pts.) with class placements that minimize gender consolidation compared to gender balanced/ random class placements in 809 schools with full cohort data.
(DOCX)

S13 Table Summary statistics of the second set of simulations based on PISA data underlying the random forests. (DOCX)

S14 Table OLS models regressing the (average) simulated reduction in friendship segregation on school and country level predictors based on PISA data. (DOCX)

S15 Table OLS models regressing the number of friends in class, math performance and school satisfaction on consolidation (including quadratic terms). (DOCX)

S16 Table OLS models regressing the number of friends in class, math performance and school satisfaction on consolidation (including quadratic terms) for small groups with up to four members. (DOCX)

S17 Table Missingness and number of categories in the four group-defining attributes in PISA. (DOCX)

S18 Table All survey questions used for the analyses. (DOCX)
