# Supplementary figures and images for: Friendship segregation and class composition in schools: A systematic analysis of the role of attribute consolidation
Source: PLoS One. 2025 Dec 31;20(12):e0339581. doi: 10.1371/journal.pone.0339581 (PMC12755804; doi:10.1371/journal.pone.0339581)

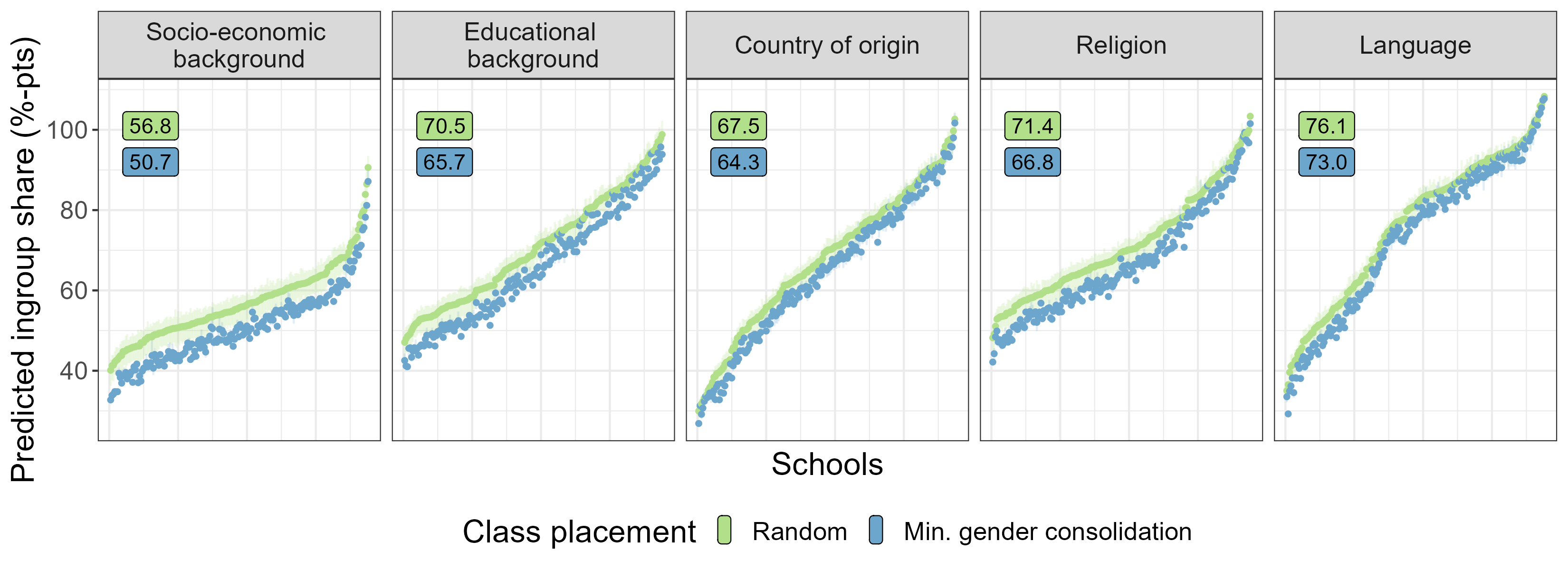

Supplement: S1 Fig — Share of ingroup friends predicted with the models of Study 1 for groups in 200 randomly sorted classes and 200 classes with minimized gender consolidation, simulated for 188 observed student cohorts in Germany, the Netherlands, and Sweden. (PNG) [file pone.0339581.s001.png]

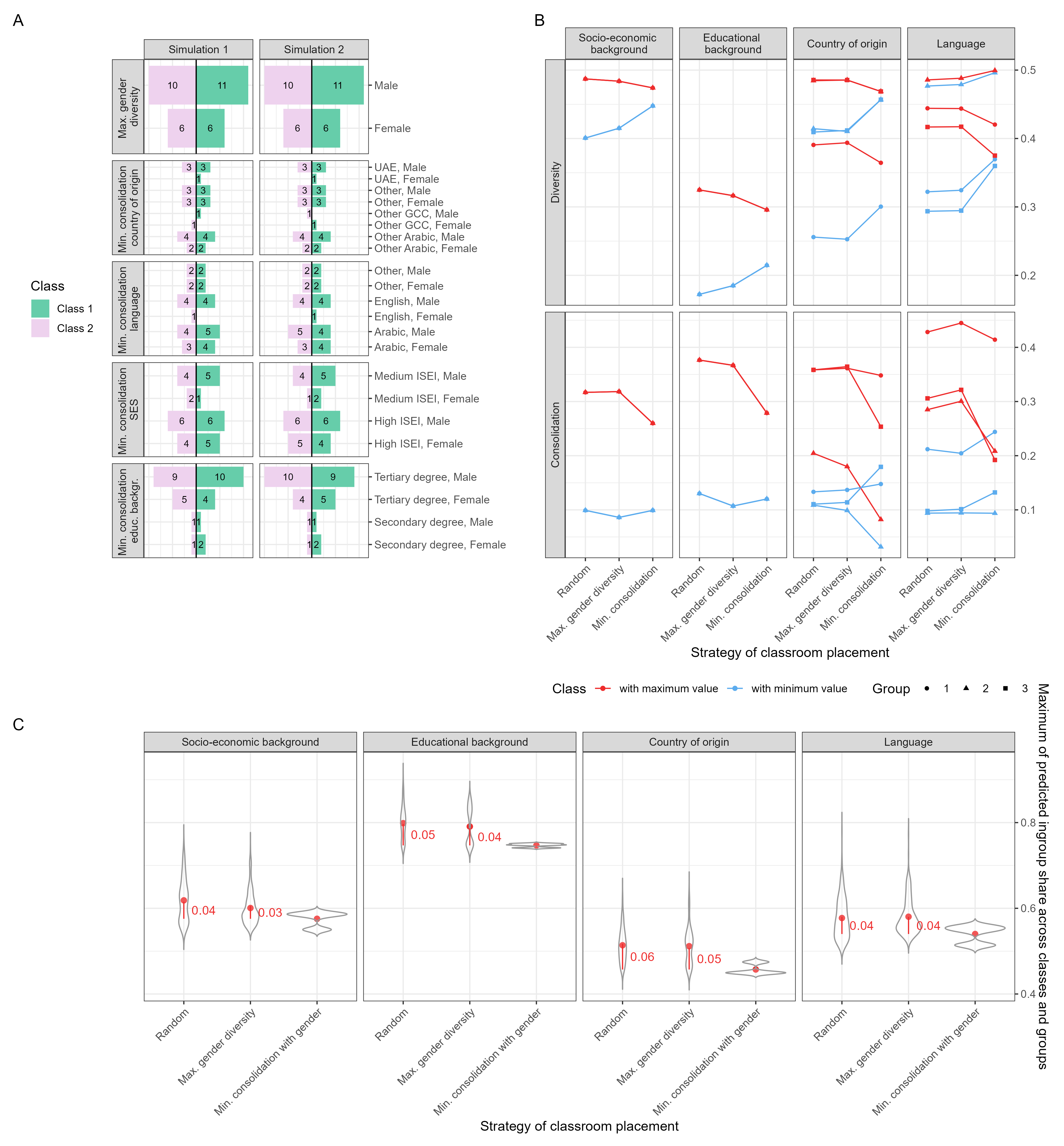

Supplement: S2 Fig — (A) The distribution of gender groups and gender-attribute subgroups in 2 of the 200 simulations of gender balanced class placements (i.e., class placement that maximizes gender diversity across classes) and class placements that minimize the consolidation of the selected attribute and gender. UAE = United Arab Emirates; Other GCC = Another State of the Gulf Cooperation Council. (B) Average gender diversity and gender consolidation for each group and class (in red for the class with the maximum value and in blue for the class with the minimum value, respectively). (C) Distributions of predicted ingroup shares for the three types of sorting strategies for each attribute. Red points show the averages of each distribution and red lines and numbers the difference to the average predicted ingroup share for gender consolidation minimizing class placements. (PNG) [file pone.0339581.s002.png]

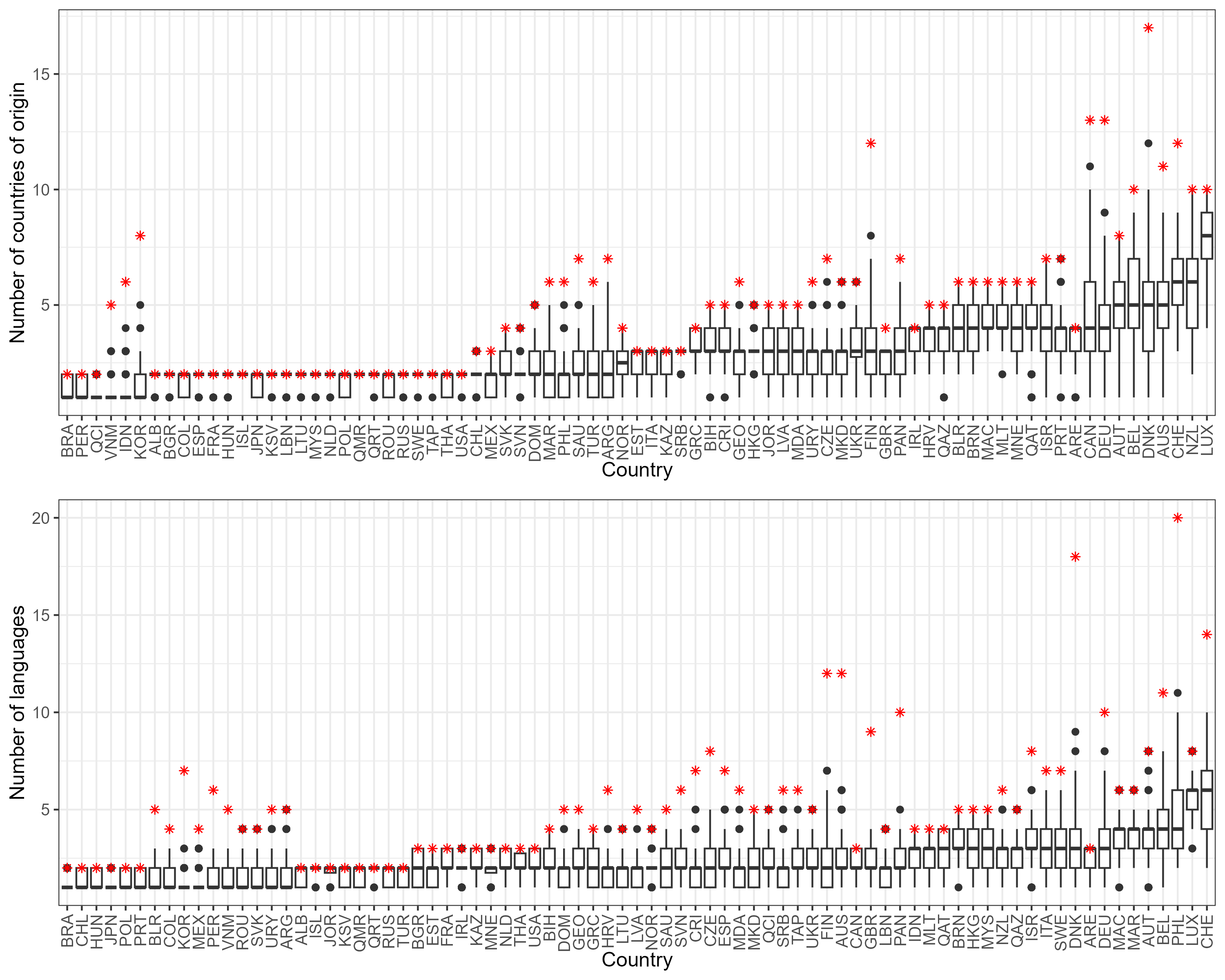

Supplement: S3 Fig — Red asterisks show the overall number of categories in the respective survey country, boxplots show the distribution of category numbers within schools. ALB = Albania, ARE = United Arab Emirates, ARG = Argentina, AUS = Australia, AUT = Austria, BEL = Belgium, BGR = Bulgaria, BIH = Bosnia Herzegovina, BLR = Belarus, BRA = Brazil, BRN = Brunei, CAN = Canada, CHE = Switzerland, CHL = Chile, COL = Colombia, CRI = Costa Rica, CZE = Czech Republic, DEU = Germany, DNK = Denmark, DOM = Dominican Republic, ESP = Spain, EST = Estonia, FIN = Finland, FRA = France, GBR = United Kingdom, GEO = Georgia, GRC = Greece, HKG = Hong Kong, HRV = Croatia, HUN = Hungary, IDN = Indonesia, IRL = Ireland, ISL = Iceland, ISR = Israel, ITA = Italy, JOR = Jordan, JPN = Japan, KAZ = Kazakhstan, KOR = Korea, KSV = Kosovo, LBN = Lebanon, LTU = Lithuania, LUX = Luxembourg, LVA = Latvia, MAC = Macao, MAR = Morocco, MDA = Moldova, MEX = Mexico, MKD = North Macedonia, MLT = Malta, MNE = Montenegro, MYS = Malaysia, NLD = Netherlands, NOR = Norway, NZL = New Zealand, PAN = Panama, PER = Peru, PHL = Philippines, POL = Poland, PRT = Portugal, QAT = Qatar, QAZ = Baku Azerbaijan, QCI = B-S-J-Z China, QMR = Moscow Region Russian Federation, QRT = Tatarstan Russian Federation, ROU = Romania, RUS = Russian Federation, SAU = Saudi Arabia, SRB = Serbia, SVK = Slovak Republic, SVN = Slovenia, SWE = Sweden, TAP = Chinese Taipei, THA = Thailand, TUR = Turkey, UKR = Ukraine, URY = Uruguay, USA = United States of America, VNM = Vietnam. (PNG) [file pone.0339581.s003.png]

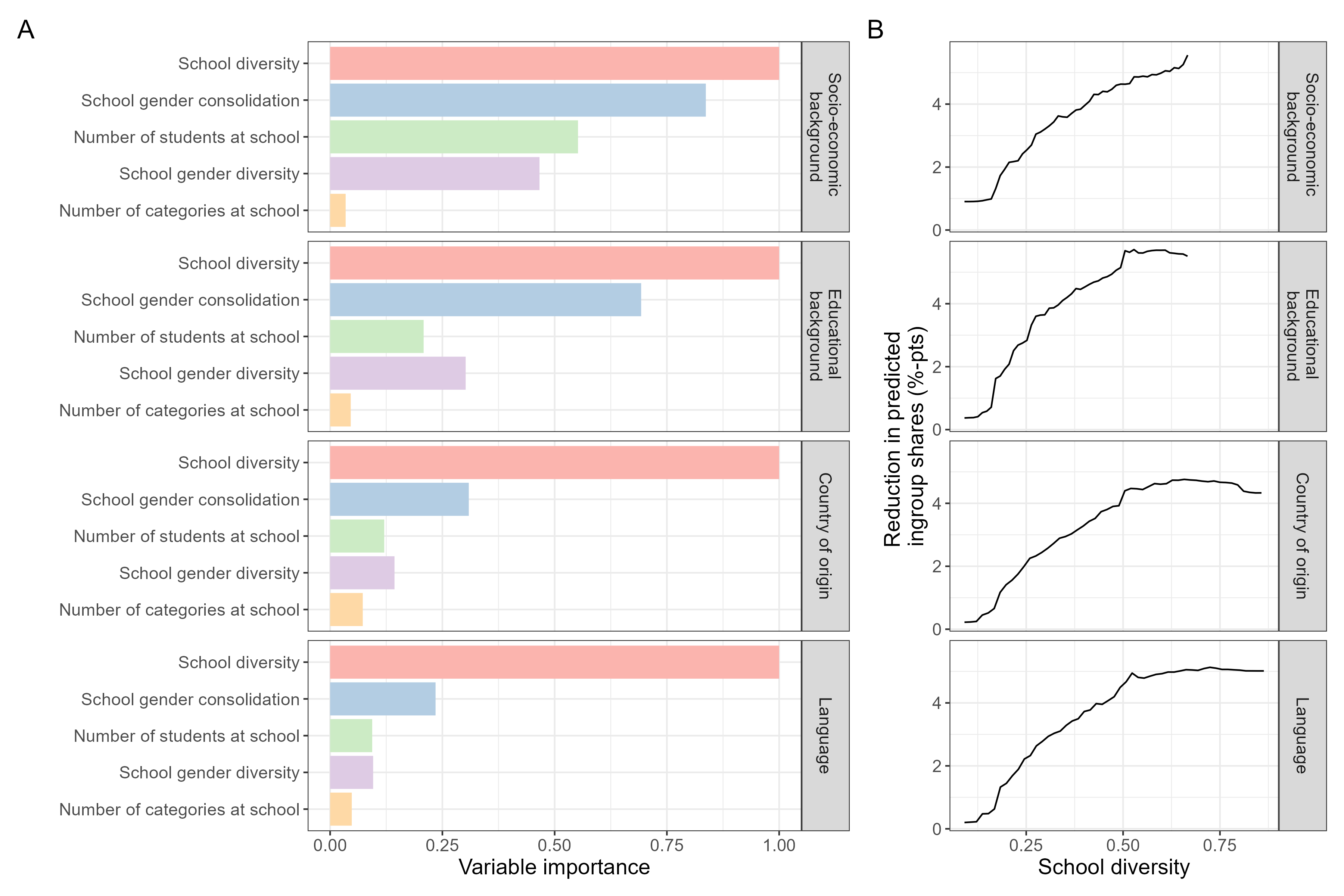

Supplement: S4 Fig — Results of random forests that predict simulated reductions in friendship segregation with gender consolidation minimizing class placements (compared to gender balanced class placements) for 16,117 schools in 79 countries with 5-fold cross-validation. (A) Relative importance of each predictor variable based on a comparison of prediction errors after permuting each of the variables in the out-of-bag data. (B) Partial dependence plots with the marginal effects of school diversity on reductions in predicted ingroup shares. (PNG) [file pone.0339581.s004.png]

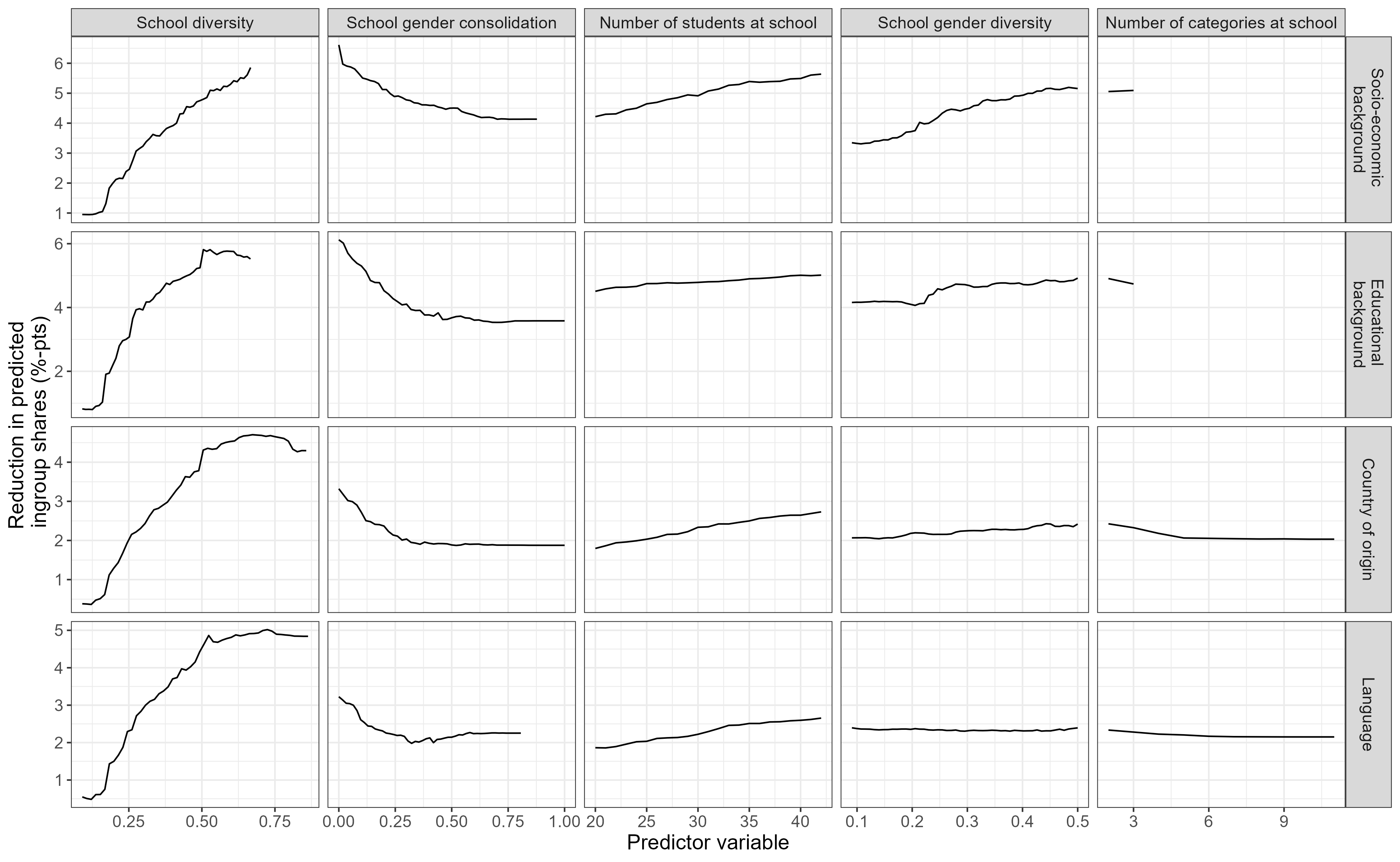

Supplement: S5 Fig — Results of random forests that predict simulated reductions in friendship segregation with gender consolidation minimizing class placements (compared to random class placements) for 16,117 schools in 79 countries with 5-fold cross-validation. (PNG) [file pone.0339581.s005.png]

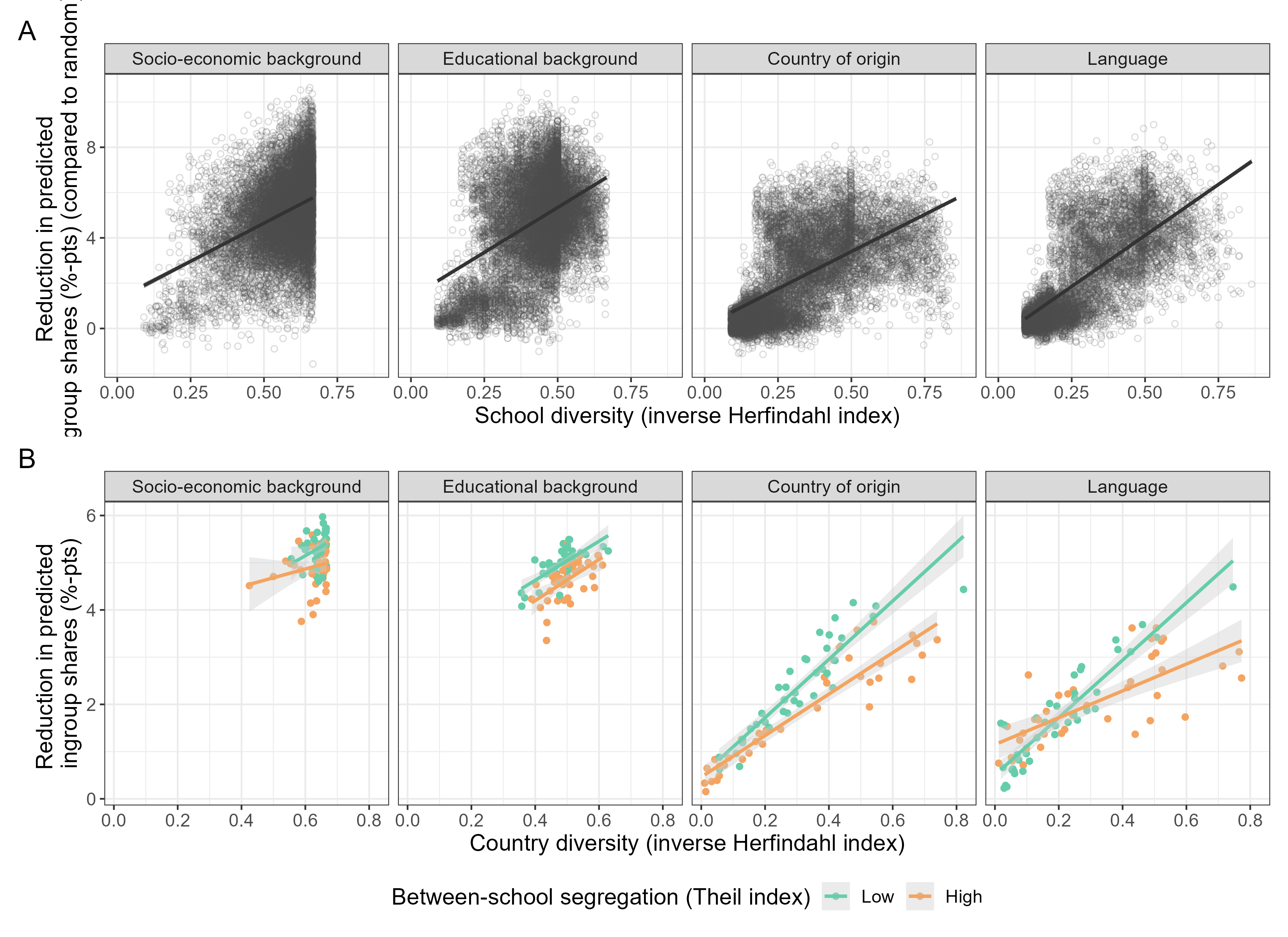

Supplement: S6 Fig — (A) Linear relationship of school diversity (measured as inverse Herfindahl index) and simulated reductions in friendship segregation. (B) Linear relationship between country diversity (measured as inverse Herfindahl index) and the country’s average simulated reduction in friendship segregation for countries with low (green) and high (orange) between-school segregation (Theil index below or above median). (PNG) [file pone.0339581.s006.png]
