# Supplementary material for: Friendship segregation and class composition in schools: A systematic analysis of the role of attribute consolidation
Source: PLoS One. 2025 Dec 31;20(12):e0339581. doi: 10.1371/journal.pone.0339581 (PMC12755804; doi:10.1371/journal.pone.0339581)
Supplement: S1 Text — (DOCX) [file pone.0339581.s007.docx]

S1 Text. Simulated class placement

In the second set of simulations in Study 2, based on data from PISA 2018, we proceed in the following three steps: First, we sort the students of each school to two classes with six different sorting strategies: a) Randomly, b) balancing genders across classes, and with a sorting heuristic that minimizes the consolidation of c) gender and socio-economic background, d) gender and educational background, e) gender and country of origin, and f) gender and language spoken at home. For sorting strategy b), we split the school population into two gender groups, shuffle them randomly within each gender group, and then assign students alternating to the two classes. In sorting strategies c) to f), we sort the students of each school according to the combination of gender and the selected attribute and assign students alternating to class 1 and 2 from the top to bottom of the sorted students list. With this simple heuristic, schools can sort the members of subgroups evenly to classes, thereby minimizing the consolidation of the two subgroup-defining attributes. We repeat each sorting strategy 200 times (randomly varying the order of subgroups in the student lists). Panel A in S2 Fig exemplarily visualizes two simulation rounds for one school in the dataset.

Second, we transform the data into a groups-in-class structure, where a group is defined as three or more students who are similar with respect to the selected attribute and have been sorted to the same school class. We do this separately for each of the four attributes (socio-economic background, educational background, country of origin, and language spoken at home), each of the ten imputed datasets, and each of the 1,200 simulated class placements. For each group, we calculate the consolidation of group membership with gender, the class size, group size, ingroup-outgroup diversity, gender diversity, and the absolute difference in the two diversity measures. Panel B in S2 Fig shows for each group in the example school how ingroup-outgroup diversity and gender consolidation differ between the sorting strategies.

Two details about the effect of the suggested sorting heuristic are observable in panel B in S2 Fig. It minimizes gender consolidation for the class with the *maximum* gender consolidation and it minimizes it at *class level* not at group level. The former means that by applying the sorting heuristic schools can avoid to end up with one class with a very strong gender consolidation. However, this is not the same as minimizing the average gender consolidation across classes or aiming at the lowest achievable gender consolidation in one class while ignoring the composition of the other classes. The red points in the bottom part of panel B in S2 Fig show that the maximum gender consolidation across classes is smallest for the sorting strategy *minimize consolidation*, while this is not necessarily the case for the minimum consolidation across classes (blue points). Similarly, the suggested sorting heuristic maximizes the diversity for the class with the minimum diversity, i.e. avoids classes with a very low diversity (see the rising blue points in the upper part of panel B in S2 Fig). The second aspect, that the suggested sorting heuristic minimizes consolidation at class and not group level, means that its impact can differ between the groups in a class. For example, the reduction in maximum consolidation values is considerably smaller for country of origin group 1 (red circles in the third graph at the bottom of panel B in S2 Fig) compared to country of origin groups 2 and 3 (red squares and triangles in the same graph). The suggested sorting strategy is a good heuristic to reduce the consolidation of attributes across classes and groups. However, if a secondary school is especially interested in increasing the chances for diverse friendships for a certain class or group at their school, another school-specific class placement may be more efficient.

Third, we use the models estimated in Study 1 to predict the shares of ingroup friends for all groups in the simulated school classes. We summarise these results by selecting within each simulated sorting strategy for each school in the dataset the maximum predicted ingroup share across groups and classes. This means that we evaluate the performance of a hypothetical class placement by taking into account only the predicted segregation for the most segregated group. We explicitly chose not to use the average or lowest predicted ingroup share across groups or classes, because we would argue that reducing segregation in one class or group at the expense of increasing segregation in another class or group is not desirable for secondary schools. Importantly, when selecting the maximum predicted ingroup share for each sorting strategy, we only take groups with non-missing predictions in both classes into account (for groups without any outgroup students in class Cramer’s V is not computable, leading to missing values in the ingroup share predictions). Note that this strategy of missing value handling leads to an underestimation of the achievable decrease in segregation as class placements with the largest differences in group sizes between classes are excluded from the comparison. For each school in the dataset, we compute the difference between the averages of the maximum predicted ingroup shares across the 200 gender consolidation minimizing and the 200 random class placements. In supplementary analyses we take the average over the maximum predicted ingroup shares across 200 gender balanced class placements as a benchmark of comparison. Panel C in S2 Fig illustrates the resulting predictions for the example school. We end up with one value per school and group-defining attribute that summarises the predicted reduction in friendship segregation achievable with the suggested sorting heuristic in comparison to random class placements (and for supplementary analyses one value comparing it to gender balanced class placements).
