# Supplementary material for: Friendship segregation and class composition in schools: A systematic analysis of the role of attribute consolidation
Source: PLoS One. 2025 Dec 31;20(12):e0339581. doi: 10.1371/journal.pone.0339581 (PMC12755804; doi:10.1371/journal.pone.0339581)
Supplement: S2 Text — (DOCX) [file pone.0339581.s008.docx]

S2 Text. Coding schemes in Studies 1 and 2

Two different data sources are used for Study 1 and 2, the “Children of Immigrants Longitudinal Survey in Four European Countries (CILS4EU)” and the “OECD Programme for International Student Assessment 2018 (PISA)”. To obtain comparable measures for the five attributes included in both studies, we harmonize information on students’ socio-economic background, educational background, country of origin, language spoken at home, country of origin, and gender.

In all analyses, students’ *socio-economic background* is defined as low, medium, or high based on the highest value of the parents’ International Socio-economic Index of Occupational Status (ISEI), with categorical cutoffs following the sample terciles in ISEI values across 79 countries in the PISA 2018 database (low if ISEI smaller 32.2, medium if ISEI larger or equal to 32.2 and smaller than 67.94, high if ISEI lager than 67.94).

Students’ *educational background* is defined in three categories as primary education or lower, secondary education, or tertiary education based on the highest educational attainment of students’ parents. In the analyses with CILS4EU data, this information is taken from parent reports (“What is your highest level of education?”, “What is your partner’s/husband’s/wife’s highest level of education?”) or student reports if the former are missing (“Did your father/mother complete primary school (or similar foreign education)?”, “Did your father/mother complete secondary school (or similar foreign education)?”,”Did your father/mother complete university?”). The answer categories given to parents who were asked about their education differed between the Netherlands and the other survey countries. We harmonize responses in the following way: We define the educational background as primary degree or lower if the highest degree reported by parents in Germany or Sweden is “I don’t have a school leaving certificate”, or by parents in the Netherlands “No education” or “Primary school”. If parents in Germany or Sweden respond with “Degree below upper secondary school” or “Degree from upper secondary school”, or parents in the Netherlands respond with “Secondary school”, “Lower vocational education”, or “Higher vocational education”, we define the educational background to be secondary degree. Finally, the educational degree is defined as tertiary degree if parents in Germany or Sweden respond with “University degree”, or parents in the Netherland respond with “University”. In the analyses with PISA data, information about students’ educational background is obtained from student reports (“What is the highest level of schooling completed by your mother/father?”, “Does your mother/father have any of the following qualifications?”) which are coded according to the International Standard Classification of Education 97 (ISCED) in the PISA database. To harmonize the categories with those used in the analyses with CILS4EU data, we define the educational background as primary or lower if the highest reported educational degree is none or ISCED level 1, as secondary if the highest reported degree is ISCED level 2, 3B, 3C, 3A, 4, or 5B, and as tertiary if the highest reported degree is ISCED level 5A or 6.

In all analyses, students’ *country of origin* is defined as the students’ country of birth if they were born abroad and the parents’ country of birth otherwise (i.e., the mother’s country of birth if she was born abroad and the fathers’ country of birth otherwise). The response options differed between the two surveys. While in CILS4EU respondents could select countries from a list or report another country in an open-ended question, in PISA 2018 students could select countries of birth from survey-country-specific lists without the option of open-ended responses. In the analyses with PISA data, we keep the ethnic categories of the survey countries, with Spain, China, and Great Britain being the only exceptions. These three countries use categories that are nested within the survey countries (e.g. provinces in Spain). We recode these categories to the respective survey country.

In the analyses with CILS4EU data, *language* is defined as the language spoken at students’ homes (“Is there a language other than [survey country language] spoken at your home?”) if students report to use this language often or always (“In this (second) language, how often do you talk to your family?”) and the survey country language otherwise. Again, a list of response categories were given to the students with the option to report another language in an open-ended question. In the analyses with PISA data, language is defined based on students’ answers to a similar question (“What language do you speak at home most of the time?”). Again, survey-country-specific lists with answer categories were given to the students without the option of open-ended responses. S3 Fig shows the number of countries of origin and languages in the 79 survey countries included in the second part of Study 2. We account for country differences in the number of countries of origin and languages by controlling for it when regressing the average reduction in simulated friendship segregation on country level characteristics.

In all analyses, *gender* is defined as a binary variable, as both surveys are limited to two gender categories. In CILS4EU students were asked “Are you a boy or a girl?” and in PISA 2018 they were asked “Are you female or male?”.
