# Supplementary material for: Friendship segregation and class composition in schools: A systematic analysis of the role of attribute consolidation
Source: PLoS One. 2025 Dec 31;20(12):e0339581. doi: 10.1371/journal.pone.0339581 (PMC12755804; doi:10.1371/journal.pone.0339581)
Supplement: S1 Table — (DOCX) [file pone.0339581.s009.docx]

S1 Table and Text. Gender segregation in adolescents’ school friendships around the world.

We search systematically for studies on gender segregation in adolescents’ friendship networks in schools around the world. For each country included in Study 2, we searched between December 2023 and January 2024 for estimates of gender segregation in school friendship networks of adolescents by checking the first 20 Google Scholar results (sorted by relevance) obtained with the search terms *friend school gender homophily [country name]*. S1 Table lists all 51 empirical studies conducted in schools in the respective country and containing a measure for gender segregation in adolescents’ friendships found with this approach. Reviewing these studies reveals a remarkable consistency of gender segregation in adolescents’ school friendships around the world. All 51 studies show a positive relationship between gender similarity and the likelihood of forming friendships.

**Table S1:** Studies with estimates of gender segregation in school friendships

| **Country** | **Study** |
| --- | --- |
| Albania | x |
| Argentina | x |
| Australia | K. De La Haye, G. Robins, P. Mohr, C. Wilson, Homophily and contagion as explanations for weight similarities among adolescent friends. *Journal of Adolescent Health* 49, 421–427 (2011). |
|  | J. Marks, K. De La Haye, L. M. Barnett, S. Allender, Friendship network characteristics are associated with physical activity and sedentary behavior in early adolescence. *PLoS ONE* 10, 1-15 (2015). |
| Austria | A. Garrote, C. L. A. Zurbriggen, S. Schwab, Friendship networks in inclusive elementary classrooms: Changes and stability related to students’ gender and self-perceived social inclusion. *Soc Psychol Educ* 26, 1479–1497 (2023). |
|  | S. Oczlon, et al., Ethnic in-and out-group friendships going into early adolescence: Prevalence, quality, stability, and the role of the network structure. *The Journal of Early Adolescence* 43, 867–907 (2023). |
|  | S. Hassani, S. Schwab, Z. Boda, Primary school students’ attitudes towards peers displaying hyperactivity: Examining impacts of homophily and inter-group contact on students’ social inclusion. *Social Development* 31, 765–781 (2022). |
| Azerbaijan | x |
| Belarus | x |
| Belgium | A. Grard, *et al*., Same-sex friendship, school gender composition, and substance use: A social network study of 50 European schools. *Substance Use & Misuse* 53, 998–1007 (2018). |
|  | H. Berten, R. Van Rossem, Homophily in adolescence: Is similarity in status characteristics associated with similarity in anti-school behaviour? *YOUNG* 23, 76–96 (2015). |
|  | C. Baerveldt, B. Zijlstra, M. de Wolf, R. Van Rossem, M. A. J. Van Duijn, Ethnic boundaries in high school students’ networks in Flanders and the Netherlands. *International Sociology* 22, 701–720 (2007). |
| Bosnia Herzegovina | x |
| Brazil | L. Alvarez, C. Pinto, V. Ponczek, Homophily in preferences or meetings? Identifying and estimating an iterative network formation model. arXiv [Preprint] (2023). http://arxiv.org/abs/2201.06694 (accessed 7 December 2023). |
| Brunei | x |
| Bulgaria | x |
| Canada | x |
| Chile | D. Chávez, D. Palacios, B. P. Luengo-Kanacri, C. Berger, G. Jiménez-Moya, The role of perspective-taking and low social class prejudice on cross-ethnic friendship formation. *New Directions for Child and Adolescent Development* 2021, 61–79 (2021). |
| China | W. An, Friendship network formation in Chinese middle schools: Patterns of inequality and homophily. *Social Networks* 68, 218–228 (2022). |
|  | M. Zhang, H. Liu, Y. Zhang, Adolescent social networks and physical, verbal, and indirect aggression in China: The moderating role of gender. *Frontiers in psychology* 11, 658 (2020). |
|  | C.-I. Wu, H.-H. Yang, Homophily effect of academic achievement on adolescent friendship network evolution. *MATEC Web Conf.* 71, 1-6 (2016). |
|  | H. Jiang, Essays on social capital and peer effects. (Doctoral dissertation, Virginia Tech, 2022). |
| Colombia | A. M. Guerra, et al., Effects of a physical activity program potentiated with ICTs on the formation and dissolution of friendship networks of children in a middle-income country. *International Journal of Environmental Research and Public Health* 17, 1-21 (2020). |
| Costa Rica | x |
| Croatia | x |
| Czech | T. Lintner, T. Diviák, K. Šeďová, P. Hlado, Ukrainian refugees struggling to integrate into Czech school social networks. *Humanit Soc Sci Commun* 10, 1-11 (2023). |
| Denmark | L. Mercken, T. A. B. Snijders, C. Steglich, H. De Vries, Dynamics of adolescent friendship networks and smoking behavior: Social network analyses in six European countries. *Social Science & Medicine* 69, 1506–1514 (2009). |
| Dominican Republic | x |
| Estonia | x |
| Finland | A. Grard, *et al*., Same-sex friendship, school gender composition, and substance use: A social network study of 50 European schools. *Substance Use & Misuse* 53, 998–1007 (2018). |
|  | E. Niskala, K. Sormunen, T. Palonen, T. Korhonen, K. Hakkarainen, Exploring Finnish fifth-grade pupils’ academic peer assistance networks. *Scandinavian Journal of Educational Research*, 1–18 (2023). |
|  | V.-J. Ilmarinen, M.-P. Vainikainen, M. J. Verkasalo, J.-E. Lönnqvist, Homophilous friendship assortment based on personality traits and cognitive ability in middle childhood: The moderating effect of peer network size. *European Journal of Personality* 31, 208–219 (2017). |
|  | S. Li, N. Kiuru, T. Palonen, K. Salmela-Aro, K. Hakkarainen, Peer selection and influence: Students’ interest-driven socio-digital participation and friendship networks. *FLR* 8, 1–17 (2020). |
| France | J. Stehlé, F. Charbonnier, T. Picard, C. Cattuto, A. Barrat, Gender homophily from spatial behavior in a primary school: A sociometric study. *Social Networks* 35, 604–613 (2013). |
|  | T. Chabot, How does socioeconomic homophily emerge? Testing for the contribution of different processes to socioeconomic segregation in adolescent friendships. *Social Networks* 76, 160–173 (2024). |
|  | P. Bhargava, D. L. Chen, M. Sutter, C. Terrier, Homophily and transmission of  behavioral traits in social networks. *CESifo Working Paper* No. 10351, 1-61 (2023) |
| Georgia | x |
| Greece | A. J. Umaña-Taylor, O. Kornienko, E. R. McDermott, F. Motti-Stefanidi, National identity development and friendship network dynamics among immigrant and non-immigrant youth. *J Youth Adolescence* 49, 706–723 (2020). |
| Hong Kong | T. J. Chan, C. T. Lam, Type of peers matters: A study of peer effects of friends, studymates and seatmates on academic performance. *Working paper Boston University*, 1-38 (2015) |
|  | A. K. Chan, A. K. Cheung, Gender differences in choosing STEM subjects at secondary school and university in Hong Kong. *Hong Kong: The Women’s Foundation,* 1-98 (2018) |
| Hungary | Z. Boda, Friendship based on race or race based on friendship? The co-evoluation of friendships, negative ties and ethnic perceptions in Hungarian school classes, (Doctoral dissertation, University of Oxford, 2015). |
|  | E. Vit, Peer effects on educational aspirations, (Doctoral dissertation, Budapesti Corvinus Egyetem, 2023). |
| Iceland | x |
| Indonesia | M. Shen, D. DeLay, U. Purwono, D. C. French, Peer relationships and Indonesian Muslim adolescents’ religiosity and religious coping: Selection and influence. *Journal of Research on Adolescence* 33, 127–140 (2023). |
| Ireland | M. McCann, J.-A. Jordan, K. Higgins, L. Moore, Longitudinal social network analysis of peer, family, and school contextual influences on adolescent drinking frequency. *Journal of Adolescent Health* 65, 350–358 (2019). |
|  | D. M. Kirke, Gender clustering in friendship networks: Some sociological implications. *Methodological Innovations Online* 4, 23–36 (2009). |
| Israel | U. Shwed, Y. Kalish, Y. Shavit, Multicultural or assimilationist education: Contact theory and social identity theory in Israeli Arab–Jewish integrated schools. *European Sociological Review* 34, 645–658 (2018). |
|  | G. Mesch, I. Talmud, The quality of online and offline relationships: The role of multiplexity and duration of social relationships. *The Information Society* 22, 137–148 (2006). |
| Italy | G. Rivellini, L. Terzera, V. Amati, Individual, dyadic and network effects in friendship relationships among Italian and foreign schoolmates. *Genus* 67 (2012). |
|  | A. Grard, *et al*., Same-sex friendship, school gender composition, and substance use: A social network study of 50 European schools. *Substance Use & Misuse* 53, 998–1007 (2018). |
| Japan | N. Maejima, Comparing gender homophily among the multilayer media social networks of face-to-face, instant messenger and social networking services: A case study of a high school classroom. *Connections* 40, 77–97 (2020). |
| Jordan | x |
| Kazakhstan | x |
| Korea | H. Shin, Friendship dynamics of adolescent aggression, prosocial behavior, and social status: The moderating role of gender. *J Youth Adolescence* 46, 2305–2320 (2017). |
|  | J. Kim, D. Park, Y.-J. Shin, Friendship dynamics of career decision-making self-efficacy: a longitudinal social network approach. *Curr Psychol* 42, 28771–28782 (2023). |
| Kosovo | x |
| Latvia | x |
| Lebanon | x |
| Lithuania | D. DeLay, G. Kaniušonytė, R. Žukauskienė, The social dynamics of fun: Can fun youth bring peers together and positively influence their friends? *J. Youth Adolescence* 53, 386–396 (2024). |
| Luxembourg | x |
| Macao | x |
| Malaysia | x |
| Malta | x |
| Mexico | R. Duarte-Barahona, E. Arceo-May, R. Huerta-Quintanilla, “Friendship formation in the classroom among elementary school students” in Complex Networks and Their Applications VIII, Studies in Computational Intelligence., H. Cherifi, S. Gaito, J. F. Mendes, E. Moro, L. M. Rocha, Eds. (Springer International Publishing, 2020), pp. 403–414. |
|  | D. B. Sánchez-Espinosa, E. Hernández-Ramírez, M. del Castillo-Mussot, Popularity and entropy in friendship and enmity networks in classrooms. *Entropy* 25, 971 (2023). |
| Moldova | x |
| Montenegro | x |
| Morocco | x |
| New Zealand | x |
| North Macedonia | x |
| Norway | x |
| Panama | x |
| Peru | x |
| Philippines | x |
| Poland | P. Grygiel, *et al*., The inclusion of other-sex peers in peer networks and sense of peer integration in early adolescence: A two-wave longitudinal study. *International Journal of Environmental Research and Public Health* 19, 1-24 (2022). |
|  | A. McKay, P. Grygiel, M. Karwowski, Connected to create: A social network analysis of friendship ties and creativity. *Psychology of Aesthetics, Creativity, and the Arts* 11, 284–294 (2017). |
| Portugal | A. Grard, et al., Same-sex friendship, school gender composition, and substance use: A social network study of 50 European schools. Substance Use & Misuse 53, 998–1007 (2018). |
|  | L. Mercken, T. A. B. Snijders, C. Steglich, H. De Vries, Dynamics of adolescent friendship networks and smoking behavior: Social network analyses in six European countries. *Social Science & Medicine* 69, 1506–1514 (2009). |
| Qatar | x |
| Romania | S. Baggio, V. Luisier, C. Vladescu, Relationships between social networks and mental health: An exponential random graph model approach among Romanian adolescents. *Swiss Journal of Psychology* 76, 5–11 (2017). |
| Russia |  |
|  | V. Ivaniushina, D. Alexandrov, Anti-school attitudes, school culture and friendship networks. *British Journal of Sociology of Education* 39, 698–716 (2018). |
| Saudi Arabia | x |
| Serbia | x |
| Slovak Republic | x |
| Slovenia | x |
| Spain | D. Escribano, V. Doldán-Martelli, F. J. Lapuente, J. A. Cuesta, A. Sánchez, Evolution of social relationships between first-year students at middle school: from cliques to circles. *Sci Rep* 11, 1-13 (2021). |
| Switzerland | x |
| Thailand | x |
| Turkey | x |
| Ukraine | x |
| United Arab Emirates | x |
| United States of America | W. Shrum, N. H. Cheek Jr, S. MacD, Friendship in school: Gender and racial homophily. *Sociology of Education*, 227–239 (1988). |
|  | P. Block, T. Grund, Multidimensional homophily in friendship networks. *Netw. sci.* 2, 189–212 (2014). |
| Uruguay | x |
| Vietnam | x |
| Studies with estimates of gender segregation in school friendship networks of adolescents within the 20 first Google Scholar results (sorted by relevance) obtained with the search terms *friend school gender homophily [country name]*. Inclusion criteria: Empirical study, conducted in schools in the respective country, containing measures of gender segregation in adolescents’ friendship networks. | |
