# Supplementary material for: Friendship segregation and class composition in schools: A systematic analysis of the role of attribute consolidation
Source: PLoS One. 2025 Dec 31;20(12):e0339581. doi: 10.1371/journal.pone.0339581 (PMC12755804; doi:10.1371/journal.pone.0339581)
Supplement: S2 Table — (DOCX) [file pone.0339581.s010.docx]

**Table S2:** OLS models regressing the share of ingroup friends on consolidation using only complete cases

|  |  | **Consolidating attribute** | | | | | | |
| --- | --- | --- | --- | --- | --- | --- | --- | --- |
|  | **Variable** | **Socio-econ. backgr.** | **Educat. backgr.** | **Country of origin** | **Religion** | **Language** | **Resident. area** | **Gender** |
| **Group-defining attribute:  Socio-economic background** |  |  |  |  |  |  |  |  |
|  | Intercept |  | 0.315*** | 0.236*** | 0.301*** | 0.25*** | 0.251*** | 0.211*** |
|  |  |  | (7.15) | (5.12) | (6.56) | (6.05) | (5.36) | (3.52) |
|  | Consolidation |  | 0.013 | 0.08* | 0.077** | 0.057 | 0.107** | 0.245*** |
|  |  |  | (0.58) | (2.26) | (2.86) | (1.67) | (2.93) | (8.28) |
|  | Class size |  | -0.02*** | -0.017*** | -0.019*** | -0.016*** | -0.018*** | -0.018*** |
|  |  |  | (-16.21) | (-10.42) | (-15.6) | (-11.59) | (-12.75) | (-15.28) |
|  | Group size |  | 0.053*** | 0.053*** | 0.053*** | 0.053*** | 0.053*** | 0.053*** |
|  |  |  | (31.81) | (31.32) | (30.82) | (31.1) | (31.51) | (30.77) |
|  | Ingr.-outgr. diversity |  | 0.067 | 0.076 | 0.061 | 0.064 | 0.097 | 0.146 |
|  |  |  | (0.98) | (1.09) | (0.84) | (0.95) | (1.25) | (1.67) |
|  | Diversity cons. attr. |  | -0.072 | 0.106 | 0.002 | 0.167*** | -0.031 | -0.066 |
|  |  |  | (-1.63) | (1.87) | (0.05) | (3.78) | (-0.75) | (-0.83) |
|  | Abs. diff. diversity |  | -0.118* | 0.004 | -0.014 | 0.022 | 0.039 | 0.137 |
|  |  |  | (-2.46) | (0.13) | (-0.31) | (0.66) | (0.82) | (1.67) |
|  | Number of categories |  | 0.011 | -0.014** | -0.005 | -0.021*** | -0.004 |  |
|  |  |  | (1.15) | (-3.06) | (-0.69) | (-4.55) | (-1.51) |  |
| **Group-defining attribute:  Educational background** |  |  |  |  |  |  |  |  |
|  | Intercept | 0.509*** |  | 0.452*** | 0.505*** | 0.481*** | 0.527*** | 0.492*** |
|  |  | (9.13) |  | (8) | (9.2) | (9.29) | (8.54) | (8.53) |
|  | Consolidation | 0.043 |  | 0.076** | 0.061* | 0.048* | 0.074* | 0.217*** |
|  |  | (1.88) |  | (3.14) | (2.25) | (2.07) | (2.41) | (7.53) |
|  | Class size | -0.026*** |  | -0.025*** | -0.026*** | -0.025*** | -0.026*** | -0.025*** |
|  |  | (-17.11) |  | (-14.69) | (-16.45) | (-15.36) | (-15.76) | (-18.52) |
|  | Group size | 0.046*** |  | 0.047*** | 0.046*** | 0.046*** | 0.047*** | 0.047*** |
|  |  | (28.38) |  | (28.68) | (28.62) | (28.43) | (28.59) | (29.29) |
|  | Ingr.-outgr. diversity | -0.063 |  | -0.113* | -0.134** | -0.114* | -0.159* | -0.093 |
|  |  | (-0.41) |  | (-2.17) | (-2.8) | (-2.37) | (-2.46) | (-1.24) |
|  | Diversity cons. attr. | -0.043 |  | 0.015 | 0.019 | 0.027 | -0.058 | -0.132* |
|  |  | (-0.31) |  | (0.3) | (0.5) | (0.64) | (-1.41) | (-2.09) |
|  | Abs. diff. diversity | 0.067 |  | -0.012 | -0.04 | 0.019 | -0.041 | 0.014 |
|  |  | (0.44) |  | (-0.31) | (-0.96) | (0.57) | (-0.89) | (0.2) |
|  | Number of categories | -0.011 |  | -0.005 | -0.01 | -0.009 | 0 |  |
|  |  | (-0.68) |  | (-1.3) | (-1.39) | (-1.71) | (-0.04) |  |
| **Group-defining attribute:  Country of origin** |  |  |  |  |  |  |  |  |
|  | Intercept | 0.757*** | 0.8*** |  | 0.798*** | 0.588*** | 0.741*** | 0.562*** |
|  |  | (9.18) | (14.5) |  | (16.64) | (7.55) | (11.16) | (7.6) |
|  | Consolidation | 0.085* | 0.077* |  | 0.163*** | 0.229*** | 0.119** | 0.248*** |
|  |  | (2.36) | (2.19) |  | (5.83) | (5) | (3.17) | (6.46) |
|  | Class size | -0.024*** | -0.023*** |  | -0.023*** | -0.021*** | -0.022*** | -0.022*** |
|  |  | (-10.77) | (-10) |  | (-10.11) | (-8.21) | (-9.71) | (-10.91) |
|  | Group size | 0.046*** | 0.045*** |  | 0.046*** | 0.042*** | 0.045*** | 0.045*** |
|  |  | (23.35) | (22.16) |  | (21.95) | (16.25) | (23.45) | (24.71) |
|  | Ingr.-outgr. diversity | 0.303* | 0.115* |  | 0.071 | 0.181** | 0.099 | 0.139 |
|  |  | (2.3) | (1.96) |  | (0.96) | (2.76) | (1.18) | (1.35) |
|  | Diversity cons. attr. | -0.076 | -0.038 |  | 0.017 | -0.022 | 0.028 | 0.159 |
|  |  | (-0.55) | (-0.72) |  | (0.29) | (-0.33) | (0.46) | (1.69) |
|  | Abs. diff. diversity | 0.21 | 0.084 |  | -0.046 | -0.003 | 0.01 | 0.032 |
|  |  | (1.53) | (1.34) |  | (-0.65) | (-0.04) | (0.14) | (0.3) |
|  | Number of categories | -0.014 | -0.005 |  | -0.024* | -0.008 | -0.004 |  |
|  |  | (-0.64) | (-0.35) |  | (-2.48) | (-1.34) | (-1.11) |  |
| **Group-defining attribute:  Religion** |  |  |  |  |  |  |  |  |
|  | Intercept | 0.506*** | 0.453*** | 0.411*** |  | 0.442*** | 0.411*** | 0.306*** |
|  |  | (7.89) | (10.38) | (8.79) |  | (10.78) | (8.09) | (5.07) |
|  | Consolidation | 0.104** | 0.057 | 0.123*** |  | 0.101*** | 0.144*** | 0.275*** |
|  |  | (3.14) | (1.84) | (3.95) |  | (3.3) | (3.32) | (8) |
|  | Class size | -0.023*** | -0.024*** | -0.02*** |  | -0.022*** | -0.021*** | -0.022*** |
|  |  | (-15.39) | (-15.79) | (-11.54) |  | (-13.04) | (-12.27) | (-15.67) |
|  | Group size | 0.049*** | 0.049*** | 0.048*** |  | 0.049*** | 0.049*** | 0.049*** |
|  |  | (40.57) | (40.4) | (38.9) |  | (39.05) | (40.49) | (40.7) |
|  | Ingr.-outgr. diversity | 0.107 | 0.049 | 0.08 |  | 0.068 | 0.077 | 0.127 |
|  |  | (0.75) | (0.99) | (1.61) |  | (1.39) | (1.15) | (1.42) |
|  | Diversity cons. attr. | 0.052 | -0.022 | 0.07 |  | 0.084 | 0.001 | 0.153* |
|  |  | (0.46) | (-0.44) | (1.03) |  | (1.41) | (0.02) | (1.96) |
|  | Abs. diff. diversity | 0.049 | 0 | -0.016 |  | -0.007 | 0.017 | 0.084 |
|  |  | (0.33) | (0) | (-0.36) |  | (-0.15) | (0.32) | (0.83) |
|  | Number of categories | -0.038 | 0.016 | -0.017*** |  | -0.019** | -0.009* |  |
|  |  | (-1.74) | (1.27) | (-3.3) |  | (-3.22) | (-2.5) |  |
| **Group-defining attribute:  Language** |  |  |  |  |  |  |  |  |
|  | Intercept | 0.503*** | 0.683*** | 0.58*** | 0.66*** |  | 0.645*** | 0.636*** |
|  |  | (6.61) | (8.35) | (7.91) | (10.42) |  | (8.45) | (9.54) |
|  | Consolidation | 0.096** | 0.059 | 0.159*** | 0.11*** |  | 0.008 | 0.23*** |
|  |  | (2.92) | (1.94) | (4.42) | (4.86) |  | (0.27) | (5) |
|  | Class size | -0.026*** | -0.023*** | -0.022*** | -0.024*** |  | -0.024*** | -0.023*** |
|  |  | (-11.66) | (-9.76) | (-8.47) | (-10.21) |  | (-9.73) | (-10.03) |
|  | Group size | 0.045*** | 0.045*** | 0.041*** | 0.046*** |  | 0.045*** | 0.045*** |
|  |  | (16.83) | (15.79) | (14.39) | (16.18) |  | (16.13) | (16.37) |
|  | Ingr.-outgr. diversity | 0.243* | 0.006 | 0.177 | -0.102 |  | 0.01 | -0.146 |
|  |  | (2.13) | (0.09) | (1.84) | (-1.59) |  | (0.12) | (-1.31) |
|  | Diversity cons. attr. | 0.023 | -0.008 | -0.216* | 0.099 |  | 0.035 | 0.094 |
|  |  | (0.21) | (-0.15) | (-2.26) | (1.72) |  | (0.49) | (0.99) |
|  | Abs. diff. diversity | 0.279* | 0.019 | 0.049 | -0.149* |  | 0.021 | -0.144 |
|  |  | (2.44) | (0.35) | (0.5) | (-2.4) |  | (0.3) | (-1.42) |
|  | Number of categories | 0.031 | -0.018 | 0 | -0.016 |  | -0.001 |  |
|  |  | (1.32) | (-1.26) | (-0.04) | (-1.74) |  | (-0.23) |  |
| **Group-defining attribute:  Residential area** |  |  |  |  |  |  |  |  |
|  | Intercept | 0.447*** | 0.364*** | 0.295*** | 0.364*** | 0.315*** |  | 0.347*** |
|  |  | (5.75) | (5.95) | (4.94) | (6.11) | (5.89) |  | (4.51) |
|  | Consolidation | 0.064 | 0.055 | 0.09 | 0.06 | 0.087 |  | 0.395*** |
|  |  | (1.34) | (1.15) | (1.9) | (1.36) | (1.87) |  | (11) |
|  | Class size | -0.014*** | -0.015*** | -0.012*** | -0.015*** | -0.013*** |  | -0.015*** |
|  |  | (-7.22) | (-7.89) | (-5.58) | (-8.02) | (-6.52) |  | (-8.72) |
|  | Group size | 0.044*** | 0.043*** | 0.044*** | 0.044*** | 0.044*** |  | 0.046*** |
|  |  | (28.99) | (26.94) | (29.74) | (29.17) | (29.4) |  | (29.45) |
|  | Ingr.-outgr. diversity | 0.224 | 0.057 | 0.048 | 0.037 | 0.019 |  | 0.016 |
|  |  | (1.15) | (0.93) | (0.74) | (0.54) | (0.3) |  | (0.16) |
|  | Diversity cons. attr. | -0.132 | 0.033 | 0.068 | 0.11* | 0.093 |  | -0.161 |
|  |  | (-0.69) | (0.57) | (0.93) | (2.01) | (1.41) |  | (-1.56) |
|  | Abs. diff. diversity | 0.215 | 0.113 | 0.061 | 0.051 | -0.043 |  | 0.076 |
|  |  | (1.11) | (1.81) | (1.22) | (0.81) | (-0.88) |  | (0.7) |
|  | Number of categories | -0.044 | -0.007 | -0.009 | -0.016 | -0.005 |  |  |
|  |  | (-1.57) | (-0.45) | (-1.41) | (-1.53) | (-0.65) |  |  |
| **Group-defining attribute:  Gender** |  |  |  |  |  |  |  |  |
|  | Intercept | 0.641*** | 0.609*** | 0.622*** | 0.628*** | 0.568*** | 0.596*** |  |
|  |  | (8) | (9.07) | (11.39) | (10.69) | (9.7) | (8.7) |  |
|  | Consolidation | 0.061 | 0.037 | -0.088 | 0.06 | -0.051 | 0.06 |  |
|  |  | (1.7) | (1.13) | (-1.67) | (1.73) | (-1.01) | (1.17) |  |
|  | Class size | -0.007*** | -0.008*** | -0.009*** | -0.008*** | -0.007*** | -0.006*** |  |
|  |  | (-4.6) | (-5.43) | (-4.79) | (-5.13) | (-4.11) | (-3.78) |  |
|  | Group size | 0.023*** | 0.023*** | 0.023*** | 0.023*** | 0.023*** | 0.023*** |  |
|  |  | (12.51) | (12.54) | (12.56) | (12.48) | (12.5) | (12.47) |  |
|  | Ingr.-outgr. diversity | 0.346* | 0.378*** | 0.41*** | 0.366*** | 0.38*** | 0.364*** |  |
|  |  | (2.48) | (3.8) | (4.15) | (3.68) | (3.84) | (3.46) |  |
|  | Diversity cons. attr. | 0.061 | 0.095 | 0.038 | -0.022 | 0.112* | -0.003 |  |
|  |  | (0.71) | (1.61) | (0.74) | (-0.5) | (2.3) | (-0.07) |  |
|  | Abs. diff. diversity | -0.036 | 0.014 | 0.035 | -0.012 | 0.089* | -0.013 |  |
|  |  | (-0.35) | (0.23) | (0.88) | (-0.21) | (2.17) | (-0.26) |  |
|  | Number of categories | -0.027 | -0.017 | 0.001 | -0.008 | -0.005 | -0.005 |  |
|  |  | (-1.39) | (-1.62) | (0.18) | (-1.02) | (-0.84) | (-1.63) |  |
| Unstandardized coefficients and t-values in parentheses of OLS regressions with cluster robust standard errors and groups-in-survey-countries fixed effects. Case-wise deletion of missing values. ***p<0.001 **p<0.01 *p<0.05. Ingr.-outgr. Diversity = Ingroup-outgroup diversity; Diversity cons. attr. = Diversity of the consolidating attribute; Abs. diff. diversity = Absolute difference between ingroup-outgroup diversity and diversity in the consolidating attribute. | | | | | | | | |
