# Supplementary material for: Friendship segregation and class composition in schools: A systematic analysis of the role of attribute consolidation
Source: PLoS One. 2025 Dec 31;20(12):e0339581. doi: 10.1371/journal.pone.0339581 (PMC12755804; doi:10.1371/journal.pone.0339581)
Supplement: S4 Table — (DOCX) [file pone.0339581.s012.docx]

**Table S4:** OLS models regressing the share of ingroup friends on consolidation, including six groups without any friendship nominations (setting their ingroup share to 1)

|  |  | **Consolidating attribute** | | | | | | | |
| --- | --- | --- | --- | --- | --- | --- | --- | --- | --- |
|  | **Variable** | **Socio-econ. backgr.** | **Educat. backgr.** | **Country of origin** | **Religion** | **Language** | **Resident. area** | **Gender** | |
| **Group-defining attribute:  Socio-economic background** |  |  |  |  |  |  |  |  | |
|  | Intercept |  | 0.29*** | 0.222*** | 0.269*** | 0.243*** | 0.271*** | 0.231*** | |
|  |  |  | (7.95) | (5.6) | (7.31) | (6.22) | (6.57) | (3.8) | |
|  | Consolidation |  | 0.026 | 0.081* | 0.059* | 0.072 | 0.076* | 0.227*** | |
|  |  |  | (1.24) | (2.58) | (2.21) | (1.95) | (2.08) | (8.46) | |
|  | Class size |  | -0.016*** | -0.014*** | -0.016*** | -0.014*** | -0.015*** | -0.015*** | |
|  |  |  | (-16.55) | (-11.43) | (-16.15) | (-11.5) | (-11.9) | (-15.36) | |
|  | Group size |  | 0.047*** | 0.047*** | 0.047*** | 0.047*** | 0.047*** | 0.047*** | |
|  |  |  | (34.72) | (33.98) | (33.48) | (33.65) | (33.91) | (34.86) | |
|  | Ingr.-outgr. diversity |  | 0.043 | 0.076 | 0.063 | 0.062 | 0.029 | 0.122 | |
|  |  |  | (0.72) | (1.23) | (0.97) | (1.02) | (0.43) | (1.51) | |
|  | Diversity cons. attr. |  | -0.029 | 0.043 | 0.016 | 0.063 | -0.001 | -0.105 | |
|  |  |  | (-0.85) | (1.2) | (0.49) | (1.83) | (-0.02) | (-1.49) | |
|  | Abs. diff. diversity |  | -0.044 | 0.021 | 0.02 | 0.014 | -0.027 | 0.105 | |
|  |  |  | (-1.13) | (0.64) | (0.47) | (0.49) | (-0.81) | (1.3) | |
|  | Number of categories |  | 0.003 | -0.008** | -0.004 | -0.01* | -0.002 |  | |
|  |  |  | (0.4) | (-2.58) | (-0.62) | (-2.43) | (-1) |  | |
| **Group-defining attribute:  Educational background** |  |  |  |  |  |  |  |  | |
|  | Intercept | 0.526*** |  | 0.475*** | 0.477*** | 0.49*** | 0.533*** | 0.506*** | |
|  |  | (8.46) |  | (10) | (11.3) | (10.97) | (10.67) | (8.98) | |
|  | Consolidation | 0.053* |  | 0.089*** | 0.06* | 0.046 | 0.049 | 0.253*** | |
|  |  | (2.43) |  | (3.34) | (2.37) | (1.62) | (1.75) | (7.53) | |
|  | Class size | -0.021*** |  | -0.021*** | -0.021*** | -0.021*** | -0.021*** | -0.02*** | |
|  |  | (-16.82) |  | (-15.49) | (-16.3) | (-15.58) | (-15.09) | (-16.83) | |
|  | Group size | 0.041*** |  | 0.041*** | 0.041*** | 0.041*** | 0.041*** | 0.041*** | |
|  |  | (35.35) |  | (35.65) | (35.6) | (34.99) | (35.46) | (35.95) | |
|  | Ingr.-outgr. diversity | -0.124 |  | -0.083 | -0.059 | -0.092* | -0.135* | -0.164 | |
|  |  | (-1.1) |  | (-1.81) | (-1.35) | (-2.21) | (-2.47) | (-1.92) | |
|  | Diversity cons. attr. | 0.004 |  | -0.052 | -0.007 | -0.003 | -0.037 | -0.087 | |
|  |  | (0.03) |  | (-1.32) | (-0.22) | (-0.08) | (-1) | (-1.45) | |
|  | Abs. diff. diversity | -0.012 |  | 0.008 | 0.063 | -0.002 | -0.032 | -0.081 | |
|  |  | (-0.11) |  | (0.21) | (1.5) | (-0.08) | (-0.87) | (-0.94) | |
|  | Number of categories | -0.008 |  | 0 | -0.006 | -0.003 | 0 |  | |
|  |  | (-0.38) |  | (0.15) | (-0.93) | (-0.65) | (-0.03) |  | |
| **Group-defining attribute:  Country of origin** |  |  |  |  |  |  |  |  | |
|  | Intercept | 0.638*** | 0.69*** |  | 0.706*** | 0.64*** | 0.634*** | 0.517*** | |
|  |  | (7.38) | (14.56) |  | (15.43) | (9.94) | (11.21) | (6.63) | |
|  | Consolidation | 0.086* | 0.064 |  | 0.143*** | 0.164*** | 0.091* | 0.248*** | |
|  |  | (2.57) | (1.88) |  | (5.41) | (4.48) | (2.49) | (7) | |
|  | Class size | -0.015*** | -0.015*** |  | -0.014*** | -0.013*** | -0.014*** | -0.014*** | |
|  |  | (-9.23) | (-8.88) |  | (-8.87) | (-7.44) | (-7.98) | (-8.76) | |
|  | Group size | 0.04*** | 0.04*** |  | 0.04*** | 0.035*** | 0.04*** | 0.039*** | |
|  |  | (29.68) | (29.19) |  | (27.81) | (21.04) | (29.01) | (30.31) | |
|  | Ingr.-outgr. diversity | 0.255* | 0.105* |  | 0.048 | 0.093 | 0.124 | 0.085 | |
|  |  | (2) | (2.04) |  | (0.8) | (1.66) | (1.74) | (1) | |
|  | Diversity cons. attr. | -0.101 | 0.016 |  | -0.035 | -0.062 | -0.018 | 0.087 | |
|  |  | (-0.9) | (0.33) |  | (-0.69) | (-1.09) | (-0.3) | (1.15) | |
|  | Abs. diff. diversity | 0.174 | 0.071 |  | -0.041 | -0.172** | 0.06 | 0.009 | |
|  |  | (1.36) | (1.32) |  | (-0.64) | (-3.16) | (0.91) | (0.1) | |
|  | Number of categories | 0.007 | -0.006 |  | -0.015* | 0 | -0.003 |  | |
|  |  | (0.21) | (-0.47) |  | (-2.03) | (-0.02) | (-0.87) |  | |
| **Group-defining attribute:  Religion** |  |  |  |  |  |  |  |  | |
|  | Intercept | 0.475*** | 0.444*** | 0.394** |  | 0.433** | 0.364*** | 0.347*** | |
|  |  | (3.43) | (3.47) | (2.88) |  | (3.17) | (3.76) | (3.49) | |
|  | Consolidation | 0.085** | 0.036 | 0.137*** |  | 0.112*** | 0.156*** | 0.264*** | |
|  |  | (2.61) | (1.28) | (4.5) |  | (4.13) | (3.85) | (7.6) | |
|  | Class size | -0.017*** | -0.018*** | -0.015*** |  | -0.016*** | -0.015*** | -0.017*** | |
|  |  | (-14.27) | (-14.4) | (-10.79) |  | (-12.11) | (-10.63) | (-14.37) | |
|  | Group size | 0.043*** | 0.043*** | 0.043*** |  | 0.043*** | 0.043*** | 0.043*** | |
|  |  | (47.52) | (47.15) | (44.19) |  | (45.47) | (47.47) | (48.72) | |
|  | Ingr.-outgr. diversity | 0.088 | 0.086 | 0.114* |  | 0.094* | 0.109 | 0.133 | |
|  |  | (0.77) | (1.94) | (2.41) |  | (2.12) | (1.89) | (1.84) | |
|  | Diversity cons. attr. | 0.043 | 0.019 | 0.014 |  | 0.015 | 0.013 | 0.039 | |
|  |  | (0.45) | (0.45) | (0.29) |  | (0.36) | (0.31) | (0.53) | |
|  | Abs. diff. diversity | -0.002 | 0.011 | -0.007 |  | -0.032 | 0.027 | 0.052 | |
|  |  | (-0.01) | (0.23) | (-0.17) |  | (-0.8) | (0.59) | (0.65) | |
|  | Number of categories | -0.019 | 0.004 | -0.012*** |  | -0.009* | -0.008** |  | |
|  |  | (-0.72) | (0.4) | (-3.78) |  | (-2.28) | (-2.81) |  | |
| **Group-defining attribute:  Language** |  |  |  |  |  |  |  |  | |
|  | Intercept | 0.4*** | 0.489*** | 0.464*** | 0.494*** |  | 0.477*** | 0.419*** | |
|  |  | (4.78) | (7.47) | (6.26) | (8.64) |  | (7.95) | (6.14) | |
|  | Consolidation | 0.064 | 0.033 | 0.12*** | 0.086*** |  | 0.04 | 0.248*** | |
|  |  | (1.85) | (1.11) | (3.29) | (3.55) |  | (1.45) | (7.41) | |
|  | Class size | -0.02*** | -0.019*** | -0.016*** | -0.018*** |  | -0.018*** | -0.018*** | |
|  |  | (-11.7) | (-11) | (-7.7) | (-10.9) |  | (-10.64) | (-11.05) | |
|  | Group size | 0.041*** | 0.041*** | 0.037*** | 0.041*** |  | 0.041*** | 0.041*** | |
|  |  | (28.64) | (28.46) | (21.85) | (27.71) |  | (28.7) | (29.63) | |
|  | Ingr.-outgr. diversity | 0.095 | 0.065 | 0.128 | -0.008 |  | 0.063 | -0.026 | |
|  |  | (0.69) | (1.32) | (1.54) | (-0.13) |  | (0.85) | (-0.3) | |
|  | Diversity cons. attr. | -0.004 | -0.028 | -0.117 | 0.034 |  | -0.029 | 0.068 | |
|  |  | (-0.03) | (-0.6) | (-1.33) | (0.59) |  | (-0.45) | (0.84) | |
|  | Abs. diff. diversity | 0.076 | 0.094 | -0.087 | -0.032 |  | 0.056 | -0.055 | |
|  |  | (0.55) | (1.86) | (-0.99) | (-0.48) |  | (0.81) | (-0.65) | |
|  | Number of categories | 0.025 | -0.006 | -0.003 | -0.013 |  | -0.003 |  | |
|  |  | (0.98) | (-0.45) | (-0.51) | (-1.49) |  | (-0.96) |  | |
| **Group-defining attribute:  Residential area** |  |  |  |  |  |  |  |  | |
|  | Intercept | 0.371*** | 0.308*** | 0.249*** | 0.288*** | 0.273*** |  | 0.282*** | |
|  |  | (5.39) | (5.97) | (4.87) | (5.78) | (5.87) |  | (4.47) | |
|  | Consolidation | 0.01 | 0.063 | 0.062 | 0.096* | 0.087* |  | 0.416*** | |
|  |  | (0.22) | (1.37) | (1.57) | (2.5) | (2.28) |  | (14.48) | |
|  | Class size | -0.012*** | -0.012*** | -0.01*** | -0.011*** | -0.011*** |  | -0.01*** | |
|  |  | (-7.36) | (-7.79) | (-5.74) | (-7.8) | (-6.51) |  | (-7.28) | |
|  | Group size | 0.038*** | 0.038*** | 0.038*** | 0.039*** | 0.038*** |  | 0.04*** | |
|  |  | (30.61) | (28.56) | (30.26) | (30.67) | (29.88) |  | (32.87) | |
|  | Ingr.-outgr. diversity | 0.198 | 0.11* | 0.126* | 0.13* | 0.089 |  | 0.071 | |
|  |  | (1.04) | (2.1) | (2.33) | (2.11) | (1.76) |  | (0.8) | |
|  | Diversity cons. attr. | -0.113 | -0.018 | 0.043 | 0.025 | 0.034 |  | -0.209** | |
|  |  | (-0.61) | (-0.36) | (0.76) | (0.51) | (0.64) |  | (-2.71) | |
|  | Abs. diff. diversity | 0.101 | 0.033 | 0.046 | 0.074 | -0.017 |  | 0.049 | |
|  |  | (0.52) | (0.6) | (1.08) | (1.26) | (-0.42) |  | (0.54) | |
|  | Number of categories | -0.017 | -0.001 | -0.005 | -0.011 | -0.002 |  |  | |
|  |  | (-0.64) | (-0.08) | (-1.15) | (-1.19) | (-0.44) |  |  | |
| **Group-defining attribute:  Gender** |  |  |  |  |  |  |  |  | |
|  | Intercept | 0.68*** | 0.661*** | 0.669*** | 0.696*** | 0.623*** | 0.599*** |  | |
|  |  | (7.38) | (12.3) | (13.91) | (13.01) | (12.22) | (8.96) |  | |
|  | Consolidation | 0.026 | 0.009 | -0.09* | 0.052 | -0.001 | 0.091* |  | |
|  |  | (0.74) | (0.26) | (-2.07) | (1.6) | (-0.01) | (2.29) |  | |
|  | Class size | -0.006*** | -0.006*** | -0.006*** | -0.005*** | -0.004** | -0.003* |  | |
|  |  | (-3.91) | (-4.1) | (-3.65) | (-3.85) | (-3) | (-2.07) |  | |
|  | Group size | 0.019*** | 0.019*** | 0.019*** | 0.019*** | 0.019*** | 0.019*** |  | |
|  |  | (12.47) | (12.48) | (12.55) | (12.46) | (12.49) | (12.45) |  | |
|  | Ingr.-outgr. diversity | 0.22 | 0.219* | 0.26** | 0.209* | 0.206* | 0.246* |  | |
|  |  | (1.58) | (2.28) | (2.74) | (2.18) | (2.2) | (2.37) |  | |
|  | Diversity cons. attr. | -0.059 | 0.082 | 0.064 | 0.003 | 0.094* | 0.015 |  | |
|  |  | (-0.72) | (1.57) | (1.6) | (0.08) | (2.48) | (0.34) |  | |
|  | Abs. diff. diversity | -0.008 | 0.052 | 0.038 | -0.009 | 0.081* | 0.023 |  | |
|  |  | (-0.07) | (0.81) | (1.12) | (-0.17) | (2.27) | (0.5) |  | |
|  | Number of categories | 0.007 | -0.009 | -0.001 | -0.013 | -0.006 | -0.007** |  | |
|  |  | (0.25) | (-0.88) | (-0.32) | (-1.87) | (-1.42) | (-2.84) |  | |
| Unstandardized coefficients and t-values in parentheses of OLS regressions with cluster robust standard errors and groups-in-survey-countries fixed effects. Pooled results over ten imputations using Rubin’s rules. ***p<0.001 **p<0.01 *p<0.05. Ingr.-outgr. Diversity = Ingroup-outgroup diversity; Diversity cons. attr. = Diversity of the consolidating attribute; Abs. diff. diversity = Absolute difference between ingroup-outgroup diversity and diversity in the consolidating attribute. | | | | | | | | |  |
