# Supplementary material for: Friendship segregation and class composition in schools: A systematic analysis of the role of attribute consolidation
Source: PLoS One. 2025 Dec 31;20(12):e0339581. doi: 10.1371/journal.pone.0339581 (PMC12755804; doi:10.1371/journal.pone.0339581)
Supplement: S5 Table — (DOCX) [file pone.0339581.s013.docx]

**Table S5:** Missingness and number of categories in the seven group-defining attributes

|  | **Individual-level data** | | **Group-level data** | | | | | | |
| --- | --- | --- | --- | --- | --- | --- | --- | --- | --- |
|  |  |  | **Categories per class** | | | | **Categories within classes** | | |
| **Group-defining attribute** | **Obs.** | **Missing values** | **Mean** | **Std. Dev.** | **Min.** | **Max.** | **Total** | **Groups  (at least 3 students)** | **Groups (final sample)** |
| Socio-economic background | 16714 | 2002 | 2.87 | 0.35 | 1 | 3 | 2732 | 2345 | 1348 |
| Educational background | 18032 | 684 | 2.52 | 0.59 | 1 | 3 | 2402 | 1808 | 984 |
| Country of origin | 18525 | 191 | 6.03 | 3.11 | 1 | 22 | 5743 | 1376 | 804 |
| Religion | 17726 | 990 | 2.97 | 0.92 | 1 | 7 | 2832 | 1912 | 1106 |
| Language | 18195 | 521 | 4.12 | 2.49 | 1 | 17 | 3918 | 1337 | 800 |
| Residential area | 18716 | 0 | 9.48 | 4.66 | 1 | 31 | 9025 | 1473 | 948 |
| Gender | 18702 | 14 | 1.9 | 0.3 | 1 | 2 | 1811 | 1753 | 1033 |
| Missing values are imputed ten times using multivariate imputation by chained equations. The summary statistics for group-level data are based on the first imputed dataset. Information on the other imputed datasets is available on request from the authors. | | | | | | | | | |
