# Supplementary material for: Friendship segregation and class composition in schools: A systematic analysis of the role of attribute consolidation
Source: PLoS One. 2025 Dec 31;20(12):e0339581. doi: 10.1371/journal.pone.0339581 (PMC12755804; doi:10.1371/journal.pone.0339581)
Supplement: S6 Table — (DOCX) [file pone.0339581.s014.docx]

**Table S6:** Summary statistics of the analytical samples for the seven group-defining attributes

| **Variable** |  | **Mean** | **Std. Dev.** | **Min.** | **Max.** |  | **Mean** | **Std. Dev.** | **Min.** | **Max.** |
| --- | --- | --- | --- | --- | --- | --- | --- | --- | --- | --- |
| Ingroup share | **Group-defining attribute: Socio-economic background** | 0.36 | 0.2 | 0 | 0.96 | **Group-defining attribute: Educational background** | 0.49 | 0.28 | 0 | 1 |
| Class size |  | 21.53 | 4.32 | 10 | 32 |  | 21.43 | 4.34 | 10 | 32 |
| Group size |  | 8.03 | 3.6 | 3 | 22 |  | 10.78 | 5.87 | 3 | 28 |
| Ingr.-outgr. diversity |  | 0.42 | 0.08 | 0.1 | 0.5 |  | 0.37 | 0.11 | 0.07 | 0.5 |
| Consolidation with socio-economic backgr. |  |  |  |  |  |  | 0.42 | 0.2 | 0 | 1 |
| Consolidation with educational backgr. |  | 0.34 | 0.18 | 0 | 1 |  |  |  |  |  |
| Consolidation with country of origin |  | 0.54 | 0.19 | 0.01 | 1 |  | 0.57 | 0.21 | 0.04 | 1 |
| Consolidation with religion |  | 0.32 | 0.16 | 0 | 0.8 |  | 0.32 | 0.18 | 0 | 1 |
| Consolidation with language |  | 0.43 | 0.18 | 0.01 | 1 |  | 0.46 | 0.22 | 0.01 | 1 |
| Consolidation with residential area |  | 0.61 | 0.19 | 0.03 | 1 |  | 0.59 | 0.21 | 0.01 | 1 |
| Consolidation with gender |  | 0.18 | 0.14 | 0 | 0.75 |  | 0.19 | 0.13 | 0 | 0.78 |
| Diversity socio-economic background |  |  |  |  |  |  | 0.57 | 0.08 | 0.1 | 0.67 |
| Diversity educational background |  | 0.4 | 0.15 | 0.07 | 0.66 |  |  |  |  |  |
| Diversity country of origin |  | 0.55 | 0.23 | 0.08 | 0.95 |  | 0.57 | 0.22 | 0.08 | 0.95 |
| Diversity religion |  | 0.48 | 0.14 | 0.08 | 0.72 |  | 0.48 | 0.14 | 0.08 | 0.72 |
| Diversity language |  | 0.42 | 0.24 | 0.06 | 0.92 |  | 0.44 | 0.25 | 0.06 | 0.92 |
| Diversity residential area |  | 0.68 | 0.22 | 0.06 | 0.94 |  | 0.67 | 0.23 | 0.06 | 0.94 |
| Diversity gender |  | 0.46 | 0.07 | 0.09 | 0.5 |  | 0.46 | 0.06 | 0.09 | 0.5 |
| Abs. diff. diversity socio-economic backgr. |  |  |  |  |  |  | 0.2 | 0.12 | 0 | 0.59 |
| Abs. diff. diversity educational backgr. |  | 0.14 | 0.11 | 0 | 0.47 |  |  |  |  |  |
| Abs. diff. diversity country of origin |  | 0.23 | 0.14 | 0 | 0.78 |  | 0.26 | 0.16 | 0 | 0.71 |
| Abs. diff. diversity religion |  | 0.14 | 0.1 | 0 | 0.47 |  | 0.17 | 0.12 | 0 | 0.56 |
| Abs. diff. diversity language |  | 0.21 | 0.13 | 0 | 0.75 |  | 0.24 | 0.14 | 0 | 0.69 |
| Abs. diff. diversity residential area |  | 0.31 | 0.16 | 0 | 0.78 |  | 0.36 | 0.19 | 0 | 0.84 |
| Abs. diff. diversity gender |  | 0.08 | 0.08 | 0 | 0.41 |  | 0.11 | 0.1 | 0 | 0.43 |
| No. of categories socio-economic backgr. |  |  |  |  |  |  | 2.92 | 0.28 | 2 | 3 |
| No. of categories educational backgr. |  | 2.55 | 0.5 | 2 | 3 |  |  |  |  |  |
| No. of categories country of origin |  | 6.83 | 2.97 | 2 | 22 |  | 7 | 3 | 2 | 22 |
| No. of categories religion |  | 3.09 | 0.8 | 2 | 6 |  | 3.1 | 0.81 | 2 | 6 |
| No. of categories language |  | 4.84 | 2.41 | 2 | 17 |  | 5.03 | 2.47 | 2 | 17 |
| No. of categories residential area |  | 9.15 | 4.06 | 2 | 21 |  | 8.96 | 4.07 | 2 | 21 |
| No. of categories gender |  | 2 | 0 | 2 | 2 |  | 2 | 0 | 2 | 2 |
| Ingroup share | **Group-defining attribute: Country of origin** | 0.52 | 0.27 | 0 | 1 | **Group-defining attribute: Religion** | 0.48 | 0.25 | 0 | 1 |
| Class size |  | 21.43 | 4.37 | 10 | 32 |  | 21.43 | 4.4 | 10 | 32 |
| Group size |  | 9.83 | 6.2 | 3 | 27 |  | 9.42 | 5.2 | 3 | 26 |
| Ingr.-outgr. diversity |  | 0.36 | 0.11 | 0.08 | 0.5 |  | 0.39 | 0.1 | 0.08 | 0.5 |
| Consolidation with socio-economic backgr. |  | 0.32 | 0.16 | 0 | 0.9 |  | 0.31 | 0.15 | 0 | 0.82 |
| Consolidation with educational backgr. |  | 0.3 | 0.17 | 0 | 1 |  | 0.28 | 0.17 | 0 | 1 |
| Consolidation with country of origin |  |  |  |  |  |  | 0.69 | 0.25 | 0.05 | 1 |
| Consolidation with religion |  | 0.52 | 0.24 | 0 | 1 |  |  |  |  |  |
| Consolidation with language |  | 0.72 | 0.19 | 0.05 | 1 |  | 0.57 | 0.27 | 0 | 1 |
| Consolidation with residential area |  | 0.61 | 0.23 | 0.01 | 1 |  | 0.64 | 0.21 | 0.1 | 1 |
| Consolidation with gender |  | 0.21 | 0.15 | 0 | 0.78 |  | 0.2 | 0.15 | 0 | 0.85 |
| Diversity socio-economic background |  | 0.56 | 0.08 | 0.1 | 0.67 |  | 0.57 | 0.08 | 0.1 | 0.67 |
| Diversity educational background |  | 0.4 | 0.15 | 0.07 | 0.66 |  | 0.4 | 0.15 | 0.07 | 0.66 |
| Diversity country of origin |  |  |  |  |  |  | 0.57 | 0.22 | 0.08 | 0.95 |
| Diversity religion |  | 0.49 | 0.14 | 0.08 | 0.72 |  |  |  |  |  |
| Diversity language |  | 0.49 | 0.24 | 0.06 | 0.92 |  | 0.44 | 0.24 | 0.06 | 0.92 |
| Diversity residential area |  | 0.67 | 0.22 | 0.06 | 0.94 |  | 0.68 | 0.22 | 0.06 | 0.94 |
| Diversity gender |  | 0.46 | 0.06 | 0.09 | 0.5 |  | 0.45 | 0.07 | 0.09 | 0.5 |
| Abs. diff. diversity socio-economic backgr. |  | 0.22 | 0.13 | 0 | 0.56 |  | 0.19 | 0.11 | 0 | 0.58 |
| Abs. diff. diversity educational backgr. |  | 0.16 | 0.11 | 0 | 0.47 |  | 0.14 | 0.1 | 0 | 0.42 |
| Abs. diff. diversity country of origin |  |  |  |  |  |  | 0.25 | 0.15 | 0 | 0.7 |
| Abs. diff. diversity religion |  | 0.18 | 0.12 | 0 | 0.5 |  |  |  |  |  |
| Abs. diff. diversity language |  | 0.22 | 0.16 | 0 | 0.75 |  | 0.21 | 0.13 | 0 | 0.63 |
| Abs. diff. diversity residential area |  | 0.35 | 0.19 | 0 | 0.83 |  | 0.33 | 0.17 | 0.01 | 0.82 |
| Abs. diff. diversity gender |  | 0.13 | 0.1 | 0 | 0.42 |  | 0.1 | 0.09 | 0 | 0.42 |
| No. of categories socio-economic backgr. |  | 2.9 | 0.3 | 2 | 3 |  | 2.91 | 0.28 | 2 | 3 |
| No. of categories educational backgr. |  | 2.62 | 0.49 | 2 | 3 |  | 2.58 | 0.49 | 2 | 3 |
| No. of categories country of origin |  |  |  |  |  |  | 7.13 | 2.97 | 2 | 22 |
| No. of categories religion |  | 3.18 | 0.8 | 2 | 6 |  |  |  |  |  |
| No. of categories language |  | 5.36 | 2.48 | 2 | 17 |  | 5.05 | 2.41 | 2 | 17 |
| No. of categories residential area |  | 8.86 | 4.01 | 2 | 21 |  | 9.06 | 4.05 | 2 | 21 |
| No. of categories gender |  | 2 | 0 | 2 | 2 |  | 2 | 0 | 2 | 2 |
|  |  |  |  |  |  |  |  |  |  |  |
|  |  |  |  |  |  |  |  |  |  |  |
| Ingroup share | **Group-defining attribute: Language** | 0.58 | 0.3 | 0 | 1 | **Group-defining attribute: Residential area** | 0.42 | 0.26 | 0 | 1 |
| Class size |  | 21.21 | 4.45 | 10 | 32 |  | 21.84 | 4.39 | 10 | 32 |
| Group size |  | 11.33 | 7.08 | 3 | 29 |  | 7.1 | 4.94 | 3 | 30 |
| Ingr.-outgr. diversity |  | 0.32 | 0.13 | 0.06 | 0.5 |  | 0.35 | 0.11 | 0.06 | 0.5 |
| Consolidation with socio-economic backgr. |  | 0.33 | 0.16 | 0 | 1 |  | 0.28 | 0.14 | 0 | 0.84 |
| Consolidation with educational backgr. |  | 0.31 | 0.18 | 0 | 1 |  | 0.25 | 0.15 | 0 | 0.88 |
| Consolidation with country of origin |  | 0.86 | 0.18 | 0.05 | 1 |  | 0.51 | 0.22 | 0.04 | 1 |
| Consolidation with religion |  | 0.54 | 0.25 | 0 | 1 |  | 0.32 | 0.17 | 0 | 1 |
| Consolidation with language |  |  |  |  |  |  | 0.41 | 0.22 | 0.02 | 1 |
| Consolidation with residential area |  | 0.6 | 0.24 | 0.04 | 1 |  |  |  |  |  |
| Consolidation with gender |  | 0.21 | 0.15 | 0 | 1 |  | 0.25 | 0.17 | 0 | 1 |
| Diversity socio-economic background |  | 0.56 | 0.09 | 0.1 | 0.67 |  | 0.57 | 0.08 | 0.1 | 0.67 |
| Diversity educational background |  | 0.4 | 0.15 | 0.07 | 0.66 |  | 0.39 | 0.15 | 0.07 | 0.66 |
| Diversity country of origin |  | 0.61 | 0.22 | 0.08 | 0.95 |  | 0.54 | 0.22 | 0.08 | 0.95 |
| Diversity religion |  | 0.48 | 0.14 | 0.08 | 0.72 |  | 0.47 | 0.14 | 0.08 | 0.72 |
| Diversity language |  |  |  |  |  |  | 0.41 | 0.24 | 0.06 | 0.92 |
| Diversity residential area |  | 0.67 | 0.23 | 0.06 | 0.94 |  |  |  |  |  |
| Diversity gender |  | 0.46 | 0.07 | 0.09 | 0.5 |  | 0.46 | 0.06 | 0.09 | 0.5 |
| Abs. diff. diversity socio-economic backgr. |  | 0.25 | 0.14 | 0 | 0.57 |  | 0.23 | 0.12 | 0 | 0.56 |
| Abs. diff. diversity educational backgr. |  | 0.17 | 0.11 | 0 | 0.52 |  | 0.15 | 0.11 | 0 | 0.53 |
| Abs. diff. diversity country of origin |  | 0.29 | 0.15 | 0 | 0.78 |  | 0.26 | 0.17 | 0 | 0.83 |
| Abs. diff. diversity religion |  | 0.19 | 0.12 | 0 | 0.52 |  | 0.18 | 0.12 | 0 | 0.53 |
| Abs. diff. diversity language |  |  |  |  |  |  | 0.22 | 0.14 | 0 | 0.8 |
| Abs. diff. diversity residential area |  | 0.38 | 0.21 | 0 | 0.84 |  |  |  |  |  |
| Abs. diff. diversity gender |  | 0.15 | 0.12 | 0 | 0.42 |  | 0.14 | 0.1 | 0 | 0.42 |
| No. of categories socio-economic backgr. |  | 2.88 | 0.32 | 2 | 3 |  | 2.91 | 0.28 | 2 | 3 |
| No. of categories educational backgr. |  | 2.62 | 0.49 | 2 | 3 |  | 2.54 | 0.5 | 2 | 3 |
| No. of categories country of origin |  | 7.19 | 3.07 | 2 | 22 |  | 6.71 | 2.84 | 2 | 22 |
| No. of categories religion |  | 3.13 | 0.8 | 2 | 6 |  | 3.09 | 0.79 | 2 | 6 |
| No. of categories language |  |  |  |  |  |  | 4.77 | 2.31 | 2 | 17 |
| No. of categories residential area |  | 8.76 | 4.03 | 2 | 21 |  |  |  |  |  |
| No. of categories gender |  | 2 | 0 | 2 | 2 |  | 2 | 0 | 2 | 2 |
| Ingroup share | **Group-defining attribute: Gender** | 0.86 | 0.13 | 0.15 | 1 |  |  |  |  |  |
| Class size |  | 21.28 | 4.37 | 10 | 32 |  |  |  |  |  |
| Group size |  | 10.72 | 3.65 | 3 | 24 |  |  |  |  |  |
| Ingr.-outgr. diversity |  | 0.46 | 0.06 | 0.09 | 0.5 |  |  |  |  |  |
| Consolidation with socio-economic backgr. |  | 0.28 | 0.14 | 0 | 0.78 |  |  |  |  |  |
| Consolidation with educational backgr. |  | 0.25 | 0.14 | 0 | 1 |  |  |  |  |  |
| Consolidation with country of origin |  | 0.53 | 0.17 | 0.03 | 1 |  |  |  |  |  |
| Consolidation with religion |  | 0.31 | 0.15 | 0 | 0.88 |  |  |  |  |  |
| Consolidation with language |  | 0.43 | 0.18 | 0.01 | 1 |  |  |  |  |  |
| Consolidation with residential area |  | 0.67 | 0.2 | 0.11 | 1 |  |  |  |  |  |
| Consolidation with gender |  |  |  |  |  |  |  |  |  |  |
| Diversity socio-economic background |  | 0.57 | 0.08 | 0.1 | 0.67 |  |  |  |  |  |
| Diversity educational background |  | 0.4 | 0.15 | 0.07 | 0.66 |  |  |  |  |  |
| Diversity country of origin |  | 0.55 | 0.22 | 0.08 | 0.95 |  |  |  |  |  |
| Diversity religion |  | 0.48 | 0.14 | 0.08 | 0.72 |  |  |  |  |  |
| Diversity language |  | 0.43 | 0.24 | 0.06 | 0.92 |  |  |  |  |  |
| Diversity residential area |  | 0.68 | 0.22 | 0.06 | 0.94 |  |  |  |  |  |
| Diversity gender |  |  |  |  |  |  |  |  |  |  |
| Abs. diff. diversity socio-economic backgr. |  | 0.13 | 0.08 | 0 | 0.53 |  |  |  |  |  |
| Abs. diff. diversity educational backgr. |  | 0.13 | 0.11 | 0 | 0.46 |  |  |  |  |  |
| Abs. diff. diversity country of origin |  | 0.21 | 0.13 | 0 | 0.66 |  |  |  |  |  |
| Abs. diff. diversity religion |  | 0.13 | 0.1 | 0 | 0.51 |  |  |  |  |  |
| Abs. diff. diversity language |  | 0.22 | 0.12 | 0 | 0.57 |  |  |  |  |  |
| Abs. diff. diversity residential area |  | 0.29 | 0.14 | 0 | 0.76 |  |  |  |  |  |
| Abs. diff. diversity gender |  |  |  |  |  |  |  |  |  |  |
| No. of categories socio-economic backgr. |  | 2.9 | 0.3 | 2 | 3 |  |  |  |  |  |
| No. of categories educational backgr. |  | 2.55 | 0.5 | 2 | 3 |  |  |  |  |  |
| No. of categories country of origin |  | 6.8 | 2.91 | 2 | 22 |  |  |  |  |  |
| No. of categories religion |  | 3.09 | 0.8 | 2 | 6 |  |  |  |  |  |
| No. of categories language |  | 4.86 | 2.38 | 2 | 17 |  |  |  |  |  |
| No. of categories residential area |  | 9.08 | 4.03 | 2 | 21 |  |  |  |  |  |
| No. of categories gender |  |  |  |  |  |  |  |  |  |  |
| Summary statistics of the variables used in the main models of Study 1, based on the first imputed dataset. Information on the other imputed datasets is available on request from the authors. Ingr.-outgr. diversity = Ingroup-outgroup diversity; Abs. diff. = Absolute difference | | | | | | | | | | |
