# Supplementary material for: Friendship segregation and class composition in schools: A systematic analysis of the role of attribute consolidation
Source: PLoS One. 2025 Dec 31;20(12):e0339581. doi: 10.1371/journal.pone.0339581 (PMC12755804; doi:10.1371/journal.pone.0339581)
Supplement: S7 Table — (DOCX) [file pone.0339581.s015.docx]

**Table S7:** OLS models regressing the share of ingroup friends on consolidation with school fixed effects

|  |  | **Consolidating attribute** | | | | | | | |
| --- | --- | --- | --- | --- | --- | --- | --- | --- | --- |
|  | **Variable** | **Socio-econ. backgr.** | **Educat. backgr.** | **Country of origin** | **Religion** | **Language** | **Resident. area** | **Gender** | |
| **Group-defining attribute:  Socio-economic background** |  |  |  |  |  |  |  |  | |
|  | Intercept |  | 0.301*** | 0.307*** | 0.309*** | 0.301*** | 0.23** | 0.211* | |
|  |  |  | (4.98) | (5.22) | (4.59) | (5.14) | (3.2) | (2.38) | |
|  | Consolidation |  | 0.026 | 0.09* | 0.043 | 0.087* | 0.066 | 0.237*** | |
|  |  |  | (1) | (2.47) | (1.39) | (2.13) | (1.55) | (7.42) | |
|  | Class size |  | -0.015*** | -0.013*** | -0.014*** | -0.013*** | -0.013*** | -0.013*** | |
|  |  |  | (-8.29) | (-6.93) | (-7.99) | (-6.92) | (-6.5) | (-7.39) | |
|  | Group size |  | 0.048*** | 0.048*** | 0.048*** | 0.047*** | 0.048*** | 0.047*** | |
|  |  |  | (27.74) | (27.41) | (27.79) | (27.32) | (28.11) | (27.78) | |
|  | Ingr.-outgr. diversity |  | 0.025 | 0.029 | 0.024 | 0.029 | 0 | 0.127 | |
|  |  |  | (0.34) | (0.39) | (0.31) | (0.39) | (0) | (1.22) | |
|  | Diversity cons. attr. |  | -0.047 | -0.055 | -0.01 | -0.01 | 0.094* | 0.001 | |
|  |  |  | (-0.7) | (-0.85) | (-0.16) | (-0.14) | (1.96) | (0.01) | |
|  | Abs. diff. diversity |  | -0.009 | -0.014 | 0.019 | 0.012 | -0.017 | 0.137 | |
|  |  |  | (-0.16) | (-0.29) | (0.35) | (0.24) | (-0.35) | (1.38) | |
|  | Number of categories |  | 0.009 | -0.007 | -0.009 | -0.01 | -0.005 |  | |
|  |  |  | (0.75) | (-1.52) | (-1.08) | (-1.59) | (-1.46) |  | |
| **Group-defining attribute:  Educational background** |  |  |  |  |  |  |  |  | |
|  | Intercept | 0.67*** |  | 0.64*** | 0.686*** | 0.682*** | 0.714*** | 0.63*** | |
|  |  | (6.88) |  | (10.62) | (16.07) | (14.8) | (12.48) | (6.11) | |
|  | Consolidation | 0.074* |  | 0.102* | 0.08* | 0.068 | 0.101* | 0.257*** | |
|  |  | (2.16) |  | (2.32) | (2.16) | (1.74) | (2.25) | (6.25) | |
|  | Class size | -0.022*** |  | -0.021*** | -0.021*** | -0.021*** | -0.021*** | -0.021*** | |
|  |  | (-12.32) |  | (-10.85) | (-10.53) | (-10.85) | (-9.91) | (-12.33) | |
|  | Group size | 0.044*** |  | 0.044*** | 0.044*** | 0.044*** | 0.044*** | 0.044*** | |
|  |  | (46.62) |  | (47.25) | (46.58) | (46.25) | (46.26) | (46.48) | |
|  | Ingr.-outgr. diversity | 0.138 |  | -0.086 | -0.01 | -0.04 | -0.11 | -0.066 | |
|  |  | (0.73) |  | (-0.99) | (-0.12) | (-0.51) | (-1.11) | (-0.44) | |
|  | Diversity cons. attr. | -0.163 |  | -0.041 | -0.042 | -0.051 | -0.073 | 0.089 | |
|  |  | (-1.07) |  | (-0.54) | (-0.66) | (-0.7) | (-1.48) | (0.78) | |
|  | Abs. diff. diversity | 0.248 |  | -0.04 | 0.089 | 0.037 | -0.017 | 0.005 | |
|  |  | (1.32) |  | (-0.62) | (1.26) | (0.68) | (-0.3) | (0.03) | |
|  | Number of categories | -0.013 |  | 0 | -0.009 | -0.005 | -0.001 |  | |
|  |  | (-0.46) |  | (-0.09) | (-0.98) | (-0.78) | (-0.22) |  | |
| **Group-defining attribute:  Country of origin** |  |  |  |  |  |  |  |  | |
|  | Intercept | 0.518*** | 0.476*** |  | 0.523*** | 0.405*** | 0.354*** | 0.388*** | |
|  |  | (3.62) | (6.89) |  | (7.62) | (5.56) | (4.51) | (3.79) | |
|  | Consolidation | 0.075 | 0.085 |  | 0.197*** | 0.201*** | 0.123* | 0.334*** | |
|  |  | (1.31) | (1.52) |  | (5.17) | (3.92) | (2.34) | (6.59) | |
|  | Class size | -0.014*** | -0.013*** |  | -0.012*** | -0.012*** | -0.011*** | -0.015*** | |
|  |  | (-4.22) | (-4.01) |  | (-4.08) | (-3.71) | (-3.48) | (-5.07) | |
|  | Group size | 0.037*** | 0.037*** |  | 0.036*** | 0.034*** | 0.037*** | 0.037*** | |
|  |  | (20.46) | (20.26) |  | (20.63) | (16.72) | (21.06) | (21.34) | |
|  | Ingr.-outgr. diversity | 0.385* | 0.216** |  | 0.151 | 0.223** | 0.29** | 0.103 | |
|  |  | (2.07) | (2.63) |  | (1.6) | (2.63) | (2.63) | (0.79) | |
|  | Diversity cons. attr. | -0.054 | -0.145 |  | -0.19* | -0.148 | 0.072 | 0.188 | |
|  |  | (-0.3) | (-1.32) |  | (-2.05) | (-1.18) | (0.84) | (1.08) | |
|  | Abs. diff. diversity | 0.205 | 0.048 |  | -0.025 | -0.21** | 0.16 | -0.14 | |
|  |  | (1.13) | (0.6) |  | (-0.26) | (-2.63) | (1.73) | (-1.08) | |
|  | Number of categories | -0.034 | -0.008 |  | -0.006 | -0.004 | -0.012** |  | |
|  |  | (-0.71) | (-0.35) |  | (-0.47) | (-0.46) | (-2.66) |  | |
| **Group-defining attribute:  Religion** |  |  |  |  |  |  |  |  | |
|  | Intercept | 0.575*** | 0.555*** | 0.572*** |  | 0.57*** | 0.417*** | 0.324** | |
|  |  | (4.91) | (11.53) | (12.17) |  | (11.81) | (6.57) | (3.18) | |
|  | Consolidation | 0.126** | 0.097* | 0.236*** |  | 0.192*** | 0.188*** | 0.305*** | |
|  |  | (2.63) | (2.02) | (5.63) |  | (5.29) | (3.37) | (7.34) | |
|  | Class size | -0.015*** | -0.015*** | -0.014*** |  | -0.014*** | -0.012*** | -0.016*** | |
|  |  | (-6.18) | (-5.65) | (-5.98) |  | (-5.6) | (-4.64) | (-6.78) | |
|  | Group size | 0.042*** | 0.042*** | 0.042*** |  | 0.042*** | 0.042*** | 0.043*** | |
|  |  | (40.33) | (41.04) | (39.47) |  | (38.9) | (40.52) | (41.91) | |
|  | Ingr.-outgr. diversity | 0.12 | 0.085 | 0.13 |  | 0.091 | 0.128 | 0.172 | |
|  |  | (0.72) | (1.1) | (1.67) |  | (1.22) | (1.38) | (1.61) | |
|  | Diversity cons. attr. | -0.076 | 0.045 | -0.144 |  | -0.019 | 0.089 | 0.189 | |
|  |  | (-0.65) | (0.55) | (-1.92) |  | (-0.21) | (1.47) | (1.42) | |
|  | Abs. diff. diversity | 0.042 | 0.022 | 0.012 |  | -0.021 | 0.038 | 0.121 | |
|  |  | (0.27) | (0.29) | (0.2) |  | (-0.33) | (0.6) | (1.14) | |
|  | Number of categories | -0.014 | -0.022 | -0.007 |  | -0.014* | -0.013** |  | |
|  |  | (-0.4) | (-1.25) | (-1.57) |  | (-2.36) | (-3.14) |  | |
| **Group-defining attribute:  Language** |  |  |  |  |  |  |  |  | |
|  | Intercept | 0.57*** | 0.657*** | 0.667*** | 0.653*** |  | 0.607*** | 0.395*** | |
|  |  | (4.02) | (10.79) | (9.03) | (10.6) |  | (9.15) | (4) | |
|  | Consolidation | 0.063 | 0.009 | 0.115* | 0.091* |  | 0.056 | 0.296*** | |
|  |  | (1.24) | (0.2) | (2.22) | (2.32) |  | (1.2) | (5.46) | |
|  | Class size | -0.021*** | -0.02*** | -0.02*** | -0.021*** |  | -0.021*** | -0.019*** | |
|  |  | (-6.54) | (-6.37) | (-6.25) | (-6.56) |  | (-6.15) | (-6.7) | |
|  | Group size | 0.039*** | 0.038*** | 0.036*** | 0.038*** |  | 0.039*** | 0.038*** | |
|  |  | (23.36) | (22.66) | (20.54) | (22.82) |  | (23.72) | (25.13) | |
|  | Ingr.-outgr. diversity | 0.36 | 0.23* | -0.058 | 0.176 |  | 0.226* | 0.238 | |
|  |  | (1.71) | (2.51) | (-0.19) | (1.64) |  | (2.4) | (1.81) | |
|  | Diversity cons. attr. | -0.055 | -0.068 | 0.037 | -0.076 |  | -0.034 | 0.112 | |
|  |  | (-0.27) | (-0.75) | (0.13) | (-0.8) |  | (-0.39) | (0.64) | |
|  | Abs. diff. diversity | 0.22 | 0.118 | -0.345 | 0.042 |  | 0.105 | 0.067 | |
|  |  | (1.01) | (1.22) | (-1.1) | (0.38) |  | (1.38) | (0.48) | |
|  | Number of categories | -0.005 | -0.014 | -0.003 | -0.005 |  | -0.003 |  | |
|  |  | (-0.12) | (-0.71) | (-0.67) | (-0.38) |  | (-0.66) |  | |
| **Group-defining attribute:  Residential area** |  |  |  |  |  |  |  |  | |
|  | Intercept | 0.343*** | 0.37*** | 0.317*** | 0.289*** | 0.316*** |  | 0.284** | |
|  |  | (3.32) | (4.89) | (4.15) | (3.87) | (4.67) |  | (2.83) | |
|  | Consolidation | -0.036 | 0.076 | 0.047 | 0.078 | 0.045 |  | 0.405*** | |
|  |  | (-0.65) | (1.23) | (0.84) | (1.5) | (0.83) |  | (10.06) | |
|  | Class size | -0.011*** | -0.009* | -0.01** | -0.01** | -0.01** |  | -0.01*** | |
|  |  | (-3.35) | (-2.44) | (-2.84) | (-3.21) | (-2.86) |  | (-3.6) | |
|  | Group size | 0.036*** | 0.037*** | 0.036*** | 0.037*** | 0.036*** |  | 0.039*** | |
|  |  | (21.28) | (20.54) | (21.17) | (21.49) | (20.9) |  | (24.13) | |
|  | Ingr.-outgr. diversity | 0.283 | 0.138 | 0.165* | 0.179* | 0.149* |  | 0.179 | |
|  |  | (1.45) | (1.86) | (2.11) | (2.18) | (2.13) |  | (1.56) | |
|  | Diversity cons. attr. | -0.176 | -0.103 | -0.051 | 0.064 | 0 |  | -0.378** | |
|  |  | (-0.98) | (-1.14) | (-0.43) | (0.78) | (0) |  | (-2.67) | |
|  | Abs. diff. diversity | 0.141 | -0.006 | 0.038 | 0.084 | 0.035 |  | 0.132 | |
|  |  | (0.71) | (-0.08) | (0.61) | (1.16) | (0.53) |  | (1.13) | |
|  | Number of categories | 0.006 | -0.037 | -0.003 | -0.018 | -0.006 |  |  | |
|  |  | (0.22) | (-1.66) | (-0.4) | (-1.29) | (-0.74) |  |  | |
| **Group-defining attribute:  Gender** |  |  |  |  |  |  |  |  | |
|  | Intercept | 0.508*** | 0.599*** | 0.659*** | 0.701*** | 0.572*** | 0.56*** |  | |
|  |  | (3.91) | (6.77) | (8.07) | (7.46) | (6.9) | (5.79) |  | |
|  | Consolidation | -0.005 | 0.038 | -0.148** | -0.008 | -0.02 | 0.062 |  | |
|  |  | (-0.1) | (0.79) | (-2.62) | (-0.2) | (-0.31) | (0.94) |  | |
|  | Class size | -0.005* | -0.004* | -0.005* | -0.004* | -0.005* | -0.003 |  | |
|  |  | (-2.57) | (-2.11) | (-2.53) | (-2.11) | (-2.33) | (-1.14) |  | |
|  | Group size | 0.018*** | 0.018*** | 0.018*** | 0.018*** | 0.018*** | 0.018*** |  | |
|  |  | (11.2) | (11.2) | (11.22) | (11.2) | (11.21) | (11.18) |  | |
|  | Ingr.-outgr. diversity | 0.273 | 0.256 | 0.291* | 0.228 | 0.265 | 0.278 |  | |
|  |  | (1.47) | (1.84) | (2.04) | (1.57) | (1.85) | (1.93) |  | |
|  | Diversity cons. attr. | -0.072 | 0.176* | 0.021 | 0.029 | 0.131 | 0.037 |  | |
|  |  | (-0.8) | (2.03) | (0.35) | (0.38) | (1.62) | (0.53) |  | |
|  | Abs. diff. diversity | 0.003 | 0.094 | 0.003 | -0.08 | 0.122 | 0.038 |  | |
|  |  | (0.03) | (1.06) | (0.06) | (-1.05) | (1.67) | (0.67) |  | |
|  | Number of categories | 0.058 | -0.018 | 0.003 | -0.015 | 0.002 | -0.007 |  | |
|  |  | (1.73) | (-1.1) | (0.66) | (-1.44) | (0.37) | (-1.64) |  | |
| Unstandardized coefficients and t-values in parentheses of OLS regressions with cluster robust standard errors and school fixed effects. Pooled results over ten imputations using Rubin’s rules. ***p<0.001 **p<0.01 *p<0.05. Ingr.-outgr. Diversity = Ingroup-outgroup diversity; Diversity cons. attr. = Diversity of the consolidating attribute; Abs. diff. diversity = Absolute difference between ingroup-outgroup diversity and diversity in the consolidating attribute. | | | | | | | | |  |
