# Supplementary material for: Friendship segregation and class composition in schools: A systematic analysis of the role of attribute consolidation
Source: PLoS One. 2025 Dec 31;20(12):e0339581. doi: 10.1371/journal.pone.0339581 (PMC12755804; doi:10.1371/journal.pone.0339581)
Supplement: S8 Table — (DOCX) [file pone.0339581.s016.docx]

**Table S8:** OLS models regressing consolidation on structural variables

|  |  | **Consolidating attribute** | | | | | | |
| --- | --- | --- | --- | --- | --- | --- | --- | --- |
|  | **Variable** | **Socio-econ. backgr.** | **Educat. backgr.** | **Country of origin** | **Religion** | **Language** | **Resident. area** | **Gender** |
| **Group-defining attribute:  Socio-econ. background** | Class size |  | -0.05 | -0.274*** | -0.213*** | -0.247*** | -0.29*** | -0.137** |
|  |  |  | (-1.21) | (-8.78) | (-5.39) | (-8.53) | (-9.74) | (-3.21) |
|  | Group size |  | 0.044 | 0.047 | 0.032 | 0.049 | -0.005 | -0.011 |
|  |  |  | (0.91) | (1.43) | (0.68) | (1.34) | (-0.18) | (-0.26) |
|  | Ingr.-outgr. diversity |  | -0.101 | -0.132** | -0.004 | -0.06 | -0.006 | 0.015 |
|  |  |  | (-1.84) | (-3.2) | (-0.08) | (-1.38) | (-0.15) | (0.27) |
|  | Diversity cons. attr. |  | 0.147*** | 0.3*** | -0.018 | 0.157** | 0.256*** | 0.014 |
|  |  |  | (3.38) | (5.73) | (-0.5) | (2.9) | (6.43) | (0.33) |
|  | Abs. diff. diversity |  | -0.133*** | -0.104*** | 0.01 | -0.123*** | -0.051 | 0 |
|  |  |  | (-3.63) | (-4.48) | (0.33) | (-5.24) | (-1.62) | (0.01) |
|  | No. of categories |  | 0.151*** | 0.538*** | 0.444*** | 0.613*** | 0.685*** |  |
|  |  |  | (3.91) | (9.6) | (11.04) | (10.9) | (14.65) |  |
| **Group-defining attribute:**  **Educational background** | Class size | -0.028 |  | -0.255*** | -0.255*** | -0.244*** | -0.261*** | -0.196*** |
|  |  | (-0.6) |  | (-6.31) | (-6.68) | (-6.63) | (-8.71) | (-4.56) |
|  | Group size | 0.008 |  | 0.019 | 0.043 | 0.047* | 0.011 | 0.022 |
|  |  | (0.36) |  | (0.82) | (1.74) | (2.07) | (0.57) | (0.95) |
|  | Ingr.-outgr. diversity | 0.015 |  | -0.179*** | -0.097* | -0.141*** | 0.1** | 0.04 |
|  |  | (0.12) |  | (-4.3) | (-2.1) | (-3.94) | (2.67) | (0.49) |
|  | Diversity cons. attr. | 0.133 |  | 0.312*** | 0.092* | 0.183** | 0.306*** | 0.076 |
|  |  | (1.79) |  | (5.03) | (2.3) | (2.7) | (6.85) | (1.87) |
|  | Abs. diff. diversity | -0.148 |  | -0.094* | 0.023 | -0.032 | -0.018 | 0.11 |
|  |  | (-1.12) |  | (-2.27) | (0.51) | (-1.06) | (-0.44) | (1.37) |
|  | No. of categories | 0.212** |  | 0.455*** | 0.36*** | 0.511*** | 0.585*** |  |
|  |  | (3.28) |  | (6.91) | (7.79) | (7.06) | (10.61) |  |
| **Group-defining attribute:  Country of origin** | Class size | -0.121* | -0.247*** |  | -0.167*** | -0.14** | -0.163*** | -0.205*** |
|  |  | (-2.57) | (-5.88) |  | (-4.22) | (-3.09) | (-4.94) | (-5.07) |
|  | Group size | -0.022 | 0.078* |  | 0.069 | 0.187* | -0.013 | -0.02 |
|  |  | (-0.6) | (2.06) |  | (1.71) | (2.41) | (-0.44) | (-0.6) |
|  | Ingr.-outgr. diversity | 0.03 | -0.024 |  | 0.065 | -0.381*** | 0.078* | -0.023 |
|  |  | (0.25) | (-0.61) |  | (1.48) | (-7.45) | (2.17) | (-0.35) |
|  | Diversity cons. attr. | 0.012 | -0.047 |  | 0.317*** | 0.877*** | 0.439*** | -0.009 |
|  |  | (0.15) | (-1.1) |  | (8.28) | (8.57) | (10.5) | (-0.23) |
|  | Abs. diff. diversity | 0.003 | -0.016 |  | -0.201*** | -0.569*** | -0.035 | -0.025 |
|  |  | (0.02) | (-0.47) |  | (-4.57) | (-11.31) | (-0.82) | (-0.39) |
|  | No. of categories | 0.199*** | 0.436*** |  | 0.199*** | 0.083 | 0.419*** |  |
|  |  | (3.77) | (9.56) |  | (4.3) | (0.95) | (8.02) |  |
| **Group-defining attribute:  Religion** | Class size | -0.152*** | -0.241*** | -0.095** |  | -0.102** | -0.222*** | -0.126** |
|  |  | (-3.5) | (-6.31) | (-2.74) |  | (-3.18) | (-6.97) | (-3.06) |
|  | Group size | 0.019 | 0.031 | 0.083** |  | 0.084*** | -0.025 | 0.012 |
|  |  | (0.75) | (1.1) | (3.21) |  | (3.5) | (-1.05) | (0.47) |
|  | Ingr.-outgr. diversity | 0.024 | -0.029 | -0.152*** |  | -0.095** | -0.036 | 0.056 |
|  |  | (0.18) | (-0.79) | (-4.34) |  | (-2.89) | (-0.98) | (0.96) |
|  | Diversity cons. attr. | -0.023 | -0.11** | 0.855*** |  | 0.662*** | 0.387*** | 0.028 |
|  |  | (-0.29) | (-2.77) | (16.11) |  | (11.64) | (8.96) | (0.79) |
|  | Abs. diff. diversity | 0.053 | -0.059 | -0.11*** |  | -0.218*** | -0.109** | 0.09 |
|  |  | (0.37) | (-1.81) | (-3.35) |  | (-8.77) | (-2.87) | (1.62) |
|  | No. of categories | 0.128* | 0.427*** | -0.091 |  | 0.113* | 0.556*** |  |
|  |  | (2.21) | (10.5) | (-1.58) |  | (1.99) | (10.43) |  |
| **Group-defining attribute: Language** | Class size | -0.126** | -0.23*** | -0.205*** | -0.16*** |  | -0.133*** | -0.201*** |
|  |  | (-2.93) | (-5.32) | (-4.5) | (-3.86) |  | (-3.53) | (-4.75) |
|  | Group size | 0.104** | 0.082* | 0.256*** | 0.206*** |  | 0.041 | -0.015 |
|  |  | (2.77) | (2.27) | (4.1) | (5.4) |  | (1.4) | (-0.45) |
|  | Ingr.-outgr. diversity | -0.025 | -0.118** | -0.758*** | -0.027 |  | 0.091* | 0.05 |
|  |  | (-0.23) | (-2.71) | (-7.39) | (-0.59) |  | (2.42) | (0.57) |
|  | Diversity cons. attr. | 0.058 | -0.125** | 1.076*** | 0.321*** |  | 0.475*** | 0.008 |
|  |  | (1.04) | (-2.87) | (5.72) | (8.41) |  | (9.71) | (0.19) |
|  | Abs. diff. diversity | -0.073 | -0.031 | -0.892*** | -0.207*** |  | -0.01 | 0.075 |
|  |  | (-0.66) | (-0.79) | (-6.81) | (-4.47) |  | (-0.21) | (0.91) |
|  | No. of categories | 0.216*** | 0.48*** | 0.35*** | 0.255*** |  | 0.3*** |  |
|  |  | (3.68) | (10.09) | (4.47) | (5.16) |  | (4.93) |  |
|  |  |  |  |  |  |  |  |  |
|  |  |  |  |  |  |  |  |  |
|  |  |  |  |  |  |  |  |  |
|  |  |  |  |  |  |  |  |  |
| **Group-defining attribute: Residential area** | Class size | -0.196*** | -0.181*** | -0.222*** | -0.163*** | -0.177*** |  | -0.123*** |
|  |  | (-4.61) | (-4.95) | (-7.49) | (-4.55) | (-5.81) |  | (-3.29) |
|  | Group size | 0.049 | -0.007 | -0.002 | -0.027 | -0.012 |  | -0.079** |
|  |  | (1.47) | (-0.21) | (-0.05) | (-0.83) | (-0.35) |  | (-2.92) |
|  | Ingr.-outgr. diversity | 0.043 | 0.014 | 0.069* | 0.131*** | 0.094** |  | 0.092 |
|  |  | (0.36) | (0.38) | (2.21) | (3.52) | (2.76) |  | (1.73) |
|  | Diversity cons. attr. | -0.036 | 0.027 | 0.339*** | 0.158*** | 0.25*** |  | 0.075** |
|  |  | (-0.46) | (0.77) | (7.2) | (4.84) | (5.1) |  | (2.67) |
|  | Abs. diff. diversity | 0.078 | -0.022 | -0.058 | 0.062 | -0.059* |  | 0.015 |
|  |  | (0.57) | (-0.71) | (-1.94) | (1.85) | (-2.25) |  | (0.3) |
|  | No. of categories | 0.187*** | 0.341*** | 0.423*** | 0.323*** | 0.487*** |  |  |
|  |  | (3.5) | (9.42) | (8.58) | (9.48) | (9.52) |  |  |
| **Group-defining attribute: Gender** | Class size | -0.227*** | -0.237*** | -0.299*** | -0.186*** | -0.248*** | -0.244*** |  |
|  |  | (-4.69) | (-5.48) | (-8.94) | (-4.2) | (-7.27) | (-8.29) |  |
|  | Group size | 0 | 0.01 | -0.021* | 0 | -0.001 | -0.005 |  |
|  |  | (0.04) | (1.07) | (-2.1) | (-0.07) | (-0.11) | (-0.5) |  |
|  | Ingr.-outgr. diversity | 0.028 | 0.12** | 0.068 | 0.111** | 0.084* | -0.037 |  |
|  |  | (0.38) | (3.04) | (1.6) | (2.73) | (2.11) | (-0.99) |  |
|  | Diversity cons. attr. | 0.048 | -0.091 | 0.266*** | 0.07 | 0.234*** | 0.534*** |  |
|  |  | (0.8) | (-1.34) | (4.43) | (1.38) | (3.5) | (11.75) |  |
|  | Abs. diff. diversity | -0.068 | -0.032 | -0.066* | 0.067 | -0.001 | -0.106** |  |
|  |  | (-0.79) | (-0.57) | (-2.17) | (1.47) | (-0.02) | (-3.23) |  |
|  | No. of categories | 0.248*** | 0.411*** | 0.575*** | 0.405*** | 0.579*** | 0.443*** |  |
|  |  | (4.54) | (8.98) | (8.89) | (8.62) | (8.84) | (7.81) |  |
| Standardized coefficients and t-values in parentheses of OLS regressions with cluster robust standard errors. Pooled results over ten imputations using Rubin’s rules. ***p<0.001 **p<0.01 *p<0.05. Ingr.-outgr. diversity = Ingroup-outgroup diversity; Diversity cons. attr. = Diversity of the consolidating attribute; Abs. diff. diversity = Absolute difference between ingroup-outgroup diversity and diversity in the consolidating attribute; No. of categories = Number of categories in the consolidating attribute. | | | | | | | | |
