# Supplementary material for: Friendship segregation and class composition in schools: A systematic analysis of the role of attribute consolidation
Source: PLoS One. 2025 Dec 31;20(12):e0339581. doi: 10.1371/journal.pone.0339581 (PMC12755804; doi:10.1371/journal.pone.0339581)
Supplement: S10 Table — (DOCX) [file pone.0339581.s018.docx]

Table S10: Significance of consolidation estimates with Bonferroni correction

|  | **Consolidating attribute** | | | | | | |
| --- | --- | --- | --- | --- | --- | --- | --- |
| **Group-defining attribute** | **Socio-economic background** | **Educational background** | **Country of origin** | **Religion** | **Language** | **Residential area** | **Gender** |
| Socio-economic background |  | 0.02629 | 0.08103 | 0.05883 | 0.07197 | 0.07577 | 0.22727*** |
|  |  | (0.21452) | (0.01041) | (0.02939) | (0.05468) | (0.03836) | (0) |
| Educational background | 0.05332 |  | 0.08916* | 0.05998 | 0.04575 | 0.04946 | 0.25329*** |
|  | (0.01588) |  | (0.00097) | (0.01839) | (0.10734) | (0.0809) | (0) |
| Country of origin | 0.08603 | 0.06351 |  | 0.14318*** | 0.16363*** | 0.09137 | 0.24804*** |
|  | (0.01035) | (0.06143) |  | (0) | (1e-05) | (0.01281) | (0) |
| Religion | 0.08018 | 0.04196 | 0.13322*** |  | 0.11181** | 0.16088** | 0.26951*** |
|  | (0.01346) | (0.1305) | (1e-05) |  | (4e-05) | (7e-05) | (0) |
| Language | 0.06443 | 0.03294 | 0.11972* | 0.08591* |  | 0.04004 | 0.24841*** |
|  | (0.06662) | (0.26786) | (0.00105) | (0.00039) |  | (0.14789) | (0) |
| Residential area | 0.00968 | 0.06324 | 0.06164 | 0.09612 | 0.0867 |  | 0.41587*** |
|  | (0.8231) | (0.17142) | (0.1171) | (0.01244) | (0.02236) |  | (0) |
| Gender | 0.02549 | 0.01056 | -0.09193 | 0.05035 | -0.00276 | 0.08979 |  |
|  | (0.47037) | (0.75097) | (0.03565) | (0.12343) | (0.95118) | (0.02343) |  |
| Unstandardized coefficients and p-values in parentheses of OLS regressions with cluster robust standard errors and groups-in-survey-countries fixed effects. Pooled results over ten imputations using Rubin’s rules. Stars show significance levels with Bonferroni correction: ***p<0.001/42 **p<0.01/42 *p<0.05/42. | | | | | | | |
