# Supplementary material for: Friendship segregation and class composition in schools: A systematic analysis of the role of attribute consolidation
Source: PLoS One. 2025 Dec 31;20(12):e0339581. doi: 10.1371/journal.pone.0339581 (PMC12755804; doi:10.1371/journal.pone.0339581)
Supplement: S11 Table — (DOCX) [file pone.0339581.s019.docx]

**Table S11:** Difference between the coefficients of gender consolidation and the other types of consolidation

| **Group-defining attribute** | **Consolidating attribute** | **Difference to gender consolidation coefficient** | **Std. Err. of difference** |
| --- | --- | --- | --- |
| Socio-economic background | Educational background | 0.20 | 0.03 |
|  | Country of origin | 0.15 | 0.04 |
|  | Religion | 0.17 | 0.04 |
|  | Language | 0.16 | 0.05 |
|  | Residential area | 0.15 | 0.05 |
| Educational background | Socio-economic background | 0.20 | 0.04 |
|  | Country of origin | 0.16 | 0.04 |
|  | Religion | 0.19 | 0.04 |
|  | Language | 0.21 | 0.04 |
|  | Residential area | 0.20 | 0.04 |
| Country of origin | Socio-economic background | 0.16 | 0.05 |
|  | Educational background | 0.18 | 0.05 |
|  | Religion | 0.10 | 0.04 |
|  | Language | 0.08 | 0.05 |
|  | Residential area | 0.16 | 0.05 |
| Religion | Socio-economic background | 0.19 | 0.05 |
|  | Educational background | 0.23 | 0.04 |
|  | Country of origin | 0.14 | 0.05 |
|  | Language | 0.16 | 0.04 |
|  | Residential area | 0.11 | 0.05 |
| Language | Socio-economic background | 0.18 | 0.05 |
|  | Educational background | 0.22 | 0.05 |
|  | Country of origin | 0.13 | 0.05 |
|  | Religion | 0.16 | 0.04 |
|  | Residential area | 0.21 | 0.04 |
| Residential area | Socio-economic background | 0.41 | 0.05 |
|  | Educational background | 0.35 | 0.05 |
|  | Country of origin | 0.35 | 0.05 |
|  | Religion | 0.32 | 0.05 |
|  | Language | 0.33 | 0.05 |
| Difference between the coefficients of gender consolidation and six other types of consolidation. Standard errors of the difference are estimated following Paternoster et al. (1998). | | | |
