# Supplementary material for: Friendship segregation and class composition in schools: A systematic analysis of the role of attribute consolidation
Source: PLoS One. 2025 Dec 31;20(12):e0339581. doi: 10.1371/journal.pone.0339581 (PMC12755804; doi:10.1371/journal.pone.0339581)
Supplement: S12 Table — Summary statistics of predicted reductions in ingroup shares (in %-pts.) with class placements that minimize gender consolidation compared to gender balanced/ random class placements in 809 schools with full cohort data. (DOCX) [file pone.0339581.s020.docx]

**Table S12:** Summary statistics of predicted reductions in ingroup shares (in %-pts.) with class placements that minimize gender consolidation compared to gender balanced/ random class placements in 809 schools with full cohort data

| **Group-defining variable** | **Version** | **Obs.** | **Missing values** | **Mean** | **Std. Dev.** | **Min.** | **Max.** | **Median** | **Percent increase in mean** | |
| --- | --- | --- | --- | --- | --- | --- | --- | --- | --- | --- |
| **Compared to gender balanced class placements** | | | | | | | | | |  |
| Socio-economic background | Main | 634 | 175 | 5.43 | 1.55 | -0.17 | 9.59 | 5.44 | 0 | |
|  | Full cohort, 2 classes | 639 | 170 | 7.02 | 2.62 | 0.2 | 19.15 | 6.48 | 29.46 | |
|  | Full cohort, 3 classes | 634 | 175 | 8.77 | 3.19 | 1.51 | 24.9 | 8.24 | 61.73 | |
| Educational background | Main | 628 | 181 | 4.61 | 2.01 | -0.16 | 9.4 | 4.88 | 0 | |
|  | Full cohort, 2 classes | 634 | 175 | 5.84 | 2.55 | -0.51 | 15.47 | 5.74 | 26.56 | |
|  | Full cohort, 3 classes | 624 | 185 | 7.4 | 3.47 | -0.19 | 17.67 | 7.6 | 60.51 | |
| Country of origin | Main | 530 | 279 | 3.24 | 1.9 | -0.69 | 8.68 | 3.61 | 0 | |
|  | Full cohort, 2 classes | 543 | 266 | 4.44 | 2.63 | -0.26 | 13.36 | 4.36 | 37.03 | |
|  | Full cohort, 3 classes | 518 | 291 | 5.35 | 3.36 | -0.47 | 17.55 | 5.45 | 65.44 | |
| Language | Main | 472 | 337 | 2.53 | 2.21 | -0.32 | 8.04 | 2.37 | 0 | |
|  | Full cohort, 2 classes | 515 | 294 | 3.75 | 3.09 | -0.35 | 15.17 | 3.5 | 48.08 | |
|  | Full cohort, 3 classes | 445 | 364 | 4.78 | 3.92 | -0.25 | 17.64 | 4.37 | 88.8 | |
| **Compared to random class placements** | | | | | | | | | |  |
| Socio-economic background | Main | 634 | 175 | 5.63 | 1.6 | -0.28 | 10.02 | 5.7 | 0 | |
|  | Full cohort, 2 classes | 639 | 170 | 7.21 | 2.58 | -0.15 | 19 | 6.72 | 28.05 | |
|  | Full cohort, 3 classes | 634 | 175 | 9.02 | 3.13 | 1.46 | 24.56 | 8.58 | 60.32 | |
| Educational background | Main | 628 | 181 | 4.79 | 2.05 | -0.12 | 9.82 | 5.03 | 0 | |
|  | Full cohort, 2 classes | 634 | 175 | 5.99 | 2.49 | 0.03 | 14.07 | 5.92 | 25.19 | |
|  | Full cohort, 3 classes | 624 | 185 | 7.67 | 3.35 | 0.32 | 18.4 | 7.94 | 60.33 | |
| Country of origin | Main | 530 | 279 | 3.16 | 1.9 | -0.69 | 8.23 | 3.51 | 0 | |
|  | Full cohort, 2 classes | 543 | 266 | 4.34 | 2.65 | -0.57 | 15.69 | 4.22 | 37.32 | |
|  | Full cohort, 3 classes | 518 | 291 | 5.26 | 3.31 | -0.23 | 17 | 5.09 | 66.48 | |
| Language | Main | 472 | 337 | 2.6 | 2.17 | -0.6 | 8.06 | 2.47 | 0 | |
|  | Full cohort, 2 classes | 515 | 294 | 3.73 | 3.04 | -0.46 | 15.67 | 3.45 | 43.48 | |
|  | Full cohort, 3 classes | 445 | 364 | 4.82 | 3.82 | -0.02 | 17.08 | 4.4 | 85.69 | |
| Simulation results for the first imputed PISA dataset. Information on the other imputed datasets is available on request from the authors. | | | | | | | | | |  |
