# Supplementary material for: Friendship segregation and class composition in schools: A systematic analysis of the role of attribute consolidation
Source: PLoS One. 2025 Dec 31;20(12):e0339581. doi: 10.1371/journal.pone.0339581 (PMC12755804; doi:10.1371/journal.pone.0339581)
Supplement: S13 Table — (DOCX) [file pone.0339581.s021.docx]

**Table S13:** Summary statistics of the second set of simulations based on PISA data underlying the random forests

| **Group-defining attribute** | **Variable** | **Obs.** | **Missing values** | **Mean** | **Std. Dev.** | **Min.** | **Max.** |
| --- | --- | --- | --- | --- | --- | --- | --- |
| **Socio-economic background** | Reduction ingroup shares (%-pts) (compared to random) | 14331 | 1786 | 5.09 | 1.69 | -1.57 | 10.63 |
|  | Reduction ingroup shares (%-pts) (compared to gender balanced) | 14331 | 1786 | 4.83 | 1.61 | -0.46 | 10.5 |
|  | School diversity | 16117 | 0 | 0.56 | 0.1 | 0 | 0.67 |
|  | School gender consolidation | 14466 | 1651 | 0.23 | 0.12 | 0 | 0.88 |
|  | Number of students at school | 16117 | 0 | 32.34 | 6.43 | 20 | 42 |
|  | School gender diversity | 16117 | 0 | 0.42 | 0.16 | 0 | 0.5 |
|  | Number of categories at school | 16117 | 0 | 2.95 | 0.21 | 1 | 3 |
| **Educational background** | Reduction ingroup shares (%-pts) (compared to random) | 14178 | 1939 | 4.82 | 1.97 | -1.02 | 10.42 |
|  | Reduction ingroup shares (%-pts) (compared to gender balanced) | 14178 | 1939 | 4.64 | 1.91 | -0.78 | 10.3 |
|  | School diversity | 16117 | 0 | 0.43 | 0.12 | 0 | 0.67 |
|  | School gender consolidation | 14416 | 1701 | 0.19 | 0.12 | 0 | 1 |
|  | Number of students at school | 16117 | 0 | 32.34 | 6.43 | 20 | 42 |
|  | School gender diversity | 16117 | 0 | 0.42 | 0.16 | 0 | 0.5 |
|  | Number of categories at school | 16117 | 0 | 2.35 | 0.49 | 1 | 3 |
| **Country of origin** | Reduction ingroup shares (%-pts) (compared to random) | 10304 | 5813 | 2.34 | 1.92 | -1.14 | 8.23 |
|  | Reduction ingroup shares (%-pts) (compared to gender balanced) | 10304 | 5813 | 2.43 | 1.91 | -0.76 | 8.68 |
|  | School diversity | 16117 | 0 | 0.26 | 0.21 | 0 | 0.88 |
|  | School gender consolidation | 12195 | 3922 | 0.23 | 0.13 | 0 | 1 |
|  | Number of students at school | 16117 | 0 | 32.34 | 6.43 | 20 | 42 |
|  | School gender diversity | 16117 | 0 | 0.42 | 0.16 | 0 | 0.5 |
|  | Number of categories at school | 16117 | 0 | 2.8 | 1.53 | 1 | 12 |
| **Language** | Reduction ingroup shares (%-pts) (compared to random) | 7929 | 8188 | 2.33 | 2.08 | -0.74 | 9 |
|  | Reduction ingroup shares (%-pts) (compared to gender balanced) | 7929 | 8188 | 2.32 | 2.08 | -0.64 | 8.66 |
|  | School diversity | 16117 | 0 | 0.18 | 0.18 | 0 | 0.86 |
|  | School gender consolidation | 10728 | 5389 | 0.22 | 0.12 | 0 | 1 |
|  | Number of students at school | 16117 | 0 | 32.34 | 6.43 | 20 | 42 |
|  | School gender diversity | 16117 | 0 | 0.42 | 0.16 | 0 | 0.5 |
|  | Number of categories at school | 16117 | 0 | 2.29 | 1.17 | 1 | 11 |
| Summary statistics of the variables used in the analyses of the second set of simulations in Study 2, based on the first imputed PISA dataset. Information on the other imputed datasets is available on request from the authors. | | | | | | | |
