# Supplementary material for: Friendship segregation and class composition in schools: A systematic analysis of the role of attribute consolidation
Source: PLoS One. 2025 Dec 31;20(12):e0339581. doi: 10.1371/journal.pone.0339581 (PMC12755804; doi:10.1371/journal.pone.0339581)
Supplement: S14 Table — (DOCX) [file pone.0339581.s022.docx]

**Table S14:** OLS models regressing the (average) simulated reduction in friendship segregation on school and country level predictors based on PISA data

|  | **Group-defining attribute:** | | | |
| --- | --- | --- | --- | --- |
| **Variable** | **Socio-economic background** | **Educational background** | **Country of origin** | **Language** |
| **Dependent variable:** Reduction in ingroup shares in %-pts. compared to gender-balanced class placements  **Units of analysis:** Schools | | | | |
| Intercept | 1.651*** | 0.908*** | 0.14 | -0.528*** |
|  | (10.62) | (5.3) | (1.03) | (-6.16) |
| School diversity | 5.601*** | 8.594*** | 6.744*** | 9.387*** |
|  | (18.58) | (19.17) | (12.6) | (22.39) |
| **Dependent variable:** Average reduction in ingroup shares in %-pts. compared to gender-balanced class placements  **Units of analysis:** Countries | | | | |
| Intercept | 3.012 | 3.299** | 0.463* | -0.232 |
|  | (0.86) | (2.93) | (2.36) | (-1.14) |
| Between-school segregation | 9.541 | -8.823 | 1.197 | 3.16*** |
|  | (0.36) | (-1.04) | (1.36) | (5.45) |
| Country diversity | 3.806 | 4.249 | 8.641*** | 10.265*** |
|  | (0.69) | (1.85) | (13.93) | (15.02) |
| Between-school segregation x Country diversity | -23.367 | 5.788 | -12.189*** | -14.535*** |
|  | (-0.55) | (0.34) | (-5.04) | (-8.99) |
| Number of categories | |  | -0.092*** | -0.036* |
|  | |  | (-5.88) | (-2.57) |
| **Dependent variable:** Reduction in ingroup shares in %-pts. compared to random class placements  **Units of analysis:** Schools | | | | |
| Intercept | 1.327*** | 1.367*** | 0.103 | -0.406*** |
|  | (8.6) | (7.79) | (0.79) | (-4.63) |
| School diversity | 6.641*** | 7.957*** | 6.595*** | 9.042*** |
|  | (22.57) | (17.4) | (12.57) | (20.85) |
| **Dependent Variable:** Average reduction in ingroup shares in %-pts. segregation compared to random class placements  **Units of analysis:** Countries | | | | |
| Intercept | 3.019 | 3.771*** | 0.342 | -0.168 |
|  | (0.94) | (3.52) | (1.74) | (-0.81) |
| Between-school segregation | 5.991 | -8.815 | 1.669 | 3.248*** |
|  | (0.24) | (-1.09) | (1.89) | (5.49) |
| Country diversity | 4.26 | 3.611 | 8.661*** | 10.17*** |
|  | (0.84) | (1.65) | (13.92) | (14.55) |
| Between-school segregation x Country diversity | -18.348 | 6.186 | -12.918*** | -14.531*** |
|  | (-0.47) | (0.38) | (-5.32) | (-8.8) |
| Number of categories |  |  | -0.093*** | -0.042** |
|  |  |  | (-5.93) | (-2.97) |
| Unstandardized coefficients and t-values in parentheses of OLS regressions. Models with schools as units of analysis have cluster robust standard errors. Pooled results over ten imputations using Rubin’s rules. ***p<0.001 **p<0.01 *p<0.05. %-pts. = percentage points | | | | |
