# Supplementary material for: Friendship segregation and class composition in schools: A systematic analysis of the role of attribute consolidation
Source: PLoS One. 2025 Dec 31;20(12):e0339581. doi: 10.1371/journal.pone.0339581 (PMC12755804; doi:10.1371/journal.pone.0339581)
Supplement: S15 Table — (DOCX) [file pone.0339581.s023.docx]

**Table S15**: OLS models regressing the number of friends in class, math performance and school satisfaction on consolidation (including quadratic terms)

|  |  |  | **Consolidating attribute** | | | | | | |
| --- | --- | --- | --- | --- | --- | --- | --- | --- | --- |
| **Dependent variable** | **Group-defining attribute** | **Variable** | **Socio-econ. backgr.** | **Educat. backgr.** | **Country of origin** | **Religion** | **Language** | **Resident. area** | **Gender** |
| **Friends in class** | **Socio-economic background** | Consolidation |  | 0.371 | -1.172 | 0.715 | -0.258 | 0.368 | -0.506 |
|  |  |  |  | (0.63) | (-1.55) | (0.96) | (-0.33) | (0.45) | (-0.71) |
|  |  | Consolidation^2^ |  | -0.291 | 1 | -1.055 | 0.037 | -0.624 | 0.754 |
|  |  |  |  | (-0.4) | (1.43) | (-1.01) | (0.04) | (-0.9) | (0.56) |
|  | **Educational background** | Consolidation | -0.173 |  | -0.429 | -1.607** | 0.405 | 1.04 | 0.071 |
|  |  |  | (-0.26) |  | (-0.6) | (-2.95) | (0.74) | (1.28) | (0.09) |
|  |  | Consolidation^2^ | 0.166 |  | 0.175 | 1.578** | -0.494 | -1.224 | -0.343 |
|  |  |  | (0.25) |  | (0.28) | (2.73) | (-0.9) | (-1.76) | (-0.23) |
|  | **Country of origin** | Consolidation | -0.162 | -0.394 |  | -0.243 | -0.981 | -0.324 | 0.128 |
|  |  |  | (-0.17) | (-0.61) |  | (-0.4) | (-1) | (-0.42) | (0.18) |
|  |  | Consolidation^2^ | 0.149 | 0.396 |  | -0.074 | 0.675 | 0.167 | 0.57 |
|  |  |  | (0.12) | (0.45) |  | (-0.13) | (0.93) | (0.25) | (0.41) |
|  | **Religion** | Consolidation | 0.198 | -0.852 | -0.458 |  | -0.715 | -0.708 | 0.49 |
|  |  |  | (0.19) | (-1.31) | (-0.66) |  | (-1.41) | (-0.89) | (0.9) |
|  |  | Consolidation^2^ | 0.173 | 1.434 | 0.071 |  | 0.22 | 0.238 | -0.519 |
|  |  |  | (0.12) | (1.64) | (0.12) |  | (0.46) | (0.37) | (-0.62) |
|  | **Language** | Consolidation | -0.223 | 0.072 | 0.413 | -0.217 |  | 0.605 | -0.567 |
|  |  |  | (-0.27) | (0.13) | (0.4) | (-0.43) |  | (0.84) | (-1.04) |
|  |  | Consolidation^2^ | 0.099 | -0.004 | -0.462 | -0.026 |  | -0.729 | 1.902* |
|  |  |  | (0.1) | (-0.01) | (-0.64) | (-0.06) |  | (-1.23) | (2.2) |
|  | **Residential area** | Consolidation | -1.224 | -0.894 | -0.211 | -0.31 | -0.255 |  | 0.377 |
|  |  |  | (-1.41) | (-1.17) | (-0.32) | (-0.43) | (-0.42) |  | (0.73) |
|  |  | Consolidation^2^ | 1.95 | 1.287 | -0.046 | 0.126 | 0.123 |  | -0.359 |
|  |  |  | (1.56) | (1.04) | (-0.08) | (0.14) | (0.19) |  | (-0.48) |
|  | **Gender** | Consolidation | -0.486 | -0.37 | -1.113 | 0.335 | -1.073 | 0.072 |  |
|  |  |  | (-0.56) | (-0.55) | (-1.38) | (0.57) | (-1.48) | (0.09) |  |
|  |  | Consolidation^2^ | 0.822 | 0.532 | 0.873 | -0.596 | 1.256 | -0.347 |  |
|  |  |  | (0.63) | (0.47) | (1.05) | (-0.75) | (1.49) | (-0.52) |  |
| **Math performance** | **Socio-economic background** | Consolidation |  | 0.055 | -0.486 | -0.176 | -0.132 | 0.11 | 0.081 |
|  |  |  |  | (0.23) | (-1.32) | (-0.49) | (-0.36) | (0.28) | (0.25) |
|  |  | Consolidation^2^ |  | 0.021 | 0.593 | 0.304 | 0.415 | -0.283 | -0.27 |
|  |  |  |  | (0.08) | (1.72) | (0.61) | (1.06) | (-0.84) | (-0.4) |
|  | **Educational background** | Consolidation | -0.121 |  | 0.359 | 0.237 | -0.039 | -0.353 | -0.304 |
|  |  |  | (-0.35) |  | (1.25) | (0.88) | (-0.15) | (-1.03) | (-0.93) |
|  |  | Consolidation^2^ | 0.238 |  | -0.316 | -0.427 | 0.073 | 0.22 | 0.419 |
|  |  |  | (0.69) |  | (-1.21) | (-1.3) | (0.3) | (0.75) | (0.7) |
|  | **Country of origin** | Consolidation | -0.394 | -0.141 |  | 0.04 | 0.519 | -0.138 | 0.063 |
|  |  |  | (-0.82) | (-0.54) |  | (0.12) | (1.12) | (-0.37) | (0.17) |
|  |  | Consolidation^2^ | 0.698 | 0.009 |  | -0.131 | -0.226 | 0.062 | 0.04 |
|  |  |  | (1.1) | (0.03) |  | (-0.42) | (-0.66) | (0.2) | (0.06) |
|  | **Religion** | Consolidation | 0.562 | -0.26 | -0.341 |  | -0.613* | -0.243 | -0.411 |
|  |  |  | (1.29) | (-1.03) | (-1.01) |  | (-2.33) | (-0.59) | (-1.38) |
|  |  | Consolidation^2^ | -0.696 | 0.05 | 0.193 |  | 0.436 | 0.134 | 0.593 |
|  |  |  | (-1.13) | (0.14) | (0.71) |  | (1.86) | (0.39) | (1.07) |
|  | **Language** | Consolidation | 0.65 | 0.158 | 0.312 | -0.419 |  | -0.008 | 0.444 |
|  |  |  | (1.67) | (0.67) | (0.73) | (-1.63) |  | (-0.02) | (1.59) |
|  |  | Consolidation^2^ | -0.571 | -0.223 | -0.112 | 0.313 |  | -0.002 | -0.904* |
|  |  |  | (-1.2) | (-0.84) | (-0.35) | (1.33) |  | (-0.01) | (-1.97) |
|  | **Residential area** | Consolidation | -0.583 | -0.617 | -0.475 | -0.129 | -0.172 |  | -0.467 |
|  |  |  | (-1.28) | (-1.61) | (-1.5) | (-0.38) | (-0.58) |  | (-1.67) |
|  |  | Consolidation^2^ | 0.705 | 0.84 | 0.456 | 0.021 | 0.243 |  | 0.566 |
|  |  |  | (1.09) | (1.38) | (1.58) | (0.05) | (0.82) |  | (1.42) |
|  | **Gender** | Consolidation | 0.205 | -0.308 | 0.224 | -0.336 | 0.161 | 0.187 |  |
|  |  |  | (0.49) | (-1.02) | (0.49) | (-1.14) | (0.47) | (0.5) |  |
|  |  | Consolidation^2^ | -0.372 | 0.328 | -0.181 | 0.356 | -0.22 | -0.189 |  |
|  |  |  | (-0.57) | (0.71) | (-0.42) | (0.85) | (-0.58) | (-0.62) |  |
|  |  |  |  |  |  |  |  |  |  |
|  |  |  |  |  |  |  |  |  |  |
|  |  |  |  |  |  |  |  |  |  |
|  |  |  |  |  |  |  |  |  |  |
|  |  |  |  |  |  |  |  |  |  |
|  |  |  |  |  |  |  |  |  |  |
| **School satisfaction** | **Socio-economic background** | Consolidation |  | 0.047 | -0.8 | -0.035 | -0.348 | 0.488 | 0.135 |
|  |  |  |  | (0.1) | (-1.13) | (-0.06) | (-0.47) | (0.66) | (0.23) |
|  |  | Consolidation^2^ |  | -0.051 | 0.791 | -0.006 | 0.721 | -0.736 | -0.098 |
|  |  |  |  | (-0.08) | (1.12) | (-0.01) | (0.86) | (-1.18) | (-0.09) |
|  | **Educational background** | Consolidation | -0.428 |  | -0.424 | 0.246 | 0.68 | -0.435 | -0.011 |
|  |  |  | (-0.68) |  | (-0.77) | (0.46) | (1.34) | (-0.69) | (-0.02) |
|  |  | Consolidation^2^ | 0.619 |  | 0.522 | -0.555 | -0.312 | 0.001 | -0.667 |
|  |  |  | (0.96) |  | (1.06) | (-0.96) | (-0.62) | (0) | (-0.49) |
|  | **Country of origin** | Consolidation | -0.25 | -0.244 |  | -1.183 | 0.177 | 0.556 | 0.455 |
|  |  |  | (-0.27) | (-0.49) |  | (-1.96) | (0.24) | (0.74) | (0.73) |
|  |  | Consolidation^2^ | 0.483 | 0.202 |  | 0.971 | 0.067 | -0.63 | -0.296 |
|  |  |  | (0.4) | (0.31) |  | (1.67) | (0.11) | (-1.01) | (-0.28) |
|  | **Religion** | Consolidation | -0.532 | 0.009 | -1.05 |  | -1.091* | -0.073 | -0.081 |
|  |  |  | (-0.72) | (0.02) | (-1.75) |  | (-2.32) | (-0.1) | (-0.16) |
|  |  | Consolidation^2^ | 0.972 | -0.085 | 0.809 |  | 0.928* | -0.166 | 0.829 |
|  |  |  | (0.9) | (-0.12) | (1.58) |  | (2.14) | (-0.26) | (0.96) |
|  | **Language** | Consolidation | 0.774 | 0.455 | 0.085 | -1.118* |  | 0.841 | 0.763 |
|  |  |  | (1.09) | (0.84) | (0.11) | (-2.19) |  | (1.25) | (1.39) |
|  |  | Consolidation^2^ | -0.715 | -0.707 | -0.197 | 0.83 |  | -0.842 | -1.181 |
|  |  |  | (-0.87) | (-1.12) | (-0.32) | (1.74) |  | (-1.47) | (-1.42) |
|  | **Residential area** | Consolidation | -0.581 | -0.424 | -1.091 | -0.471 | 0.253 |  | -0.041 |
|  |  |  | (-0.72) | (-0.61) | (-1.83) | (-0.68) | (0.44) |  | (-0.08) |
|  |  | Consolidation^2^ | 1.377 | 0.567 | 1.369* | 0.61 | 0.396 |  | 0.188 |
|  |  |  | (1.24) | (0.5) | (2.41) | (0.7) | (0.64) |  | (0.27) |
|  | **Gender** | Consolidation | 0.052 | 0.339 | -0.406 | -0.113 | -0.204 | -0.359 |  |
|  |  |  | (0.07) | (0.62) | (-0.54) | (-0.21) | (-0.31) | (-0.55) |  |
|  |  | Consolidation^2^ | -0.193 | -1.053 | 0.65 | 0.362 | 0.62 | 0.02 |  |
|  |  |  | (-0.16) | (-1.13) | (0.89) | (0.5) | (0.87) | (0.04) |  |
| Unstandardized coefficients and t-values in parentheses of OLS regressions with cluster robust standard errors, groups-in-survey-countries fixed effects and controlled for class size. Pooled results over ten imputations using Rubin’s rules. ***p<0.001 **p<0.01 *p<0.05. | | | | | | | | | |
