# Supplementary material for: Friendship segregation and class composition in schools: A systematic analysis of the role of attribute consolidation
Source: PLoS One. 2025 Dec 31;20(12):e0339581. doi: 10.1371/journal.pone.0339581 (PMC12755804; doi:10.1371/journal.pone.0339581)
Supplement: S16 Table — (DOCX) [file pone.0339581.s024.docx]

**Table S16:** OLS models regressing the number of friends in class, math performance and school satisfaction on consolidation (including quadratic terms) for small groups with up to four members

|  |  |  | **Consolidating attribute** | | | | | | |
| --- | --- | --- | --- | --- | --- | --- | --- | --- | --- |
| **Dependent variable** | **Group-defining attribute** | **Variable** | **Socio-econ. backgr.** | **Educat. backgr.** | **Country of origin** | **Religion** | **Language** | **Resident. area** | **Gender** |
| **Friends in class** | **Socio-economic background** | Consolidation |  | 2.794 | -1.243 | 2.448 | 0.433 | 1.269 | -2.487 |
|  |  |  |  | (1.5) | (-0.8) | (1.26) | (0.26) | (0.72) | (-1.15) |
|  |  | Consolidation^2^ |  | -2.856 | 1.452 | -3.15 | -0.623 | -0.757 | 4.736 |
|  |  |  |  | (-1.26) | (1.01) | (-1.31) | (-0.35) | (-0.5) | (1.14) |
|  | **Educational background** | Consolidation | 0.367 |  | -4.589* | -5.662* | -2.151 | 2.757 | -0.011 |
|  |  |  | (0.17) |  | (-2.21) | (-2.5) | (-1.26) | (1.32) | (0) |
|  |  | Consolidation^2^ | -0.502 |  | 3.594* | 6.57* | 2.358 | -3.305 | -1.607 |
|  |  |  | (-0.24) |  | (1.98) | (2.28) | (1.47) | (-1.9) | (-0.26) |
|  | **Country of origin** | Consolidation | 0.898 | -0.571 |  | -1.017 | -5.527* | -3.496* | 1.492 |
|  |  |  | (0.4) | (-0.25) |  | (-0.52) | (-2.08) | (-2.12) | (0.69) |
|  |  | Consolidation^2^ | -1.395 | 1.063 |  | 0.289 | 3.999* | 2.783 | -2.816 |
|  |  |  | (-0.49) | (0.46) |  | (0.17) | (2.11) | (1.87) | (-0.66) |
|  | **Religion** | Consolidation | 0.614 | -1.257 | 0.548 |  | -0.632 | -1.41 | 0.805 |
|  |  |  | (0.21) | (-0.84) | (0.35) |  | (-0.54) | (-0.62) | (0.56) |
|  |  | Consolidation^2^ | -0.436 | 2 | -0.937 |  | 0.087 | 0.579 | -1.383 |
|  |  |  | (-0.11) | (1.08) | (-0.67) |  | (0.08) | (0.33) | (-0.69) |
|  | **Language** | Consolidation | -0.514 | -2.565 | -6.878 | -1.103 |  | 0.901 | 0.657 |
|  |  |  | (-0.14) | (-1.01) | (-1.26) | (-0.51) |  | (0.39) | (0.29) |
|  |  | Consolidation^2^ | -1.227 | 4.538 | 3.881 | 0.494 |  | -1.265 | -0.794 |
|  |  |  | (-0.25) | (1.57) | (1.11) | (0.28) |  | (-0.63) | (-0.21) |
|  | **Residential area** | Consolidation | -1.504 | -2.136 | -0.003 | -0.325 | -0.323 |  | 0.683 |
|  |  |  | (-1.08) | (-1.44) | (0) | (-0.24) | (-0.31) |  | (0.65) |
|  |  | Consolidation^2^ | 1.775 | 2.525 | -0.556 | -0.168 | -0.004 |  | -0.421 |
|  |  |  | (0.95) | (1.17) | (-0.55) | (-0.1) | (0) |  | (-0.24) |
|  | **Gender** | Consolidation | 1.689 | 3.408 | -3.8 | -2.329 | -4.369 | 1.408 |  |
|  |  |  | (0.33) | (0.34) | (-0.95) | (-0.97) | (-1.04) | (0.34) |  |
|  |  | Consolidation^2^ | -0.982 | -8.521 | 2.872 | 3.225 | 4.545 | -1.733 |  |
|  |  |  | (-0.1) | (-0.45) | (0.78) | (1.3) | (1.04) | (-0.51) |  |
| **Math performance** | **Socio-economic background** | Consolidation |  | -0.026 | -0.404 | -0.345 | 0.144 | -0.234 | 0.74 |
|  |  |  |  | (-0.03) | (-0.6) | (-0.38) | (0.19) | (-0.29) | (0.62) |
|  |  | Consolidation^2^ |  | -0.136 | 0.674 | 0.516 | 0.232 | -0.134 | -1.881 |
|  |  |  |  | (-0.13) | (1.07) | (0.45) | (0.29) | (-0.19) | (-0.82) |
|  | **Educational background** | Consolidation | -0.873 |  | -0.149 | 0.392 | -0.625 | -1.895* | 0.098 |
|  |  |  | (-0.82) |  | (-0.19) | (0.44) | (-0.83) | (-2.15) | (0.08) |
|  |  | Consolidation^2^ | 0.827 |  | 0.175 | -1.069 | 0.756 | 1.572* | -0.723 |
|  |  |  | (0.75) |  | (0.24) | (-0.94) | (1.01) | (2.05) | (-0.28) |
|  | **Country of origin** | Consolidation | -0.283 | -0.013 |  | 0.928 | 0.778 | 0.593 | 0.589 |
|  |  |  | (-0.2) | (-0.01) |  | (0.98) | (0.54) | (0.73) | (0.54) |
|  |  | Consolidation^2^ | 0.564 | -0.313 |  | -0.622 | -0.157 | -0.517 | -1.242 |
|  |  |  | (0.31) | (-0.32) |  | (-0.73) | (-0.15) | (-0.71) | (-0.59) |
|  | **Religion** | Consolidation | 0.464 | -0.777 | -1.781* |  | -1.95** | -0.227 | 0.586 |
|  |  |  | (0.31) | (-1.09) | (-2.08) |  | (-3.12) | (-0.22) | (0.88) |
|  |  | Consolidation^2^ | -0.739 | 0.729 | 1.489* |  | 1.641** | 0.19 | -1.313 |
|  |  |  | (-0.39) | (0.77) | (2.11) |  | (3) | (0.23) | (-1.26) |
|  | **Language** | Consolidation | 1.629 | 0.358 | -0.182 | 0.45 |  | 0.854 | -0.305 |
|  |  |  | (1.03) | (0.31) | (-0.08) | (0.43) |  | (1.03) | (-0.27) |
|  |  | Consolidation^2^ | -1.217 | -0.438 | 0.26 | -0.216 |  | -0.285 | 0.281 |
|  |  |  | (-0.56) | (-0.34) | (0.17) | (-0.24) |  | (-0.39) | (0.14) |
|  | **Residential area** | Consolidation | -0.564 | -0.187 | -0.852 | -0.545 | -0.543 |  | -0.119 |
|  |  |  | (-0.72) | (-0.24) | (-1.71) | (-0.85) | (-1.1) |  | (-0.2) |
|  |  | Consolidation^2^ | 0.911 | 0.361 | 0.829 | 0.634 | 0.683 |  | -0.122 |
|  |  |  | (0.89) | (0.32) | (1.64) | (0.8) | (1.34) |  | (-0.12) |
|  | **Gender** | Consolidation | 4.213 | -1.095 | -1.541 | 0.05 | -0.924 | -1.924 |  |
|  |  |  | (1.32) | (-0.17) | (-0.61) | (0.03) | (-0.34) | (-1.01) |  |
|  |  | Consolidation^2^ | -8.799 | 2.942 | 1.656 | 0.037 | 1.019 | 1.307 |  |
|  |  |  | (-1.39) | (0.24) | (0.75) | (0.02) | (0.39) | (0.78) |  |
|  |  |  |  |  |  |  |  |  |  |
|  |  |  |  |  |  |  |  |  |  |
|  |  |  |  |  |  |  |  |  |  |
|  |  |  |  |  |  |  |  |  |  |
|  |  |  |  |  |  |  |  |  |  |
| **School satisfaction** | **Socio-economic background** | Consolidation |  | -0.647 | -0.672 | 0.815 | -1.207 | 0.965 | 0.318 |
|  |  |  |  | (-0.35) | (-0.46) | (0.48) | (-0.74) | (0.6) | (0.14) |
|  |  | Consolidation^2^ |  | 0.821 | 0.382 | -1.064 | 1.3 | -0.961 | -0.646 |
|  |  |  |  | (0.36) | (0.27) | (-0.5) | (0.75) | (-0.69) | (-0.14) |
|  | **Educational background** | Consolidation | -0.285 |  | -2.808 | 3.124 | 0.446 | -2.332 | -1.15 |
|  |  |  | (-0.13) |  | (-1.65) | (1.36) | (0.24) | (-1.25) | (-0.43) |
|  |  | Consolidation^2^ | 0.914 |  | 2.906 | -4.386 | 0.723 | 1.366 | -0.427 |
|  |  |  | (0.43) |  | (1.91) | (-1.48) | (0.42) | (0.82) | (-0.08) |
|  | **Country of origin** | Consolidation | 0.294 | -1.565 |  | -0.639 | 2.316 | 1.824 | -0.61 |
|  |  |  | (0.11) | (-0.85) |  | (-0.36) | (1.05) | (1.1) | (-0.36) |
|  |  | Consolidation^2^ | 0.111 | 1.126 |  | 0.639 | -1.207 | -1.917 | 2.943 |
|  |  |  | (0.03) | (0.55) |  | (0.4) | (-0.75) | (-1.32) | (1.12) |
|  | **Religion** | Consolidation | -1.472 | 1.117 | -2.103 |  | -2.537* | -0.349 | -0.422 |
|  |  |  | (-0.59) | (0.78) | (-1.32) |  | (-2.09) | (-0.17) | (-0.3) |
|  |  | Consolidation^2^ | 2.733 | -1.828 | 1.537 |  | 2.33* | -0.034 | 1.925 |
|  |  |  | (0.8) | (-1.04) | (1.13) |  | (2.17) | (-0.02) | (0.87) |
|  | **Language** | Consolidation | 1.424 | -0.099 | -2.259 | -1.862 |  | 2.743 | 2.679 |
|  |  |  | (0.46) | (-0.04) | (-0.6) | (-0.84) |  | (1.31) | (1.29) |
|  |  | Consolidation^2^ | -0.503 | -0.412 | 0.867 | 1.688 |  | -2.356 | -4.31 |
|  |  |  | (-0.11) | (-0.16) | (0.33) | (0.83) |  | (-1.21) | (-1.27) |
|  | **Residential area** | Consolidation | 0.987 | -0.157 | -2.188* | -1.1 | -0.576 |  | -0.294 |
|  |  |  | (0.71) | (-0.11) | (-2.25) | (-0.92) | (-0.61) |  | (-0.26) |
|  |  | Consolidation^2^ | -0.429 | 0.352 | 2.175* | 0.965 | 1.268 |  | 1.091 |
|  |  |  | (-0.22) | (0.18) | (2.2) | (0.67) | (1.19) |  | (0.6) |
|  | **Gender** | Consolidation | 9.345 | 7.984 | -1.45 | 1.643 | -6.971 | 2.835 |  |
|  |  |  | (1.72) | (0.86) | (-0.29) | (0.61) | (-1.51) | (0.94) |  |
|  |  | Consolidation^2^ | -17.172 | -15.227 | 0.703 | -1.422 | 6.501 | -3.101 |  |
|  |  |  | (-1.6) | (-0.89) | (0.16) | (-0.51) | (1.38) | (-1.21) |  |
| Unstandardized coefficients and t-values in parentheses of OLS regressions with cluster robust standard errors, groups-in-survey-countries fixed effects and controlled for class size. Pooled results over ten imputations using Rubin’s rules. ***p<0.001 **p<0.01 *p<0.05. | | | | | | | | | |
