# Supplementary material for: Friendship segregation and class composition in schools: A systematic analysis of the role of attribute consolidation
Source: PLoS One. 2025 Dec 31;20(12):e0339581. doi: 10.1371/journal.pone.0339581 (PMC12755804; doi:10.1371/journal.pone.0339581)
Supplement: S17 Table — (DOCX) [file pone.0339581.s025.docx]

**Table S17:** Missingness and number of categories in the four group-defining attributes in PISA

|  |  |  | **Categories per class** | | | |
| --- | --- | --- | --- | --- | --- | --- |
| **Group-defining attribute** | **Obs.** | **Missing values** | **Mean** | **Std. Dev.** | **Min.** | **Max.** |
| Socio-economic background | 482506 | 38704 | 2.95 | 0.21 | 1 | 3 |
| Educational background | 506789 | 14421 | 2.35 | 0.49 | 1 | 3 |
| Country of origin | 510322 | 10888 | 2.8 | 1.53 | 1 | 12 |
| Language | 509092 | 12118 | 2.29 | 1.17 | 1 | 11 |
| Missing values are imputed ten times using multivariate imputation by chained equations. The summary statistics on the number of categories per class are based on the first imputed dataset. Information on the other imputed datasets is available on request from the authors. | | | | | | |
