# Supplementary material for: Friendship segregation and class composition in schools: A systematic analysis of the role of attribute consolidation
Source: PLoS One. 2025 Dec 31;20(12):e0339581. doi: 10.1371/journal.pone.0339581 (PMC12755804; doi:10.1371/journal.pone.0339581)
Supplement: S18 Table — (DOCX) [file pone.0339581.s026.docx]

Table S18: All survey questions used for the analyses

| Variable | Question | Response categories | Survey | Position in survey |
| --- | --- | --- | --- | --- |
| CILS4EU |  |  |  |  |
| Gender | Are you a boy or a girl? | - Boy - Girl | CILS4EU, Main questionnaire | 1 |
| Country of origin | In which country were you born? | England:   - Britain - Bangladesh - China - India - Jamaica - Nigeria - Pakistan - Turkey - Other country  🡪 Please specify   Germany:   - Germany - Italy - Poland - Russia - Turkey - Other country 🡪 Please specify:   Netherlands:   - Netherlands - Turkey - Morocco - Surinam - Netherlands Antilles/Aruba - Other country 🡪 Please specify:   Sweden:   - Sweden 🡪 Go to 5 - Other country 🡪 Please specify: | CILS4EU, Main questionnaire | 3 |
| Language spoken at home | Is there a language other than [survey country language] spoken at your home? | - Yes - No | CILS4EU, Main questionnaire | 7 |
| Language spoken at home | Which language is this? | England:   - Punjabi - Gujerati - Bengali - Urdu - Hindi - Cantonese - Turkish - Other language 🡪 Please specify:   Germany:   - Italian - Polish - Russian - Turkish - Other language 🡪 Please specify:   Netherlands:   - Turkish - Arabic - Berbers - English - Hindi/Hindustani - Other language 🡪 Please specify:   Sweden:   - Which language is this? | CILS4EU, Main questionnaire | 8 |
| Language spoken at home | In this (second) language, how often do you talk to your family? | - Always - Often - Sometimes - Never | CILS4EU, Main questionnaire | 10 |
| Math performance | How well are you doing in the following subjects? Math | - Very well - Quite well - OK - Not that well - Not well at all | CILS4EU, Main questionnaire | 16 |
| Country of origin | The following questions are about your biological mother. If she is no longer alive or if you are not in touch  with her, please answer the questions as best as you can.  In which country was your biological mother born? | England:   - Britain - Bangladesh - China - India - Jamaica - Nigeria - Pakistan - Turkey - Other country  🡪 Please specify - I don’t know the country   Germany:   - Germany - Italy - Poland - Russia - Turkey - Other country 🡪 Please specify: - I don’t know the country   Netherlands:   - Netherlands - Turkey - Morocco - Surinam - Netherlands Antilles/Aruba - Indonesia - Other country 🡪 Please specify: - I don’t know the country   Sweden:   - Sweden - Other country 🡪 Please specify: - I don’t know the country | CILS4EU, Main questionnaire | 25 |
| Educational background | Did your mother complete primary school (or a similar foreign education)? | - Yes - No - Don’t know | CILS4EU, Main questionnaire | 27 |
| Educational background | Did your mother complete secondary school (or a similar foreign education)? | - Yes - No - Don’t know | CILS4EU, Main questionnaire | 27 |
| Educational background | Did your mother complete university? | - Yes - No - Don’t know | CILS4EU, Main questionnaire | 27 |
| Socio-economic background | Think about your mother’s job. If she is not currently working, think about her last job. What is the name of her job? Additionally, please describe what she does in her job. | - *open* - She has never worked before. | CILS4EU, Main questionnaire | 28 |
|  | The following questions are about your biological father. If he is no longer alive or if you are not in touch with him, please answer the questions as best as you can.  In which country was your biological father born? | England:   - Britain - Bangladesh - China - India - Jamaica - Nigeria - Pakistan - Turkey - Other country  🡪 Please specify - I don’t know the country   Germany:   - Germany - Italy - Poland - Russia - Turkey - Other country 🡪 Please specify: - I don’t know the country   Netherlands:   - Netherlands - Turkey - Morocco - Surinam - Netherlands Antilles/Aruba - Indonesia - Other country 🡪 Please specify: - I don’t know the country   Sweden:   - Sweden - Other country 🡪 Please specify: - I don’t know the country | CILS4EU, Main questionnaire | 31 |
| Educational background | Did your father complete primary school (or a similar foreign education)? | - Yes - No - Don’t know | CILS4EU, Main questionnaire | 33 |
| Educational background | Did your father complete secondary school (or a similar foreign education)? | - Yes - No - Don’t know | CILS4EU, Main questionnaire | 33 |
| Educational background | Did your father complete university? | - Yes - No - Don’t know | CILS4EU, Main questionnaire | 33 |
| Socio-economic background | Think about your father’s job. If he is not currently working, think about his last job. What is the name of his job? Additionally, please describe what he does in his job. | - *open* - He has never worked before. | CILS4EU, Main questionnaire | 34 |
| School satisfaction | On a scale from 1 to 10 where 1 is very unsatisfied and 10 is very satisfied, how satisfied are you with school in general? | - Very unsatisfied 1 - 2 - 3 - 4 - 5 - 6 - 7 - 8 - 9 - Very satisfied 10 | CILS4EU, Main questionnaire | 51 |
| Religion | What is your religion? | England:   - No religion - Buddhism - Christianity - Hinduism - Judaism - Islam - Sikh - Other religion 🡪 Please specify:   Germany and Netherlands:   - No religion - Buddhism - Christianity: Catholic - Christianity: Protestant - Hinduism - Islam - Judaism - Other religion 🡪 Please specify:   Sweden:   - No religion - Buddhism - Christianity - Hinduism - Islam - Judaism - Other religion 🡪 Please specify: | CILS4EU, Main questionnaire | 71 |
| Friends in class | Here are some questions about your friends. You can answer these questions for one to five friends. You should not count your boyfriend/girlfriend.  Does he/she go to your school? | - Yes, same class - Yes, but different class - No, goes to another school - No, has finished schooling | CILS4EU, Friends questionnaire | 5 |
| Share of ingroup friends | Who are your best friends in class? Here you may write down no more than five numbers. |  | CILS4EU, Sociometric questionnaire | 1 |
| Share of ingroup friends | Who is your best friend in class? Here you may write down no more than one number. |  | CILS4EU, Sociometric questionnaire | 2 |
| Residential area | Which classmates live within 5 minute walk from your home? |  | CILS4EU, Sociometric questionnaire | 9 |
| Country of origin | In which country were you born? | England:   - Britain - Bangladesh - China - India - Jamaica - Nigeria - Pakistan - Turkey - Other country 🡪 Please specify:   Germany:   - Germany - Italy - Poland - Russia - Turkey - Other country 🡪 Please specify:   Netherlands:   - Netherlands - Turkey - Morocco - Surinam - Netherlands Antilles/Aruba - Indonesia - Other country 🡪 Please specify:   Sweden:   - Sweden - Other country 🡪 Please specify: | CILS4EU, Parents questionnaire | 25 |
| Educational background | What is your highest level of education? If you got your degree outside <survey country>, please select the <survey country> level that best matches your foreign degree. | England:   - I don't have a school leaving certificate - GCSEs or a similar qualification - A Levels or a similar qualification - University degree   Germany and Sweden:   - I don't have a school leaving certificate - Degree below upper secondary school - Degree from upper secondary school - University degree   Netherlands:   - No education - Primary school - Secondary school - Lower vocational education - Higher vocational education - University | CILS4EU, Parents questionnaire | 37 |
| Socio-economic background | What is your job title? Additionally, please describe what you do in your job. |  | CILS4EU, Parents questionnaire | 44 |
| Country of origin | In which country was your partner/husband/wife born? | England:   - Britain - Bangladesh - China - India - Jamaica - Nigeria - Pakistan - Turkey - Other country 🡪 Please specify:   Germany:   - Germany - Italy - Poland - Russia - Turkey - Other country 🡪 Please specify:   Netherlands:   - Netherlands - Turkey - Morocco - Surinam - Netherlands Antilles/Aruba - Indonesia - Other country 🡪 Please specify:   Sweden:   - Sweden - Other country 🡪 Please specify: | CILS4EU, Parents questionnaire | 54 |
| Educational background | What is partner's/husband's/wife's highest level of education? If he/she got his/her degree outside <survey country>, please select the <survey country> level that best matches his/her foreign degree. | England:   - He/she doesn’t have a school leaving certificate - GCSEs or a similar qualification - A Levels or a similar qualification - University degree   Germany and Sweden:   - He/she doesn’t have a school leaving certificate - Degree below upper secondary school - Degree from upper secondary school - University degree   Netherlands:   - No education - Primary school - Secondary school - Lower vocational education - Higher vocational education - University | CILS4EU, Parents questionnaire | 60 |
| Socio-economic background | What is his/her job title? Additionally, please describe what he/she does in his/her job. |  | CILS4EU, Parents questionnaire | 66 |
|  |  |  | CILS4EU, Parents questionnaire |  |
| PISA 2018 |  |  |  |  |
| Gender | Are you female or male? | - Female - Male | PISA 2018, Student questionnaire | 4 |
| Educational background | What is the <highest level of schooling> completed by your mother? | - <ISCED level 3A> - <ISCED level 3B, 3C> - <ISCED level 2> - <ISCED level 1> - She did not complete - <ISCED level 1> | PISA 2018, Student questionnaire | 5 |
| Educational background | Does your mother have any of the following qualifications? | - <ISCED level 6> - <ISCED level 5A> - <ISCED level 5B> - <ISCED level 4> | PISA 2018, Student questionnaire | 6 |
| Educational background | What is the <highest level of schooling> completed by your father? | - <ISCED level 3A> - <ISCED level 3B, 3C> - <ISCED level 2> - <ISCED level 1> - He did not complete - <ISCED level 1> | PISA 2018, Student questionnaire | 7 |
| Educational background | Does your father have any of the following qualifications? | - <ISCED level 6> - <ISCED level 5A> - <ISCED level 5B> - <ISCED level 4> | PISA 2018, Student questionnaire | 8 |
| Socio-economic background | The following two questions concern your mother’s job:  *(If she is not working now, please tell us her last main job.)*  What is your mother’s main job? (e.g. school teacher, kitchen-hand, sales manager)  *Please type in the job title.*  What does your mother do in her main job? (e.g. teaches high school students, helps the cook prepare meals in a restaurant, manages a sales team)  *Please use a sentence to describe the kind of work she does or did in that job.* |  | PISA 2018, Student questionnaire | 14 |
| Socio-economic background | The following two questions concern your father’s job:  *(If he is not working now, please tell us his last main job.)*  What is your father’s main job? (e.g. school teacher, kitchen-hand, sales manager)  *Please type in the job title.*  What does your father do in his main job? (e.g. teaches high school students, helps the cook prepare meals in a restaurant, manages a sales team)  *Please use a sentence to describe the kind of work he does or did in that job.* |  | PISA 2018, Student questionnaire | 15 |
| Country of origin | In what country were you and your parents born?  You  Mother  Father | - <Country A> - <Country B> - <Country C> - <Country D> - <…etc.> - Other country | PISA 2018, Student questionnaire | 19 |
| Language spoken at home | What language do you speak at home most of the time? | - <Language 1> - <Language 2> - <Language 3> - <…etc.> - Other language | PISA 2018, Student questionnaire | 22 |
| Survey questions in the order they appear in the questionnaires. The three student questionnaires in CILS4EU were administered in the same order as shown in the table (main, friends, sociometric questionnaire). | | | | |
